# Artificial intelligence for automated ICD-10 coding: a systematic review of multi-label text classification in clinical narratives

**DOI:** 10.3389/fdgth.2026.1905379

**Published:** 2026-08-13

**Authors:** Kamonrat Tangudomkit, Sawrawit Chairat, Sitthichok Chaichulee

**Affiliations:** Department of Biomedical Sciences and Biomedical Engineering, Faculty of Medicine, Prince of Songkla University, Songkhla, Thailand

**Keywords:** automatic ICD-10 coding, class imbalance, clinical natural language processing, deep learning, explainability, large language models, multi-label text classification, systematic review

## Abstract

**Background:**

ICD-10 coding is an essential process in healthcare systems that supports clinical management, reimbursement, and health data analytics. However, the complexity of its hierarchical structure and the large number of available codes make manual coding limited in terms of time, cost, and consistency. Despite growing research in this area, evidence remains fragmented, particularly regarding real-world implementation readiness.

**Objective:**

To review and synthesize existing knowledge on algorithms, datasets, evaluation methods, and real-world implementation readiness of automatic ICD-10 coding systems.

**Methods:**

Eligible studies were original research articles, preprints, or conference papers published in English between January 1, 2020 and December 31, 2025, and retrieved from seven academic databases: Scopus, PubMed, Web of Science, IEEE Xplore, ACM Digital Library, arXiv, and Google Scholar. Studies were included if they investigated automatic ICD-10 coding from clinical text using machine learning, deep learning, transformer-based, or large language model (LLM) approaches. Methodological quality was assessed using a research-question-driven appraisal framework. This systematic review followed PRISMA 2020 guidance and was preregistered in the Open Science Framework (OSF) at https://osf.io/cegqk.

**Results:**

A total of 257 records were identified, of which 24 studies met the inclusion criteria and contributed 296 experimental evaluations overall. Study quality was high in 7 studies, moderate in 8, and limited by technical or methodological concerns in 9. Hybrid deep learning (Hybrid DL) was most often used as the main automated coding approach, while machine learning (ML) and rule-based approaches were mainly used as baselines. F1-macro was consistently lower than F1-micro among studies reporting both metrics. Hybrid DL showed the most stable performance under all-code or full-code evaluation, while AI model performance varied by the documents-per-label (D/L) ratio.

**Discussion:**

The evidence indicates continued technical progress, particularly through Hybrid DL and transformer-based approaches, while LLM-based methods remain emerging and less consistently effective for structured multi-label coding. The observed D/L–performance relationship suggested that AI model selection should consider dataset structure and label support, in addition to algorithmic complexity.

**Conclusion:**

AI-based automatic ICD-10 coding is a promising approach for clinical coding support. Future research should prioritize rare-label imbalance, reproducibility, explainability, and validation across diverse clinical settings.

**Systematic Review Registration:**

https://osf.io/cegqk.

## 1 Introduction

Disease coding is the assignment of standardized codes to clinical diagnoses and related health conditions. It is usually done by trained clinical coders based on clinical documentation written by physicians and other healthcare professionals. This documentation may include both explicit clinical diagnoses that can be directly mapped to ICD-10 codes and broader clinical information, such as symptoms, medical history, physical findings, laboratory results, procedures, and discharge summaries, which may help distinguish the correct code from several plausible alternatives (1). It is a routine part of hospital and public health information systems that supports patient record management, reimbursement, health service planning, and medical research. Without standardized coding, diagnostic reporting becomes less consistent and more prone to administrative and analytical errors (2, 3). In practice, disease code assignment remains challenging. Clinical narratives may contain ambiguous terms, inconsistent phrasing, and incomplete information. Coders need to work with a large and detailed coding system and follow specific coding guidelines and assignment criteria (3, 4).

The International Statistical Classification of Diseases and Related Health Problems (ICD), maintained by the World Health Organization (WHO) (2, 5), is the main international system used to classify diseases and related health conditions. This classification has been revised over time, and ICD-11 is the most recent version. However, adoption of ICD-11 remains limited. Most operational hospital coding systems, reimbursement infrastructures, and publicly available clinical research datasets are still built on ICD-10. For this reason, ICD-10 remains the practical target for automated coding research and is the focus of this review (3, 6, 7).

**Figure 1:**
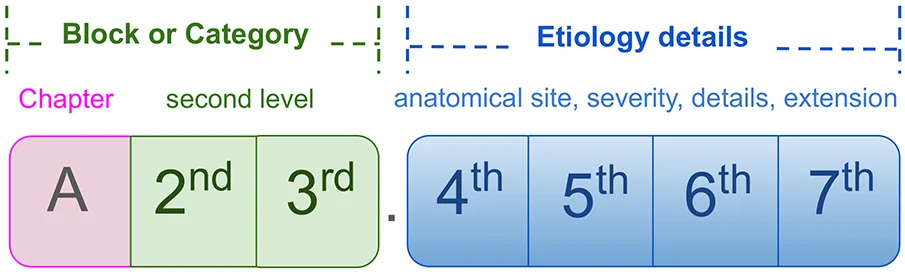
Hierarchical organization of ICD-10 codes with progression from broad chapters (first character) to blocks or categories (first three characters), and more specific subcategories or full codes.

ICD-10 is organized as a hierarchical system (see Figure 1). At the highest level, ICD-10 is divided into chapters which group diseases by organ system or cause. Each chapter is divided into blocks that group conditions with similar characteristics. Within each block are categories, represented by three-character codes that provide more specific diagnostic classification. These categories can be divided into subcategories to capture clinical details, such as anatomical site, severity, or etiology. This hierarchical structure allows coding at different levels of detail, but also makes code assignment more complex. Compared with ICD-9, ICD-10 has a larger and more detailed coding structure, and ICD-11 further increases specificity through a flexible coding architecture. This expanded structure has been associated with increased coding time, greater demands on coder expertise, and higher training requirements across institutions (3, 5).

Since manual coding depends heavily on individual expertise, automatic ICD-10 coding systems have been developed to support code assignment and to potentially reduce coder workload and inconsistency. Early studies mainly used rule-based methods. As AI methods advanced, machine learning (ML) and deep learning (DL) approaches were adopted, followed more recently by large language models (LLMs) (2, 3, 5, 7). Despite this progress, evidence on these systems remains fragmented. Most prior work emphasizes AI model performance, while evaluation design, reproducibility, transparency, and real-world implementation readiness have received far less systematic attention. This review addresses that gap by synthesizing not only AI-based coding approaches and performance, but also how automatic ICD-10 coding systems are evaluated and whether they report features relevant to clinical implementation (6, 8).

To this end, we conduct a systematic review of automatic ICD-10 coding research published between 2020 and 2025, following PRISMA 2020 (9). We synthesize AI model architectures, dataset characteristics, preprocessing and evaluation practices, and class-imbalance handling, and we appraise study quality with particular attention to transparency, reproducibility, and generalizability. The review is organized around eleven research questions, defined below, that together characterize both the technical state of the field and its readiness for real-world deployment.

## 2 Previous literature reviews and identified research gaps

Recent reviews show that automatic ICD coding research has developed from traditional ML methods toward DL, transformer-based models, hybrid architectures, and more recently LLMs (2, 3, 5, 7). Among recent systematic reviews, Kaur et al. (3) examined studies published between 2010 and 2021 and found that most included studies focused on ICD-9-CM, with many using MIMIC-II or MIMIC-III datasets. The review identified a shift from traditional ML to DL methods, including convolutional neural networks (CNNs), recurrent neural networks (RNNs), long short-term memory networks (LSTMs), gated recurrent units (GRUs), and transformer-based models, with increasing use of BERT-family models and domain-specific pretrained language models (PLMs). This review also noted emerging interest in hierarchical models, prompt-based approaches, LLM-assisted coding, and the role of clinical coders.

Several reviews have focused more specifically on DL-based automatic ICD coding. Teng et al. (5) focused mainly on DL architectures and reported that DL generally performed better than traditional ML in learning semantic information from clinical free text without extensive feature engineering. Li et al. (2) provided a broad synthesis of DL research and described a progression from CNN- and RNN-based models to attention-based and transformer-based architectures, with transformer models and PLMs often performing well for long and complex clinical text. Ji et al. (10) presented a unified framework for DL-based medical coding, covering encoders, deep architectures, decoders, and auxiliary information including AI models such as CNN, BERT-XML, MultiResCNN, CAML, and HyperCore, along with open-source benchmarking tools such as AnEMIC.

Other recent reviews have expanded the discussion beyond conventional DL model comparison. Mousavi Baigi et al. (6) focused on system-level and policy-related aspects of AI-based ICD coding, emphasizing transparent, auditable, and human-AI collaborative systems for sustainable healthcare implementation. Gershon et al. (7) reviewed the use of LLMs, including BERT and GPT, for automatic ICD coding and found that these models performed better on frequent codes than on rare codes. The review also highlighted possible roles for LLMs in data augmentation, retrieval-based approaches, and graph-based decoders for handling rare codes.

Across these reviews, several recurring challenges remain (2, 3, 5, 7). ICD coding is consistently described as a large-scale multi-label problem with a long-tail label distribution, where rare codes are difficult to predict. Many studies simplify the task by using top-k prediction, reduced code granularity, or exclusion of infrequent labels. Most prior reviews also focus mainly on algorithms and AI model performance, while giving less attention to implementation-related issues such as human-in-the-loop workflows, explainability, reproducibility, external validation, and generalizability. In addition, recent studies include heterogeneous ICD-related tasks, including full-code assignment, restricted code prediction, coarse-granularity prediction, entity linking, and human-AI coding workflows. These differences make direct comparison of reported AI model performance difficult (3, 7) and highlight the need for a review that synthesizes both technical approaches and implementation-related reporting features.

### 2.1 Our contributions

This review aims to provide practical guidance for researchers developing automatic ICD-10 coding systems. Specifically, it systematically synthesizes the methods and performance of AI models reported in previous studies. It also examines how dataset characteristics are related to AI model performance, so that researchers can better anticipate expected results before implementation. In addition, this review addresses gaps identified in earlier reviews by appraising research transparency, reproducibility, and generalizability using a structured quality rubric informed by established AI reporting and risk-of-bias frameworks for prediction-model studies.

Based on these objectives, the research questions are formulated as follows:

- **RQ1**: What is the overall publication landscape from 2020 to 2025, and which types of AI models have been investigated?
- **RQ2**: At what stage are most studies conducted, how are experiments designed, and what testing settings are commonly used for AI model evaluation?
- **RQ3**: What is the overall methodological quality of the included studies across key criteria, including dataset assurance, explainability, code availability, reproducibility, transparency, and generalizability?
- **RQ4**: What are the main characteristics of the datasets and code-set sizes used in the included studies?
- **RQ5**: Which text preprocessing and feature representation techniques are most commonly used?
- **RQ6**: Which AI models are most often selected as the main automated coding approaches, and which are most often used as baseline comparators?
- **RQ7**: Which hyperparameters and fine-tuning details are most commonly reported?
- **RQ8**: Which evaluation metrics and evaluation protocols are most commonly used?
- **RQ9**: How are F-measure metrics reported across different AI model types and evaluation settings?
- **RQ10**: What label-support patterns and imbalance-handling strategies are reported, and how are these associated with reported performance?
- **RQ11**: How does the relationship between documents per label (D/L) and AI model type influence performance?

## 3 Theoretical and conceptual framework

In routine care, a single patient encounter can generate many types of information in the electronic health record (EHR), including clinical documentation, laboratory results, vital signs, imaging findings, and pathology reports. After the encounter, the documented diagnoses and related health conditions need to be translated into ICD-10 codes for reporting and administrative use. Because more than one diagnosis may be recorded in the same case, more than one ICD-10 code may also be assigned. Moreover, ICD-10 is implemented in multiple national or regional versions, such as ICD-10-CM, ICD-10-AM, and ICD-10-TM, which preserve the core ICD-10 structure but differ in code granularity, terminology, procedure coding components, and local implementation rules. These differences are important in automatic ICD coding studies because models trained on one ICD-10 version may not transfer directly to datasets coded with another version (11, 12). This section outlines the conceptual framework used to organize the review: the multi-label classification setup, the end-to-end coding pipeline, the framework for handling class imbalance, and the categories of explainability methods relevant to clinical AI (8, 13).

### 3.1 Multi-label text classification

Multi-label text classification is a classification setting in which a single text instance can be assigned more than one label at the same time, unlike multi-class classification, where each instance is assigned to only one class (14). In automatic ICD-10 coding, the input is usually a text-based clinical document from the EHR, such as a discharge summary or an outpatient note. In some cases, the input is constructed by combining multiple sources of clinical documentation generated during the same patient visit into a single input for prediction. Because one document or one encounter-level input may contain more than one diagnosis or comorbidity, automatic ICD-10 coding is treated as a large-scale multi-label classification problem with a long-tail label distribution (4). In many multi-label models, each label is treated independently, and final label assignment is based on predicted scores or probabilities, often using threshold values selected for the task (15).

### 3.2 Automatic ICD-10 coding pipeline

**Figure 2:**
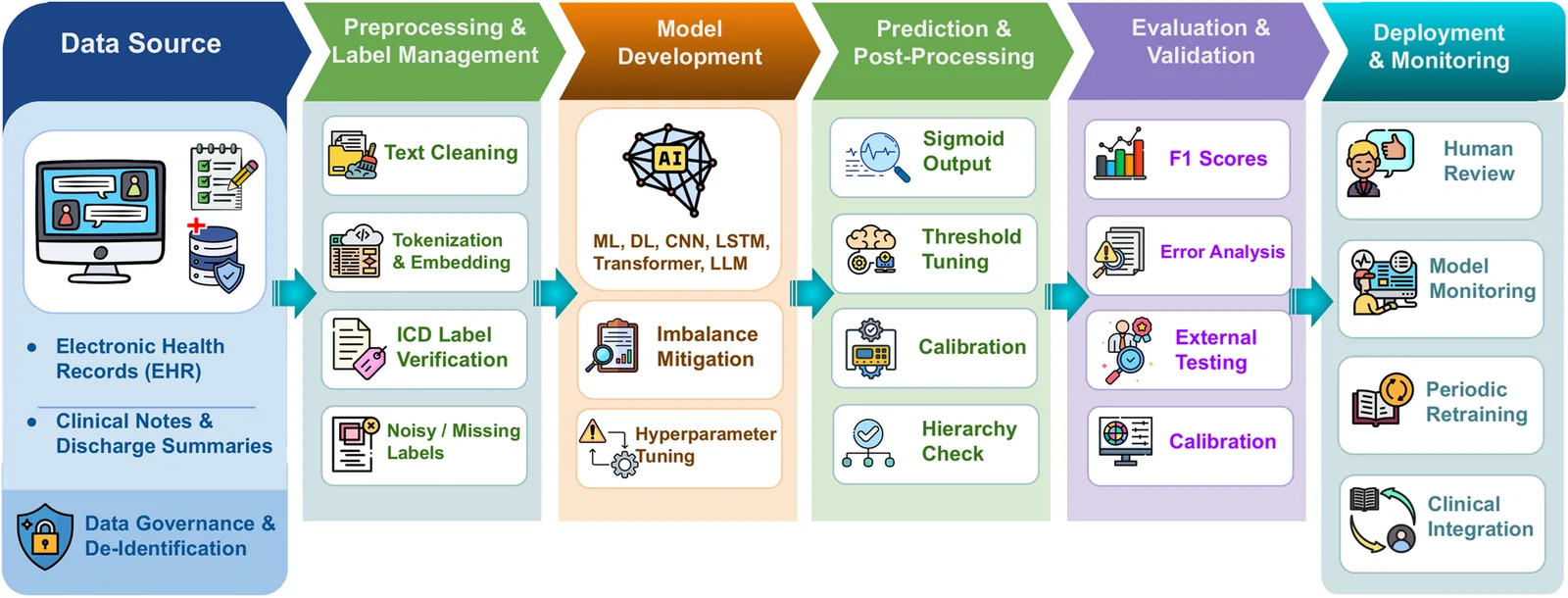
End-to-end pipeline for automatic ICD-10 coding, organized into six stages: data acquisition, preprocessing and label verification, AI model training, prediction and post-processing, evaluation and error analysis, and deployment with human oversight. Icons from Flaticon: “Chat”, “Label”, “Missing”, “Bar Graph”, “Color”, and “Satisfaction” by Magnific. “Checklist” by Smashicons, “Data Protection” by Pop Vectors”, “Shield” by monkik, “Data Cleaning” by pojok d, “Embedded” by Eucalyp, “Neural Network” by Parzival 1997, “Analysis” by syafii5758, “Risk Management” by Nuricon, “Diagnostic” by Heisenberg_jr, “Tuning” by phatplus, “Instrumentation” by gravisio, “Hierachy” by bearicons, “Survey” by Uniconlabs, “Auditoría” by Paul J., “Monitoring” by Eucalyp, “Aprendizaje Continuo” by Afian Rochman Afif and “Learning” by Uniconlabs. Licensed under Flaticon License.

In the context of automatic ICD-10 coding, multi-label text classification is one component of a broader end-to-end pipeline. As illustrated in Figure 2, we organize the literature using a six-stage pipeline, beginning with clinical data collection and ending with system use and oversight in practice (3, 4).

#### 3.2.1 Data acquisition

The process begins with the Data Source Layer, which includes information recorded in EHRs, such as discharge summaries and clinical notes. This stage also involves data governance and de-identification procedures to protect patient privacy. The quality, completeness, and standardization of the source data are important because they influence all later stages of system development and evaluation (16, 17).

#### 3.2.2 Data preprocessing and label verification

The next stage involves preparing the text and labels for analysis. This may include text cleaning, tokenization, embedding, and review of the ICD codes linked to each document. Attention to missing, noisy, or inconsistent labels is important, since label quality can directly affect AI model reliability in large-scale multi-label tasks (18).

#### 3.2.3 AI model training and hyperparameter optimization

In the AI model development stage, the system learns patterns from the clinical text using methods such as CNN, LSTM, Transformer, or PLMs. This stage may also include hyperparameter tuning and strategies to address class imbalance (2, 5, 19).

#### 3.2.4 Prediction and post-processing

After AI model inference, the predicted output must be converted into final ICD-10 code assignments. This stage may involve threshold tuning, calibration, and the application of ICD-10 hierarchical rules or constraints (20). From a clinical and operational perspective, this step determines how AI model output is translated into code decisions that may later be reviewed or used in practice.

#### 3.2.5 Evaluation and validation

The evaluation and validation stage may go beyond summary performance metrics such as micro-F1 or macro-F1, and may also include analysis of frequent and rare codes, error patterns, external validation, and calibration assessment to better reflect real-world generalizability (4, 21).

#### 3.2.6 Deployment, monitoring, and human oversight

The final stage concerns how the system is used beyond AI model development. This includes integration into hospital workflows, human-in-the-loop review, detection of AI model drift, and ongoing system improvement. This stage highlights that automatic ICD-10 coding is not only a technical prediction task, but also a clinical support process that requires governance, oversight, and long-term monitoring (6, 8, 22). The distribution of these approaches across the included studies is reported in the synthesis results.

### 3.3 Imbalance mitigation framework in ICD-10 coding

Class imbalance is a major structural challenge in automatic ICD-10 coding, especially in full-code multi-label classification with a long-tail label distribution. In this setting, a small number of codes occur very frequently, while many others appear only rarely. This pattern can bias AI model learning toward common labels and reduce performance on less frequent codes, especially in terms of F1-macro (4). Imbalance mitigation techniques reported in the literature can be applied at different stages of the learning process.

At the data level, common approaches include oversampling, undersampling, and data augmentation. Oversampling increases the number of minority-label examples, for example through methods such as SMOTE (23). Undersampling reduces the number of majority-label examples to limit AI model bias. Data augmentation generates synthetic samples close to real examples to improve coverage of the minority-label distribution. At the loss level, cost-sensitive learning assigns different weights to labels according to their frequency, so that minority labels receive greater importance during training. Other methods, such as focal loss, place more emphasis on difficult or minority examples by changing how the loss is calculated (24). At the decision level, threshold tuning and calibration can reduce the effects of imbalance. Adjusting label-specific thresholds may improve F1-macro without changing the underlying AI model representation (20, 21). At the architecture level, some methods use the hierarchical structure of ICD-10 to support prediction of rare labels. In hierarchical AI models, rare labels may benefit from information shared through parent categories. Attention mechanisms may also help the AI model focus on more informative parts of the input during representation learning (19, 25). At the training-dynamics level, curriculum learning proposes that the AI model should be trained gradually, for example from easier to more difficult examples or from coarse to fine label structures, in order to improve optimization under imbalanced conditions (26). These categories are not mutually exclusive, and individual studies often combine techniques across levels.

### 3.4 Explainability theory in clinical AI

Explainability is an important part of AI systems for automatic ICD-10 coding, especially in clinical settings where decisions may affect care, oversight, and system acceptance. In this context, explainability is relevant not only for AI model interpretation, but also for physician trust, regulatory acceptance, and readiness for real-world use in healthcare institutions (8, 27). The explainability approaches identified in the literature can be grouped as follows.

At the AI model-internal level, commonly used approaches include attention visualization and gradient-based methods, which are often considered intrinsic forms of explanation (25, 28). These methods use internal AI model signals, such as attention weights or gradients, to indicate which words or phrases may have influenced the prediction. However, whether attention weights truly reflect the actual decision process remains debated (29). Among post-hoc model-agnostic methods, SHAP estimates the contribution of each feature to the AI model output based on Shapley values from cooperative game theory (30), while LIME uses a local surrogate explanatory model to approximate AI model behavior around a specific prediction (31).

Rationale extraction identifies parts of the text that are intended to be sufficient and minimal for the prediction (32). Ontology-based explanations use knowledge representation and semantic reasoning to support interpretation, often by leveraging the hierarchical structure of ICD or related medical ontologies. Prototype-based or case-based reasoning approaches explain predictions by referring to similar past cases (33). Rule-based explanations are derived from symbolic logic and expert systems, and usually rely on interpretable if-then rules (34). Human-in-the-loop approaches extend explainability into an interactive setting by involving domain experts in AI model development, review, or evaluation (22).

## 4 Methods

This review was conducted and reported in accordance with the PRISMA 2020 guideline (9). Study selection and eligibility were structured using the PICO framework. The completed PRISMA 2020 checklist is provided in the Supplementary Material.

### 4.1 Protocol and registration

To define the procedures and criteria for the review process and study selection, the review protocol was preregistered on the Open Science Framework (OSF; https://osf.io/cegqk) on March 12, 2026. During the review process, minor protocol updates were made. These updates did not alter the main review structure.

### 4.2 Eligibility criteria

The PICO framework was used to guide the review process. In this review, the Population element was operationalized as clinical records, clinical notes, discharge summaries, or EHR datasets used for automatic ICD-10 coding. The PICO elements and additional eligibility criteria, including article type, publication year, and language, are summarized in Table 1.

**Table 1:** Inclusion and exclusion criteria based on the PICO framework.

PICO framework	Include	Exclude
Population (P) Clinical records or clinical text datasets used for automatic ICD-10 coding	ICD-10 coding systems Clinical notes Discharge summaries EHRs Inpatient or outpatient records	ICD-9, ICD-11, SNOMED, ICPC, or DRG Billing or reimbursement records without diagnostic coding
Intervention (I) AI Models or techniques used for training or fine-tuning, and other techniques in automatic ICD-10 coding systems	Rule-based ML DL Transformer LLMs Multi-label text classification	Studies not framed as multi-label text classification
Comparator (C) Other approaches used as baselines	Manual coding Alternative ML, DL, transformer, or LLM approaches used as baselines within the same study	Studies with no comparator or baseline reported
Outcome (O) Evaluation metrics used to measure system performance	F1 score (micro, macro, or weighted) Accuracy Precision and recall AUROC, AUPRC, MAP, EMR Other reported metrics	No performance measurement or evaluation reported
Other eligibility criteria		
Type of articles	Research articles Conference articles Preprints	Veterinary or animal study Opinion articles or editorials or commentaries Reviews Case reports
Publication year	Published in 2020 to 2025	Published before 2020
Article language	English Language	Non-English articles

### 4.3 Search strategy

Searches were conducted in PubMed, Scopus, Web of Science, IEEE Xplore, ACM Digital Library, arXiv, and Google Scholar. Search terms were derived from the PICO framework and grouped into three concepts: ICD-10 coding, AI-based methods, and clinical text data. The core search string was:

("ICD-10 coding" OR "clinical coding" OR "automatic coding") AND ("machine learning" OR "deep learning" OR "transformer" OR "large language model") AND ("clinical notes" OR "EHR" OR "discharge summary")

These keywords were used to retrieve and screen articles published in English between January 1, 2020 and December 31, 2025. Because database search rules and indexing structures differ, Boolean queries were adapted for each database, as shown in Supplementary Table S1. The search was conducted on March 15, 2026. An updated search was performed on July 8, 2026, to identify any additional studies indexed after the original search. Journal articles were the main focus, but relevant preprints, particularly from arXiv, were also included to capture timely contributions in this rapidly evolving field. Preprints were screened using the same inclusion and exclusion criteria as peer-reviewed articles. Preprints that had since been published in peer-reviewed venues during the screening period were replaced by their peer-reviewed counterparts. No additional registers, organizational websites, or reference-list searches were used.

### 4.4 Review processing and data extraction

Title and abstract screening and full-text screening were conducted independently by two authors (K. Tangudomkit and S. Chairat) using the Rayyan platform (https://www.rayyan.ai) for record management and screening support. Disagreements between the two authors were resolved through discussion, and unresolved conflicts were adjudicated by a third author (S. Chaichulee).

The data extraction template was designed by the authors specifically for the automatic ICD-10 coding problem. Existing AI reporting frameworks were developed for other study types, such as randomized trials of AI interventions, diagnostic accuracy studies, or live clinical evaluations, and do not align with the methodological development studies that dominate this field. The extraction template was therefore built around the structural characteristics of automatic ICD-10 coding research: a multi-label classification task with a large hierarchical label space, long-tail label distribution, heavy reliance on single-site EHR data, and limited progress toward real-world deployment. Each extraction item was chosen to capture either a methodological design choice that materially affects performance or a reporting dimension that affects credibility and reproducibility.

The extracted data items are organized as follows:

1. **Study characteristics**: author, publication year, article type, country, and study design.
2. **Dataset characteristics**: dataset name, dataset type, ICD-10 version, clinical department or data source, dataset language, dataset size, and number of labels.
3. **AI Model and methodological features**: methodological approach, AI model architecture, preprocessing and tokenization techniques, feature representation or embedding method, training strategy, and hyperparameter or fine-tuning details. When studies reported multiple AI models, baselines, or architecture, each eligible experimental evaluation was extracted separately.
4. **Imbalance handling**: label distribution and techniques used to address class imbalance, including data-level, algorithm-level, model-level, training-level, or output-level strategies. In addition, dataset size and the number of labels were used to calculate the documents-per-label ratio (D/L). This ratio was then used to classify the appropriateness of imbalance handling into five levels: extreme sparsity (<5), long-tail (5–20), acceptable (20–100), robust learning (100–1,000), and extremely strong (>1,000).
5. **Evaluation details and performance outcomes**: evaluation design, output setting, code granularity, and reported multi-label performance metrics, including F1-micro, F1-macro, F1-weighted, F1-score, precision, recall, area under the receiver operating characteristic curve (AUROC), area under the precision-recall curve (AUPRC), exact match ratio (EMR), and mean average precision (MAP) where available. All results compatible with these outcome domains were sought. For cross-study interpretation, all-code or full-code results were prioritized, whereas top-k, restricted-label, or subgroup-specific results were extracted separately and not directly combined with full-code results. F1-micro and F1-macro were prioritized because they were the most consistently reported metrics and reflected both overall and rare-label performance.
6. **Interpretability**: explainability or AI model interpretation approaches, including attention visualization, rationale extraction, rule-based methods, and ontology-based interpretation.
7. **Reproducibility and transparency**: code availability, AI model reproducibility, and ethical approval statements.
8. **Clinical integration**: evidence of clinical applicability, including whether the study remained at the research stage, involved pilot testing, or reported clinical deployment.
9. **Generalizability**: assessment of AI model generalizability, including reported limitations and potential sources of bias.
10. **Key findings**: study objectives, main findings, reported limitations, and suggested future directions.

Missing or unclear information was checked against the full text, tables, figures, Supplementary Material, or original dataset descriptions where available; otherwise, it was recorded as “not reported”. No assumptions were made beyond information explicitly reported by the authors. Microsoft Excel was used for data extraction because it provided flexibility for structuring and managing the extraction fields. Data extraction was performed by one author (K. Tangudomkit). Studies were excluded if further review showed that they met any predefined exclusion criteria. Unclear, inconsistent, or potentially ineligible items were discussed with a second author (S. Chairat) or a third author (S. Chaichulee) to ensure accuracy and consistency. No study investigators were contacted to obtain or confirm additional data, and no automation tools were used for data extraction. All retained articles were subsequently included in the quality assessment.

### 4.5 Quality assessment

Because the included studies were mostly retrospective AI model development or validation studies, complete application of existing clinical AI appraisal tools was not feasible. Many items in broader frameworks require information that is usually available only from study authors. To reduce subjective inference, this review used an adapted appraisal rubric based only on information that could be consistently extracted from the published articles. The rubric was designed to summarize methodological quality and reporting completeness in automated ICD-10 coding studies, including relevant issues such as label quality, long-tail label imbalance, and code-space complexity.

The rubric was used as a structured and transparent method to compare methodological quality and reporting completeness across studies. Each study was assessed using ten criteria, labelled Q1–Q10. Each criterion was scored from 0 to 1, and the total score was calculated by summing the ten criteria. The total score was interpreted as a descriptive completeness index.

- **Q1 Clarity of objective**: 1.0 if clearly stated; otherwise 0.0.
- **Q2 Data source transparency and label quality assurance**:
  - **Dataset reporting**: dataset size and number of labels both reported = 1.0; one reported = 0.5; none reported = 0.0.
  - **Label quality**: not reported = 0.0; partially verified or auto-assigned labels = 0.3; multi-level quality assurance = 0.6; expert-reviewed gold standard = 1.0.
  - The final score was the average of the two sub-scores.
- **Q3 AI model transparency**: reporting of five technical components, including preprocessing, architecture, hyperparameters, training procedure, and computational resources. Full reporting = 1.0; otherwise, 0.2 for each reported item.
- **Q4 Handling of label imbalance**: both label distribution and imbalance-handling method reported = 1.0; one reported = 0.5; none reported = 0.0.
- **Q5 Evaluation design and baseline comparison**:
  - **Evaluation design stated**: 1.0 if reported; otherwise 0.0.
  - **Baseline comparison or statistical rigor**: not reported = 0.0; descriptive comparison only = 0.5; statistical testing, confidence-based analysis, or advanced comparison = 1.0.
  - The final score was the average of the two components.
- **Q6 Performance metrics reporting**: appropriate multi-label metrics reported (e.g., accuracy, precision, recall, AUROC, AUPRC, MAP, EMR) = 1.0; none reported = 0.0.
- **Q7 Explainability**: reporting of an explainability method = 1.0; otherwise 0.0.
- **Q8 Reproducibility and transparency**:
  - **Code availability**: not reported = 0.0; available upon request = 0.3; partially available = 0.6; publicly available = 1.0.
  - **AI Model reproducibility**: not reproducible = 0.0; limited = 0.3; partial = 0.6; fully reproducible = 1.0.
  - **Ethics approval**: not reported = 0.0; implicit only = 0.5; explicit approval = 1.0.
  - The final score was the average of the three sub-scores.
- **Q9 Clinical integration**: research stage = 0.3; pilot testing = 0.6; clinical deployment = 1.0.
- **Q10 Generalizability, limitations, and bias**:
  - **Generalizability**: limited or single-site = 0.3; moderate or internal multi-source = 0.6; high or external or cross-domain = 1.0.
  - **Limitations and bias reported**: mentioned = 1.0; not mentioned = 0.0.
  - The final score was the average of the two components.

Quality assessment was performed by one author (K. Tangudomkit) using the standardized scoring rubric. Unclear or borderline judgments were discussed with a second author (S. Chairat) or a third author (S. Chaichulee) until consensus was reached. Microsoft Excel was used only to record and calculate scores, and no automation tools were used to assign quality scores.

### 4.6 Data synthesis approach

Data synthesis was conducted using a narrative and quantitative approach.

**Unit of analysis.** Three units of analysis were used, each clearly indicated where applicable. (i) The study level (n=24) was used for publication trends, quality assessment, and reporting practices. (ii) The experiment level (n=296, comprising 172 main AI model and 124 baseline comparator experiments) was used for AI model type and dataset-characteristic analyses, where a single study can contribute multiple experiments across different datasets or AI model variants. (iii) The metric-cell level (n=465) was used for label-selection-strategy and code-granularity analyses, where each (experiment × metric) combination is counted separately.

**Performance value extraction.** For each experiment, the highest reported value of each F-measure variant (F1-micro, F1-macro, F1-weighted) under the experiment’s evaluation setting was extracted. Where studies reported multiple variants of the same AI model under different hyperparameter or training configurations, each was treated as a separate experiment.

**Documents-per-label ratio.** The documents-per-label (D/L) ratio was computed as the size of the training set divided by the number of distinct labels in the evaluation setting of that experiment. Studies that did not separate training from evaluation splits were assigned the total dataset size as numerator.

**AI Model-type taxonomy.** AI Models were classified into six categories: ML, DL, transformer, Hybrid DL, LLM, and hybrid LLM. ML denotes traditional non-neural machine learning models using manually engineered or vectorized features. DL denotes neural models such as CNN, RNN, LSTM, or GRU that do not primarily rely on pretrained transformer backbones. Transformer denotes a standalone pretrained transformer or PLM architecture fine-tuned directly for classification. Hybrid DL denotes a transformer or PLM backbone combined with an additional task-specific module. LLM denotes generative LLMs used through zero-shot, few-shot, or instruction-based prompting. Hybrid LLM denotes generative LLMs combined with another coding component. Classification of each experiment is reported in Supplementary Table S7.

**Visualization.** Findings were summarized in tables and figures. Boxplots are used for performance comparisons; percentages are reported with their denominators stated explicitly.

The findings were then interpreted to address Research Questions RQ1–RQ11 and to propose practical guidance for future studies.

### 4.7 Reporting bias and certainty of evidence

Formal assessment of reporting bias was not performed, as no meta-analytic pooling was conducted due to the heterogeneity of datasets, AI model architectures, and evaluation protocols across included studies. Certainty of evidence was not formally assessed, as the included studies were methodological development studies rather than clinical effectiveness studies. Instead, evidence certainty was considered narratively based on study quality, consistency, directness, precision, external validation, reproducibility, and generalizability.

## 5 Extraction and synthesis results

The results are presented in three main sections: (1) the outcomes of the article selection process, (2) the results of the quality assessment of the included studies, and (3) the findings from the synthesis of the extracted data. The synthesis results are presented in sequence according to the stages of the automatic ICD-10 coding pipeline. Denominators are stated separately because some analyses were conducted at study level, experiment level, or evaluation-record level.

### 5.1 PRISMA flow reports

A structured screening process was conducted to identify studies for inclusion in this systematic review. During the identification stage, 257 records were retrieved from seven databases: Scopus (102), PubMed (21), Web of Science (24), IEEE Xplore (14), ACM (2), arXiv (67), and Google Scholar (27). After removal of 53 duplicates, 204 articles remained for title and abstract screening. Of these, 106 records were excluded, leaving 98 articles for full-text review.

Full-text assessment led to the exclusion of 74 articles for the following reasons: not related to ICD-10 (n=31), not multi-label text classification (n=21), full text not retrievable (n=7), no evaluation reported (n=7), dataset not reported (n=7), and competition report (n=1).

Inter-rater agreement between the two reviewers was fair at the title and abstract screening stage (Cohen’s κ≈0.37) and moderate at the full-text screening stage (Cohen’s κ≈0.54). The higher agreement observed during full-text screening suggests that eligibility decisions became more consistent when more detailed study information was available. All disagreements were resolved through discussion until consensus was reached.

Several studies that appeared potentially eligible during the screening were excluded after full-text assessment because they did not meet the predefined inclusion criteria or were not sufficiently comparable with the included studies. For example, Ferrão et al. (35) discussed ICD-10 coding but primarily focused on structured data and ICD-9-based experiments. Gaspar et al. (36) used ICD-10 codes to predict only bleeding outcomes. Almagro et al. (37) developed an ICD-10 prediction algorithm but reported only top-k ranking metrics, limiting comparability with most included studies. All 24 contained sufficient data for extraction, so no studies were excluded at this step.

**Figure 3:**
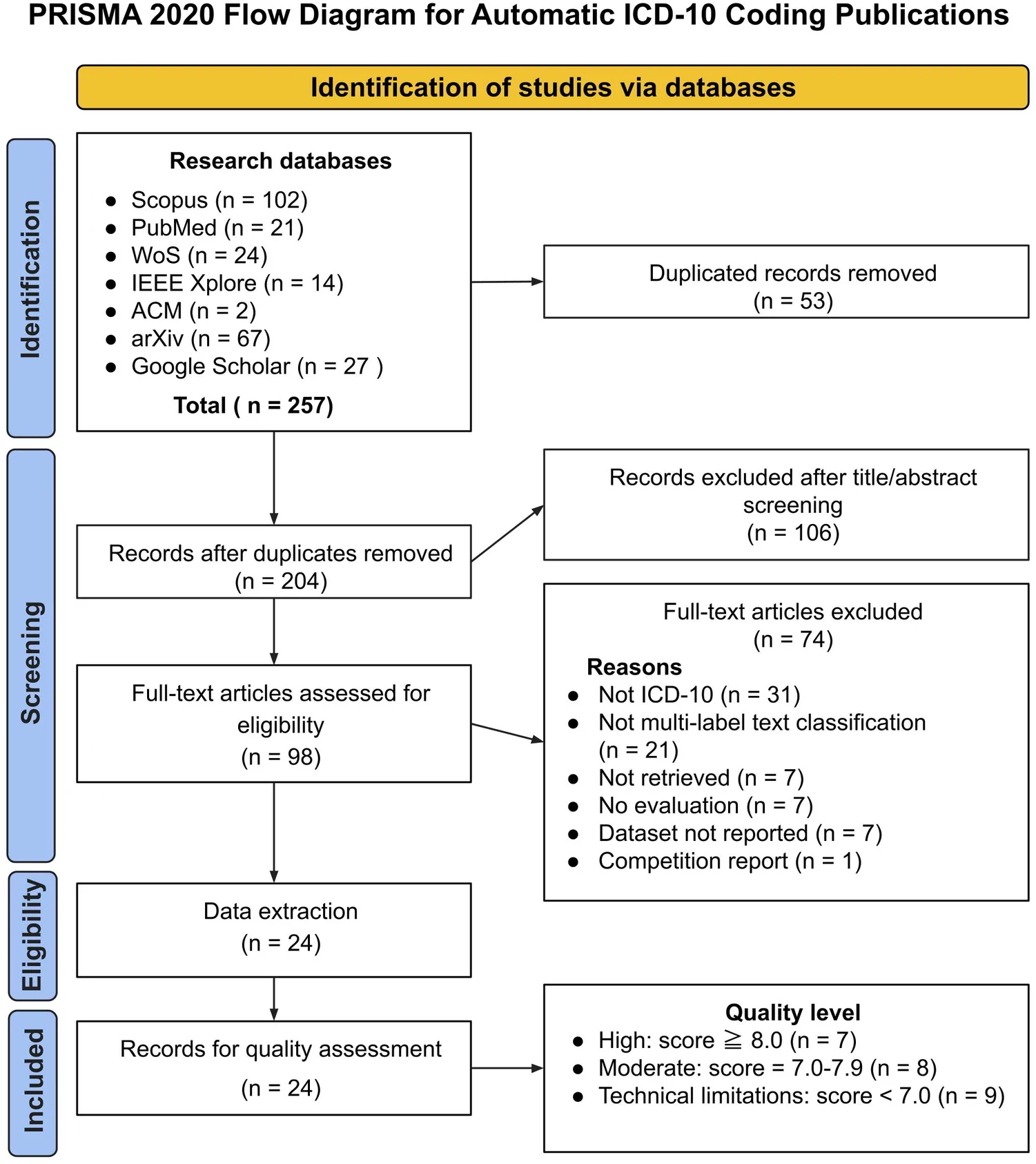
PRISMA 2020 study-selection flow diagram for automatic ICD-10 coding studies. Of 257 identified records, 204 were screened after duplicate removal, 98 underwent full-text assessment, and 24 studies were included.

After study inclusion, all 24 included articles underwent quality assessment. The article selection process is summarized in Figure 3.

### 5.2 Quality assessment

All 24 included articles underwent quality assessment. The scoring results based on the predefined criteria are presented in Table 2. The results show that all 24 studies underwent quality assessment and were categorized into three levels: high quality, with scores ≥8.0 (n=7); moderate quality, with scores of 7.0–7.9 (n=8); and limited methodological quality, with scores <7.0 (n=9). These categories were used as a descriptive summary of methodological quality and reporting completeness. The studies were then synthesized according to their relevance to the research questions. Details of the quality assessment for each study are presented in Supplementary Table S2.

**Table 2:** Quality assessment scores of the included studies based on predefined QA criteria.

Study	Q1	Q2	Q3	Q4	Q5	Q6	Q7	Q8	Q9	Q10	Total score
Wu et al. (38)	1.00	1.00	1.00	1.00	1.00	1.00	1.00	0.67	0.30	0.65	**8.62**
Motzfeldt et al. (39)	1.00	1.00	1.00	1.00	0.75	1.00	1.00	0.83	0.30	0.65	**8.53**
Gao et al. (40)	1.00	0.80	1.00	1.00	0.75	1.00	1.00	0.53	0.60	0.80	**8.48**
Falis et al. (41)	1.00	0.65	1.00	1.00	0.75	1.00	1.00	0.87	0.30	0.65	**8.22**
Nguyen et al. (42)	1.00	0.65	1.00	1.00	0.75	1.00	1.00	0.83	0.30	0.65	**8.18**
Ngo et al. (43)	1.00	0.65	1.00	1.00	1.00	1.00	1.00	0.53	0.30	0.65	**8.13**
Vassileva et al. (44)	1.00	1.00	0.80	0.50	0.75	1.00	1.00	0.70	0.30	1.00	**8.05**
Ponthongmak et al. (45)	1.00	1.00	1.00	1.00	0.75	1.00	0.00	0.87	0.30	1.00	**7.92**
Mayya et al. (46)	1.00	1.00	1.00	1.00	0.75	1.00	1.00	0.20	0.30	0.65	**7.90**
Masud et al. (47)	1.00	0.65	1.00	0.50	0.75	1.00	1.00	0.43	0.30	1.00	**7.63**
Schlegel et al. (48)	1.00	0.65	1.00	1.00	0.25	1.00	1.00	0.53	0.30	0.65	**7.38**
Mustafa et al. (49)	1.00	1.00	0.40	1.00	0.75	1.00	1.00	0.27	0.30	0.65	**7.37**
Lenc et al. (50)	1.00	1.00	1.00	1.00	0.75	1.00	0.00	0.27	0.60	0.65	**7.27**
Suvirat et al. (51)	1.00	1.00	1.00	1.00	0.75	1.00	0.00	0.43	0.30	0.65	**7.13**
Palacios et al. (52)	1.00	1.00	1.00	1.00	0.75	1.00	0.00	0.37	0.30	0.65	**7.07**
Polignano et al. (53)	1.00	1.00	0.80	1.00	0.75	1.00	0.00	0.83	0.30	0.15	**6.83**
Hauksson and Einarsson (54)	1.00	0.65	1.00	1.00	0.75	1.00	0.00	0.43	0.30	0.65	**6.78**
Bou Hatoum et al. (55)	1.00	0.50	0.80	1.00	0.75	1.00	0.00	0.27	0.60	0.80	**6.72**
Xu and Dray (56)	1.00	0.65	1.00	1.00	0.75	1.00	0.00	0.37	0.30	0.65	**6.72**
Makohon and Li (57)	1.00	1.00	0.80	1.00	0.75	1.00	0.00	0.37	0.30	0.50	**6.72**
Mustafa et al. (58)	1.00	0.65	0.60	1.00	0.75	1.00	0.00	0.70	0.30	0.65	**6.65**
Wu et al. (59)	1.00	0.65	1.00	1.00	0.75	1.00	0.00	0.37	0.30	0.50	**6.57**
Lamproudis et al. (60)	1.00	0.65	0.80	1.00	0.75	1.00	0.00	0.87	0.30	0.15	**6.52**
Dalloux et al. (61)	1.00	0.65	0.80	1.00	0.75	1.00	0.00	0.20	0.30	0.15	**5.85**

Note: Q1: Clarity of objective, Q2: Data source transparency and label quality assurance, Q3: AI model transparency, Q4: Handling of label imbalance, Q5: Evaluation design and baseline comparison, Q6: Performance metrics reporting, Q7: Explainability, Q8: Reproducibility and transparency, Q9: Clinical integration, Q10: Generalizability, limitations, and bias , And bold values indicate the total score for each study. Bold values indicate the total quality-assessment score (sum of Q1–Q10).

### 5.3 Synthesis of evidence

This section synthesizes evidence from the included studies to identify overall research trends, methodological challenges, descriptive patterns and key findings in automatic ICD-10 coding. It brings together findings on quality assessment, pipeline design, AI model performance, label-support patterns, and implementation-related reporting features.

#### 5.3.1 Overall research trends

**Figure 4:**
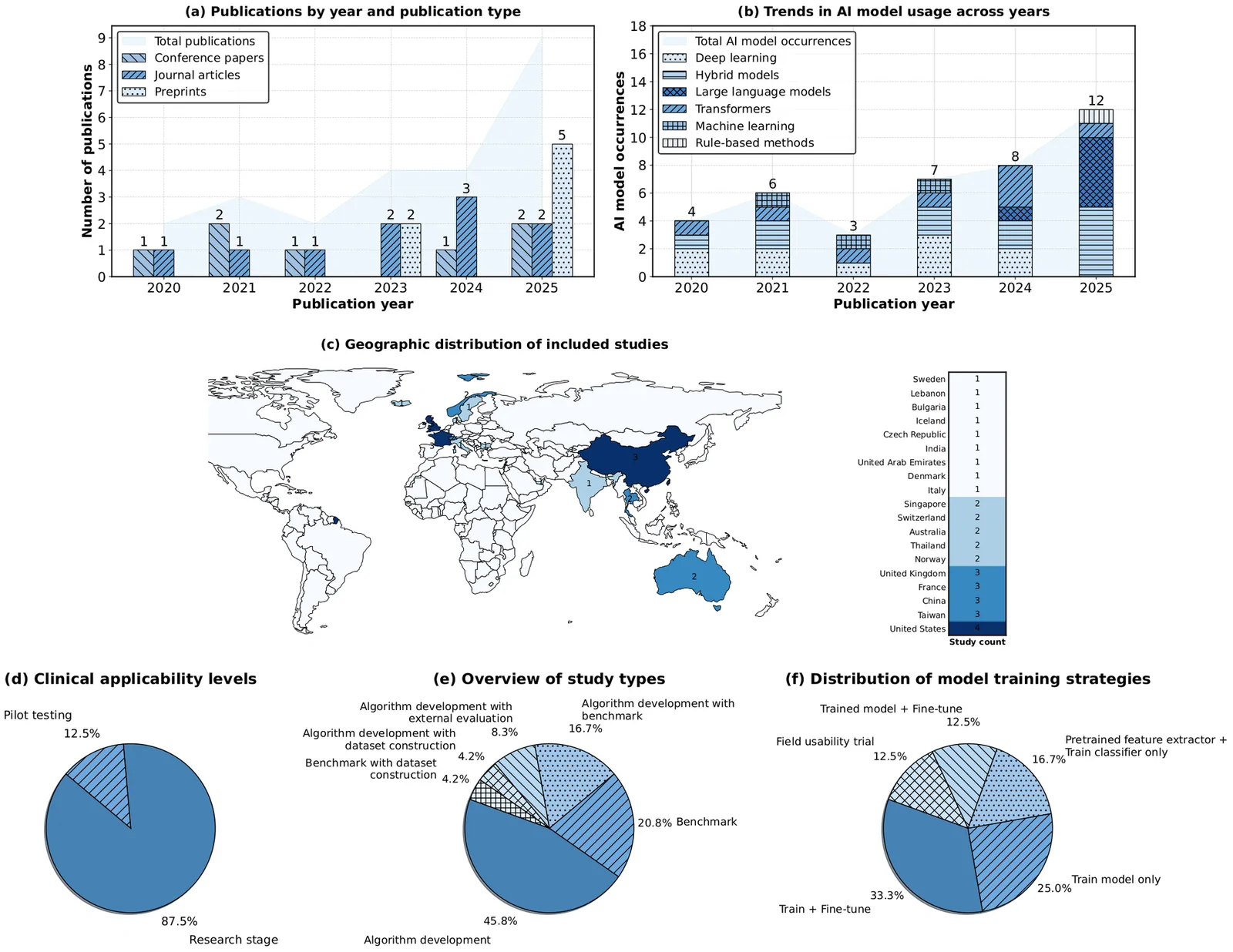
Overview of automatic ICD-10 coding research from 2020 to 2025 across the 24 included studies: **(a)** publication trends, **(b)** AI model usage, **(c)** geographic distribution, **(d)** clinical applicability, **(e)** study type, and **(f)** AI model training strategy.

An overview of publications from January 1, 2020 to December 31, 2025, based on all 24 included articles regardless of quality level, shows a generally increasing trend in research on automatic ICD-10 coding, with only a slight decline in 2022 (Figure 4a). When examined by publication type, conference papers remained relatively stable over time, while journal articles showed a steady increase and reached their peak in 2024. Preprints first appeared in 2023 and increased sharply by 2025, reflecting growing use of preprint platforms for early dissemination before formal publication.

The use of different AI model types also increased over time and reached its highest level in 2025. As shown in Figure 4b, during 2020–2021, most studies focused mainly on DL and transformer models. Hybrid models began to increase from 2020 onward. In 2024, the diversity of AI model types reached its highest level, marking the early emergence of LLMs in this field. By 2025, LLMs became the most frequently adopted AI model type, followed by hybrid models and transformers. This pattern shows a shift in AI model selection from traditional DL architectures toward LLMs and hybrid approaches.

Researchers in this field are distributed across multiple regions worldwide, as shown in Figure 4c. The United States accounts for the largest share of publications, followed by Taiwan, China, Western European countries such as France and the United Kingdom, with additional contributions from Switzerland and Norway. Asian countries, including Singapore, Thailand, and India, also contribute a substantial share. In addition, several countries, such as Australia, and a number of Northern and Eastern European countries, contributed one to two publications.

Figures 4d–f summarize clinical applicability, study type, and AI model training strategy. Most studies (87.5%) remained at the research stage without validation in real-world clinical settings, while pilot clinical testing accounted for 12.5%. Nearly half of the publications focused mainly on algorithm development. Benchmarking studies accounted for 20.8%, whereas studies combining AI model development with dataset construction or external evaluation were less common. Regarding AI model training strategy, training with fine-tuning was the most common approach (33.3%), reflecting the widespread use of pretrained AI models followed by task-specific adaptation. The use of pretrained AI models without fine-tuning was less common.

#### 5.3.2 Challenges in quality assessment

In addition to the research stage, methodological reliability and the potential for further development or clinical application also depend on label quality, explainability and trustworthiness, code availability, AI model reproducibility, transparency and ethical reporting, and generalizability across settings.

##### 5.3.2.1 Dataset and label assurance

**Figure 5:**
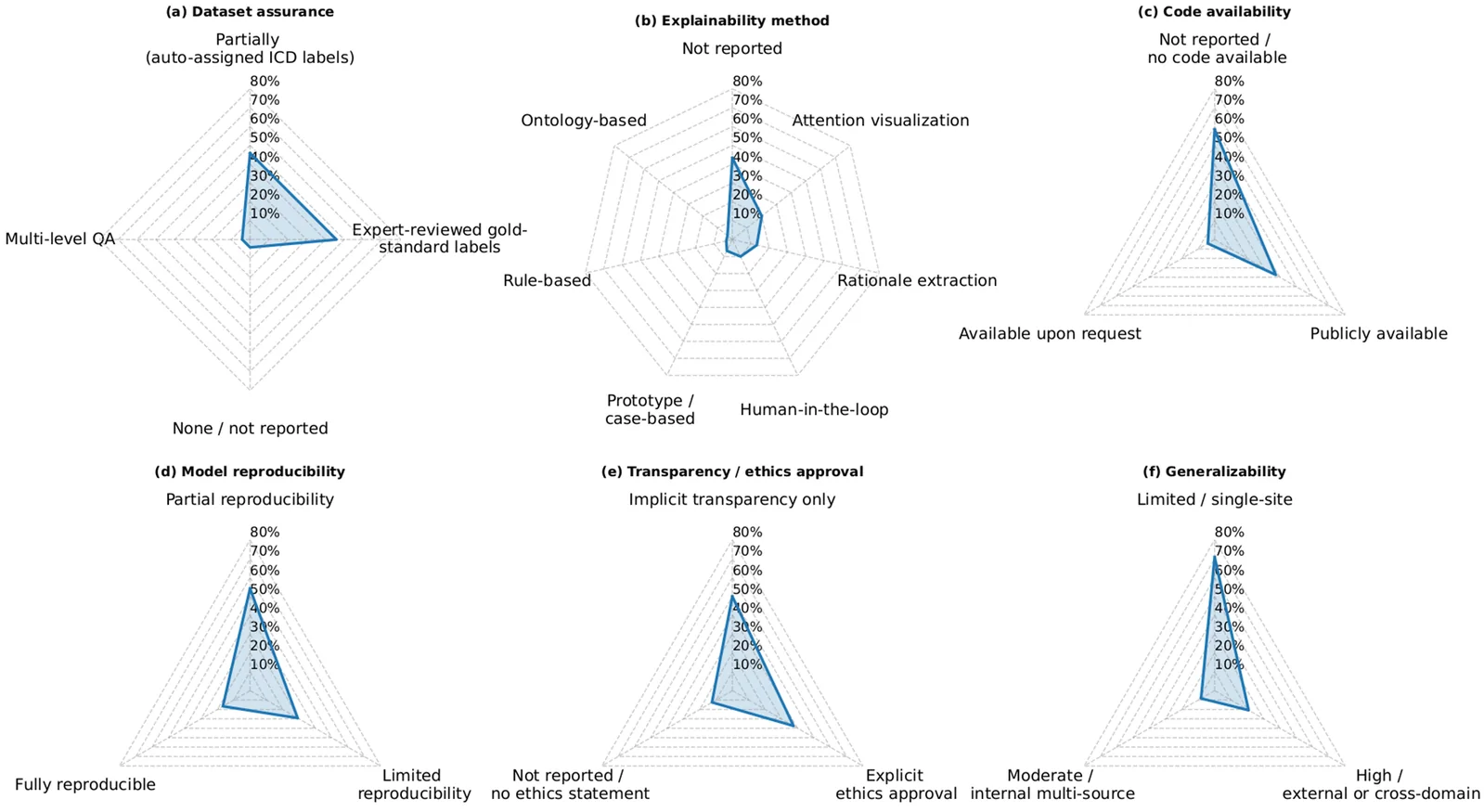
Reporting quality and methodological transparency across the 24 included studies: **(a)** label quality, **(b)** explainability method, **(c)** code availability, **(d)** AI model reproducibility, **(e)** ethics and transparency reporting, and **(f)** generalizability.

Label quality across datasets is summarized in Figure 5a. Expert-reviewed gold-standard labels and partially auto-assigned ICD labels together accounted for most included studies. In contrast, only a small proportion of studies reported multi-level quality assurance (4.2%), and a similarly small proportion did not report label quality. These results show that automatic ICD-10 coding research often relies on labels of varying quality, while systematic label verification remains limited.

##### 5.3.2.2 Explainability and trustworthiness

The explainability methods reported in automatic ICD-10 coding studies are shown in Figure 5b. The largest category was not reported (48.0%), based on data extraction results where some documents reported more than one category (denominator = 30), showing that a substantial proportion of studies did not explicitly describe any explainability method. Among the reported approaches, attention visualization and rationale extraction were the most common. Human-in-the-loop, prototype- or case-based, rule-based, and ontology-based approaches were reported less often.

##### 5.3.2.3 Code availability

Software and source code availability remained limited, as shown in Figure 5c. Many studies did not provide any source code. Publicly available code accounted for a smaller proportion, while only a small number of studies provided code upon request.

##### 5.3.2.4 AI model reproducibility

AI model reproducibility also remained limited, as illustrated in Figure 5d. The largest proportion of studies showed only partial reproducibility, usually reporting AI model architecture or evaluation procedures but not providing important details such as source code, hyperparameters, or training protocols. Another group showed only limited reproducibility, and only a small proportion could be considered fully reproducible.

##### 5.3.2.5 Transparency and ethics approval

Ethical and transparency statements were reported inconsistently, as shown in Figure 5e. Most studies provided only implicit transparency statements, while explicit ethics approval was reported in a smaller proportion. A minority of studies did not report any ethics or transparency statement.

##### 5.3.2.6 Generalizability

Generalizability remained a major limitation. As illustrated in Figure 5f, most studies used single-site datasets, which limits applicability beyond the original setting. Internal multi-source validation was less common, and only a small proportion of studies reported external or cross-domain validation.

Overall, automatic ICD-10 coding research still faces important limitations in label quality, explainability, transparency, reproducibility, and generalizability. Code availability and ethical reporting remain inconsistent, and most studies still rely on single-site datasets. Detailed information for each study is presented in Supplementary Table S2. In addition, study-level information including clarity of objectives, key findings, reported limitations, and future directions has been compiled and is presented in Supplementary Table S3.

#### 5.3.3 Steps in the pipeline

Because automatic ICD-10 coding follows a structured workflow, clinical text is first preprocessed and transformed into numerical representations using feature-based methods or PLMs. These representations are then used by AI models to generate ICD-10 predictions. The outputs may then be refined through thresholding, top-k selection, or structural constraints, and AI model performance is then evaluated using metrics such as F-measure, accuracy, precision, and recall. Based on this workflow, the included articles were mapped to the main steps of the pipeline.

##### 5.3.3.1 Dataset characteristics

Analysis of the input data sources shows that most studies (83.3%) used inpatient department (IPD) data, as summarized in Table 3. This reflects the strong focus on IPD records, which usually contain longer and more detailed clinical narratives. A smaller proportion (12.5%) used outpatient department (OPD) data, and 4.2% did not specify the care setting. This distribution shows limited use of outpatient data, where the text is often shorter and less formal.

**Table 3:** Summary of clinical care settings across included studies.

Clinical care setting	Publications	Total
IPD	Wu et al. (38), Motzfeldt et al. (39), Gao et al. (40), Falis et al. (41), Nguyen et al. (42), Ngo et al. (43), Vassileva et al. (44), Ponthongmak et al. (45), Mayya et al. (46), Mustafa et al. (49), Lenc et al. (50), Suvirat et al. (51), Palacios et al. (52), Polignano et al. (53), Xu and Dray (56), Makohon and Li (57), Mustafa et al. (58), Wu et al. (59), Lamproudis et al. (60), Dalloux et al. (61)	20 (83.3%)
OPD	Masud et al. (47), Schlegel et al. (48), Hauksson and Einarsson (54)	3 (12.5%)
Not reported	Bou Hatoum et al. (55)	1 (4.2%)

**Figure 6:**
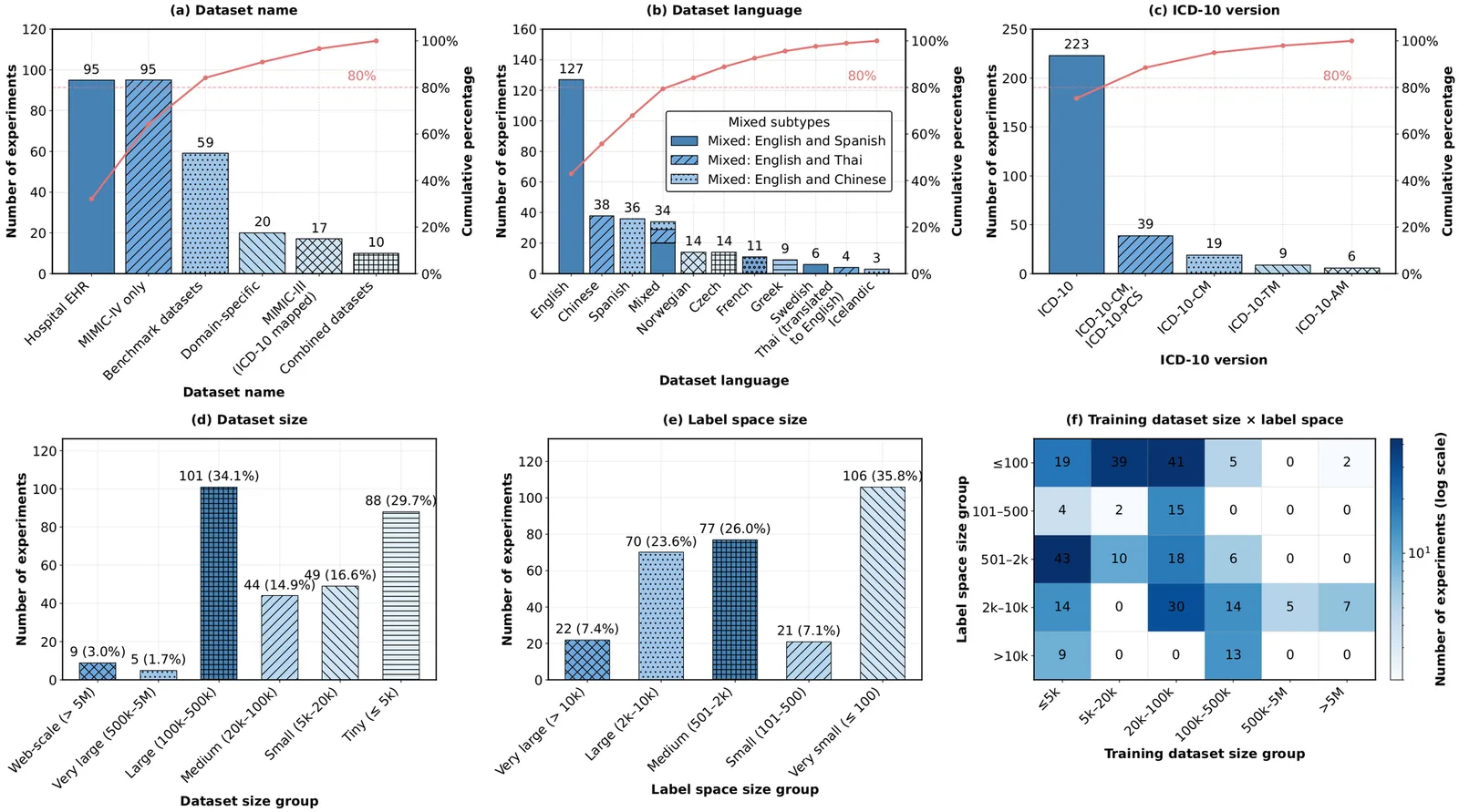
Dataset characteristics in automatic ICD-10 coding studies based on 296 experiment-level records: **(a)** data source, **(b)** dataset language, **(c)** ICD-10 version, **(d)** dataset size, **(e)** label-space size, and **(f)** joint distribution of dataset size and label-space size.

The datasets most frequently used for AI model evaluation are summarized in Figure 6, which combines main and baseline comparators experiments across 296 experiment-level records. Figure 6a shows that hospital EHRs and MIMIC datasets were the two most commonly used data sources. Benchmark datasets ranked next and were mainly used as standardized datasets for comparison. In contrast, domain-specific datasets and combined datasets from multiple sources were used much less often. Regarding dataset language, Figure 6b shows that English-language datasets were used far more often than datasets in other languages. Multilingual datasets were relatively rare and were mainly limited to a small number of languages, such as Chinese and Spanish. As shown in Figure 6c, the standard ICD-10 version was the most commonly used coding system among the included studies. ICD-10-CM with ICD-10-PCS and ICD-10-CM alone were less common, while regional versions such as ICD-10-TM and ICD-10-AM appeared only in a small proportion of studies. It should be noted that the ICD-10 category in this figure also includes translated versions of ICD-10 used in different languages, where these versions were not explicitly defined as separate clinical modification versions.

Two additional variables used to describe dataset characteristics were dataset size and label-space size, as shown in Figures 6d–f. These analyses were conducted at the experiment level rather than the study level. The largest dataset-size categories were large datasets and very small datasets, while very large and web-scale datasets were less common. In this review, web-scale datasets refer to extremely large datasets comparable in scale to data available on the web or the Internet. For label-space size, the largest single category involved very small label spaces (≤100 codes), but this represented a plurality rather than most experiments. Small-to-medium label spaces were also common, while extremely large label spaces (>10,000 codes) were less frequent. These findings show that many evaluations simplified the original complexity of ICD-10 by using restricted code sets, reduced granularity, or smaller label spaces. Detailed dataset characteristics are presented in Supplementary Table S4.

##### 5.3.3.2 Preprocessing techniques

**Figure 7:**
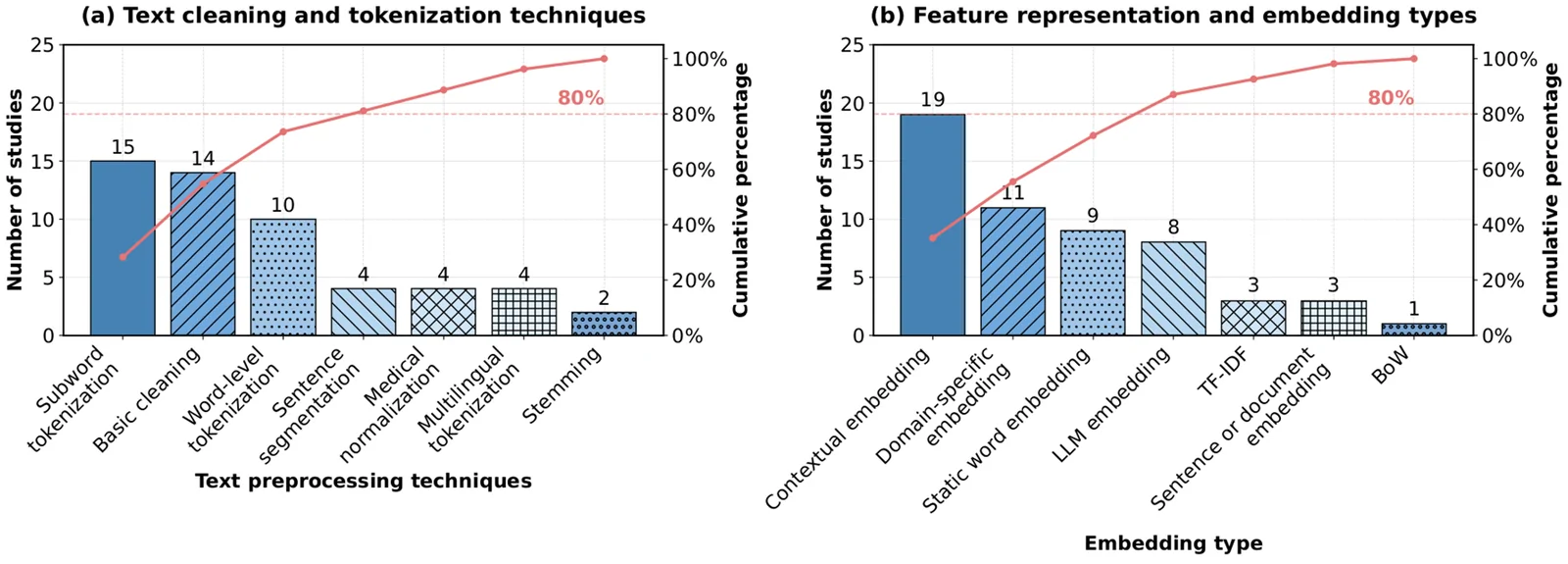
Distribution of text preprocessing techniques and embedding approaches. Bars show the number of included studies reporting each technique. Cumulative percentages use total reported occurrences as the denominator: 53 for panel **(a)** and 54 for panel **(b)**.

The preprocessing workflow before AI model training usually included text cleaning, tokenization, and feature representation, with text cleaning and tokenization often performed together. As shown in Figure 7a, subword tokenization was the most commonly used technique, followed by basic cleaning and word-level tokenization. Some studies used more than one preprocessing technique. More advanced methods appeared less often, while medical normalization and multilingual tokenization were used only in a small number of studies. Stemming was the least frequently reported technique.

Figure 7b shows the feature representation and embedding methods used in the included studies. Contextual embedding was the most frequently used approach. This was followed by static word embedding and domain-specific embedding. LLM embeddings, TF-IDF, and sentence or document embedding were used less often, while bag-of-words was uncommon. These findings show a clear preference for contextual representations over traditional vectorization methods. Publication details are presented in Supplementary Tables S5–S6.

##### 5.3.3.3 AI model training and fine-tuning

**Figure 8:**
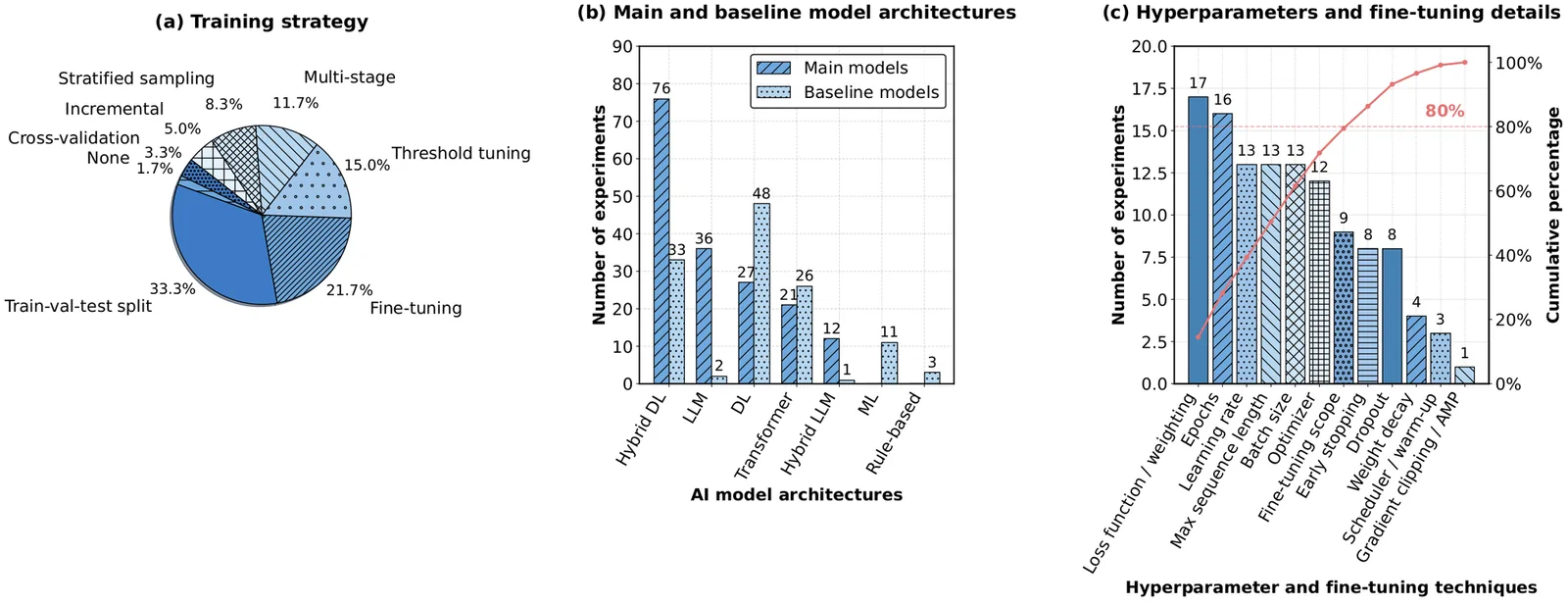
Training strategies, AI model types, and hyperparameter or fine-tuning details. **(a)** Training-strategy occurrences across 296 experiment-level records. **(b)** AI Model-type distribution across 296 experiment-level records, including 172 main AI model and 124 baseline comparator records. **(c)** Study counts for reported hyperparameter or fine-tuning details; cumulative percentages use 117 reported occurrences as the denominator.

After feature representation, the resulting vectors were used to train the AI models, with different training strategies applied, as shown in Figure 8a. The most common strategy was the train-validation-test split (33.3%), followed by fine-tuning (21.7%) and threshold tuning (15.0%). More advanced strategies, such as multi-stage training (11.7%) and stratified sampling (8.3%), were used less often. Cross-validation and incremental approaches were rarely reported.

The selection of AI model types was summarized across 296 experiment-level records, including 172 main AI model and 124 baseline comparator records, as shown in Figure 8b. Hybrid DL was the most frequently used main AI model type. In contrast, DL and transformer models were more commonly used as baseline comparators, suggesting that they had become common reference approaches in the field. Although LLMs and Hybrid LLM approaches were less frequent overall than Hybrid DL, they appeared more often as main AI models than as baselines. Detailed information on training strategies, main AI models, and baseline comparators for each study is presented in Supplementary Table S7.

The hyperparameters and fine-tuning elements most frequently reported in automatic ICD-10 coding research are shown in Figure 8c. Loss functions and weighting strategies were the most commonly reported training settings, followed by the number of epochs, maximum sequence length, learning rate, and batch size. Optimizers were also widely reported. In contrast, dropout and the scope of fine-tuning appeared less frequently, whereas early stopping, weight decay, and schedulers or warm-up strategies were reported only rarely. Overall, the results suggest that most studies relied on standard hyperparameters rather than more advanced tuning strategies. Publication details are presented in Supplementary Table S8.

##### 5.3.3.4 Evaluation approach

**Figure 9:**
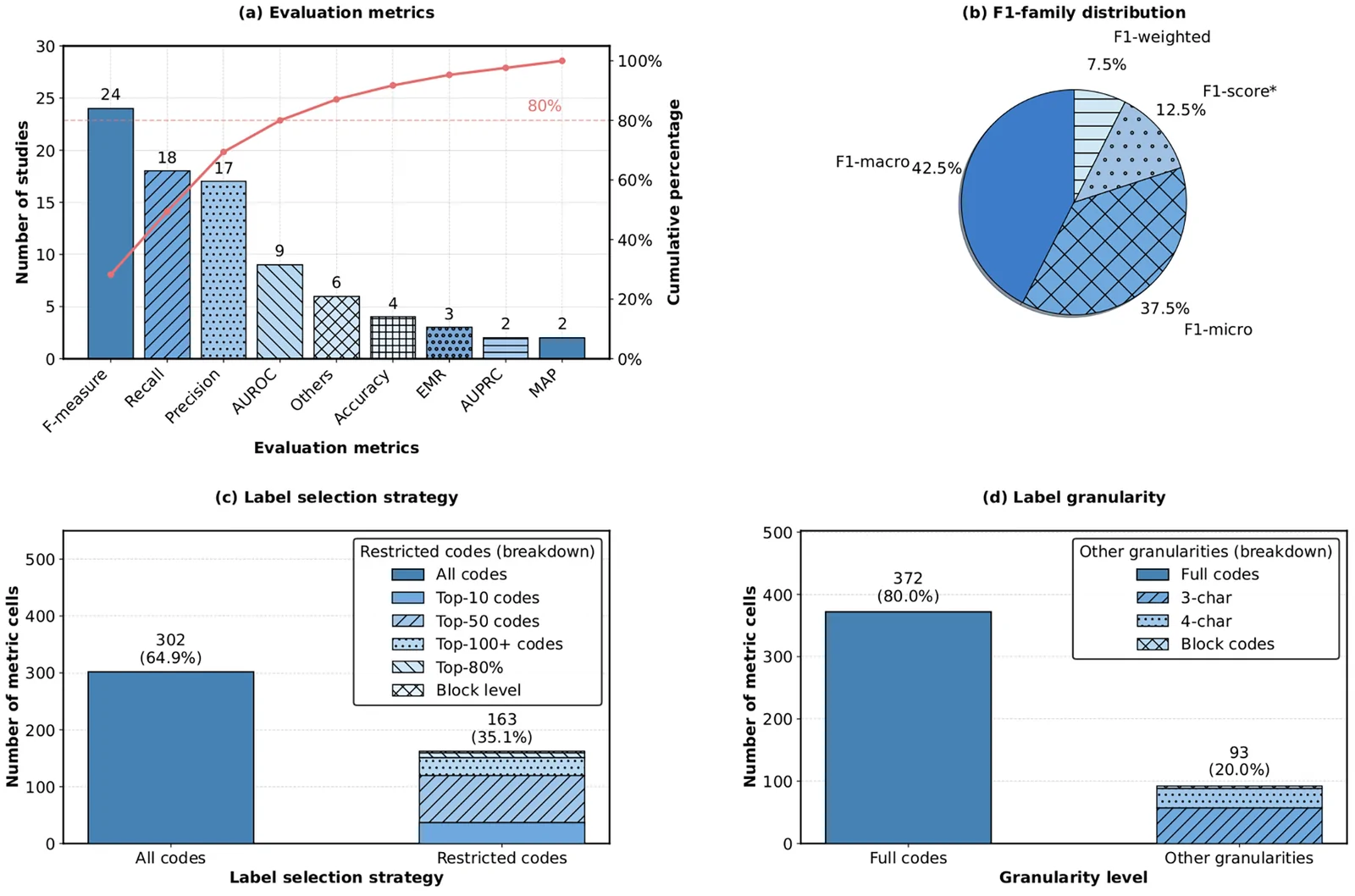
Distribution of evaluation metrics and code-format settings: **(a–b)** evaluation-metric occurrences and F1-family metric distributions, respectively, across 24 studies, with 85 reported metric instances; **(c–d)** label-selection strategies and label granularity settings, respectively, across 465 evaluation records. F1-score* indicates that the averaging method was not specified.

Several evaluation metrics were used for ICD-10 coding, as shown in Figure 9a. F-measure appeared in all 24 studies, followed by recall (18 studies) and precision (17 studies). Other metrics, such as AUROC and accuracy, were used less often, while EMR, AUPRC, MAP, and other metrics appeared only occasionally.

Figure 9b shows variation in the use of F1-based metrics. Frequent use of F1-score and F1-micro suggests an emphasis on overall predictive performance, especially on common labels. The comparable use of F1-macro indicates growing attention to label imbalance and performance across individual labels. In contrast, the limited use of F1-weighted suggests that most studies did not account for label-frequency weighting in evaluation.

The prediction formats used across evaluation records are shown in Figures 9c,d. Among 465 evaluation records, all-code settings accounted for 302 records (64.9%), while restricted-code settings accounted for 163 records (35.1%). Code restriction was applied in different forms, such as top-k prediction or block-level codes, to reduce label-space complexity. Regarding label granularity, full codes accounted for 372 records (80.0%), while reduced code granularity, such as 3-character, 4-character, or block-level codes, accounted for 93 records (20.0%). Detailed information on evaluation metrics and code formats is presented in Supplementary Tables S9–S10.

#### 5.3.4 AI model performance comparison

**Figure 10:**
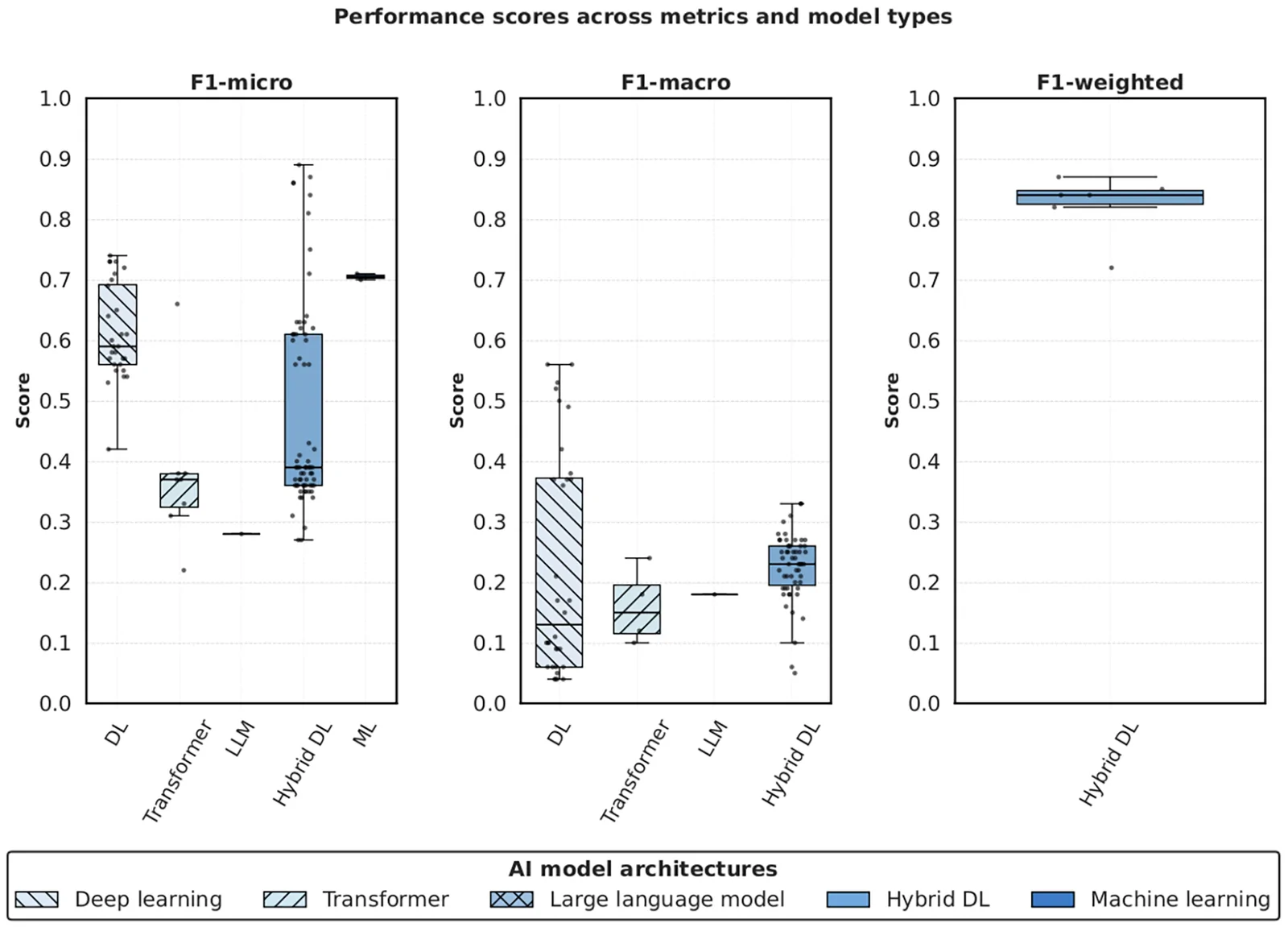
Descriptive performance comparison across F1-score variants and AI model types, based on 210 all-code/full-code experiments with more than 1,000 labels.

AI model performance comparisons were restricted to all-code and full-code experiments with more than 1,000 labels, resulting in 210 experiments. The performance outcomes are presented in Figure 10. Because some studies contributed multiple experiments, the results were interpreted descriptively and were not treated as statistically independent.

When F1-micro, F1-macro, and F1-weighted were examined, the effect of class imbalance became clearer. For F1-micro, ML, DL, and transformer models showed relatively high median values. Hybrid DL showed greater variance. In contrast, all AI model types showed a clear drop in F1-macro, indicating continued difficulty in handling rare labels. As noted by Edin et al. (62), F1-macro comparisons should be interpreted cautiously because differences in experimental settings, label filtering, threshold tuning, and metric calculation may limit direct comparability across studies. DL showed particularly high dispersion. LLMs showed relatively low and narrowly distributed F1-macro scores in the included settings, but these results should be interpreted cautiously because LLM experiments were heterogeneous and often involved zero-shot or few-shot evaluation. For F1-weighted, results were reported only for Hybrid DL experiments.

These results summarize the median and maximum reported performance across AI model types. Because F1 values are continuous and were extracted from heterogeneous experiments, median values were used only as descriptive summaries of repeated reported values. Hybrid DL showed relatively high median values across several metrics and a high upper performance range in selected experiments. DL showed lower median values, while its maximum scores reached F1-micro = 0.74 and F1-macro = 0.56. The gap between median and maximum values suggests that strong performance was achieved in some studies but was not consistent across the group. Transformer models showed similar median and maximum values in some metrics, suggesting less variation in this subset. LLMs showed relatively low and closely aligned median and maximum values in the included settings. No exceptionally high performance was observed in this group under the included all-code and full-code settings.

#### 5.3.5 Handling of imbalance and performance

**Figure 11:**
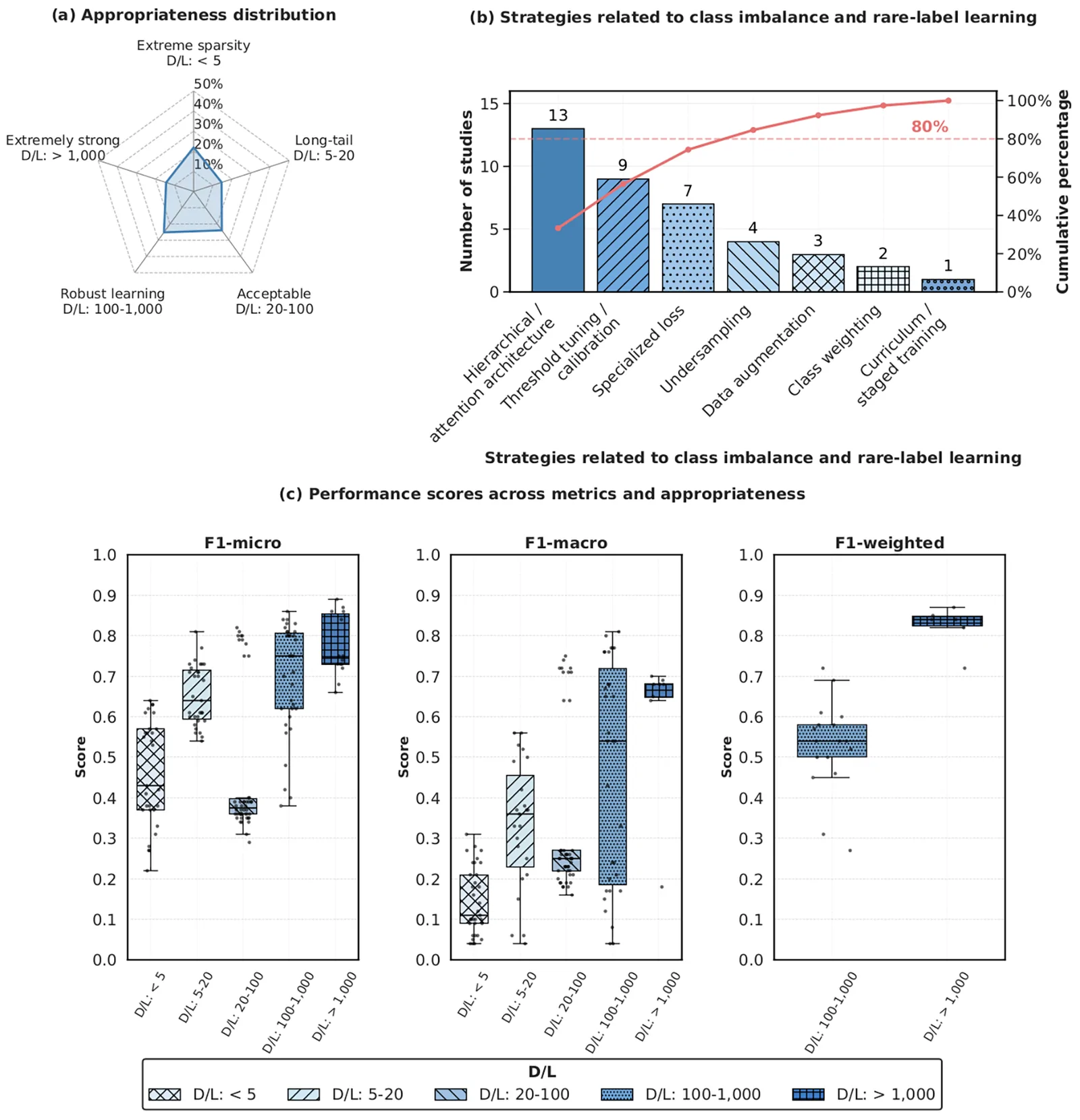
Label support, imbalance handling, and F1-score patterns in automatic ICD-10 coding studies: **(a)** D/L-based label-support levels across 372 full-code evaluation records; **(b)** imbalance-handling techniques reported across 24 studies, with cumulative percentages based on 39 reported strategy occurrences; **(c)** descriptive performance comparison across F1-score variants and D/L, based on 210 all-code/full-code experiments. Boxplots show the median and interquartile range (IQR). Whiskers extend to the most extreme observations within 1.5 × IQR, and points beyond the whiskers are displayed as outliers.

From the comparative analysis of different AI model types, class imbalance remained a major factor affecting AI model performance. Figure 11a presents AI model performance in relation to label-support levels based on documents per label. The figure includes full-code experiments across 372 evaluation records. Many studies reduced imbalance severity to some extent during AI model development or evaluation.

The strategies related to class imbalance and rare-label learning are shown in Figure 11b. Because some studies reported more than one strategy, percentages were calculated from 39 reported strategy instances. Architecture-based approaches, including hierarchical or attention-based models were reported in 13 studies (33.3%), threshold tuning or calibration in 9 studies (23.0%), and specialized loss functions in 7 studies (17.9%). Other techniques, including undersampling, class weighting, data augmentation, and curriculum or staged training, were less common. Detailed information on D/L groups and corresponding strategies is presented in Supplementary Table S11.

As shown in Figure 11c, performance varied across D/L groups and F1-score variants. Higher D/L was generally associated with better reported performance in some settings, especially for F1-micro and F1-weighted. However, the pattern was not uniform across all metrics. F1-macro remained more variable in the lower D/L groups, reflecting the difficulty of modeling rare labels when fewer documents were available per label. The 20–100 D/L group did not show higher F1-micro or F1-macro scores than the lower D/L groups. This may be because experiments in this group mainly used simpler MLP-based models, while other D/L groups included stronger transformer-based or more recent DL models. Therefore, the observed performance may reflect both D/L and AI model architecture, not D/L alone.

These results summarize the median and maximum reported performance across D/L groups. The higher D/L groups, especially the 100–1,000 and >1,000 D/L groups, showed higher reported F1-micro and F1-weighted values in some settings, while the <5 D/L group generally showed lower reported values, especially for F1-macro. The 5–20 and 20–100 D/L groups showed mixed patterns, with some high maximum values but less consistent performance across metrics. Overall, F1-macro remained lower and more variable than F1-micro or F1-weighted, indicating that rare-label prediction remained difficult even when overall performance appeared satisfactory. These findings suggest that AI model performance may improve as D/L increases, especially for micro- and weighted-level metrics. However, macro-level performance remains more sensitive to low D/L settings.

Detailed information for each study regarding the techniques applied throughout the automatic ICD-10 coding pipeline, together with their corresponding performance comparisons, is presented in Supplementary Tables S12–S15. These findings are further summarized in Supplementary Tables S16–S22. Across the included studies, Hybrid DL achieved high reported values in selected settings. However, these comparisons should be interpreted descriptively because experiments differed in dataset source, label space, code granularity, and evaluation setting.

#### 5.3.6 Performance-based guidelines for selecting documents per label and AI model types

**Figure 12:**
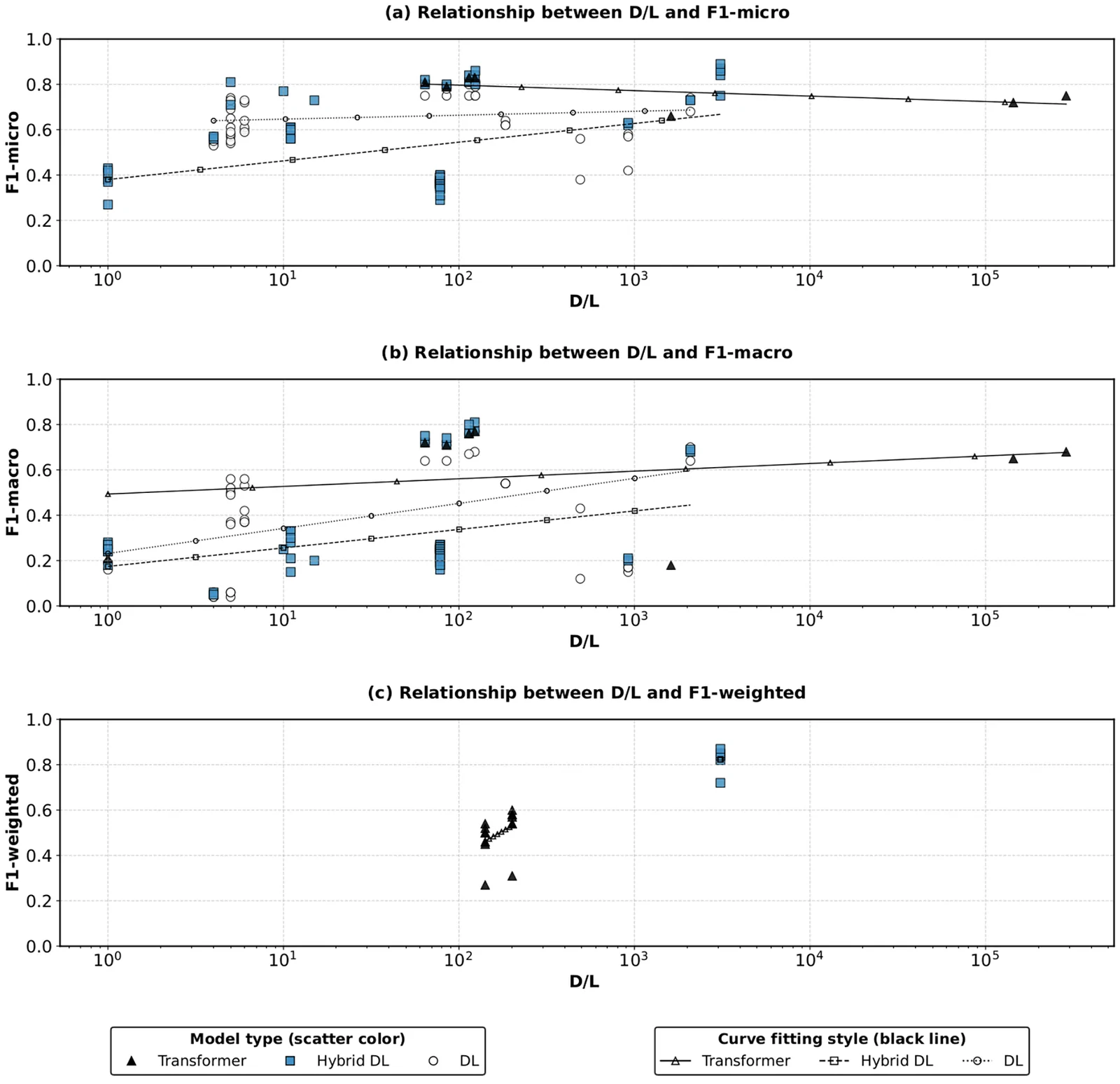
Association between D/L and F1-based performance by AI model architecture: **(a)** F1-micro, **(b)** F1-macro, and **(c)** F1-weighted.

Two variables that may influence AI model performance are the number of documents per label (D/L) and the type of AI model. Their relationship with performance is shown in Figure 12.

Figure 12a compares D/L with F1-micro. Transformer models showed relatively high F1-micro across several D/L ranges and appeared more stable than other model types in the medium-to-high D/L range ($10^{2}\text{--}10^{4}$). The trend line was relatively flat, suggesting that F1-micro did not increase linearly with D/L in this subset. Hybrid DL showed a clearer increase in F1-micro as D/L increased. In lower D/L settings (D/L < 10), its reported F1-micro was around 0.45, while values increased to approximately 0.65–0.70 when D/L reached about $10^{3}$ or higher. DL showed lower F1-micro in low-D/L settings and greater variation across the range, with some evidence of improvement followed by a plateau or decline at higher D/L values.

Figure 12b compares D/L with F1-macro. F1-macro remained lower and more variable than F1-micro across AI model types, indicating persistent difficulty in rare-label prediction. Transformer models showed a gradual increase from approximately 0.30 in lower D/L settings to around 0.65–0.70 in higher D/L settings, although the curve appeared to plateau or decline slightly at the highest D/L values. Hybrid DL began with lower F1-macro values in lower D/L settings, around 0.20, and increased as D/L grew. DL showed low F1-macro in low-D/L settings, approximately 0.05–0.10, but some experiments improved to around 0.55 when D/L reached approximately 10${}^{2}$. However, this pattern was not consistent across the full range.

Figure 12c compares D/L with F1-weighted performance. F1-weighted results were available only for Transformer and Hybrid DL experiments, and no fitted trend line was shown for DL. Transformer models appeared mainly in medium D/L settings, approximately 100–200 documents per label, with F1-weighted scores ranging from about 0.30 to 0.60. Hybrid DL was observed in higher D/L settings, around 2,000–3,000 documents per label, and showed higher F1-weighted scores, approximately 0.70–0.90. However, this pattern should be interpreted cautiously as F1-weighted data were limited and reported only for a small number of AI model categories.

Across all three panels, several descriptive patterns were observed. Low-D/L settings were associated with lower and more variable performance, especially for F1-macro. Transformer models appeared relatively stable in medium-to-high D/L ranges, while Hybrid DL showed stronger dependence on larger D/L values in this extracted dataset. DL showed improvement in some low-to-moderate D/L settings but also showed greater variability. Increasing D/L to very high levels does not necessarily lead to proportional gains for all AI model types.

### 5.4 Addressing the research questions

Based on all research questions, the answers can be summarized as follows:

- **RQ1**: What is the overall publication landscape from 2020 to 2025, and which types of AI models have been investigated? **Answer**: Research on automatic ICD-10 coding increased during the review period. The included studies investigated ML, DL, transformer, Hybrid DL, LLM, and Hybrid LLM approaches. ML and rule-based approaches were used mainly as baselines, while Hybrid DL, transformer, and LLM-based approaches became more prominent in recent years.
- **RQ2**: At what stage are most studies conducted, how are experiments designed, and what testing settings are commonly used for AI model evaluation? **Answer**: Most studies remained at the research stage, with only a small proportion reaching pilot testing. Most studies focused mainly on algorithm development or benchmarking. Common training strategies included train-validation-test splitting, fine-tuning, threshold tuning, and multi-stage model development.
- **RQ3**: What is the overall methodological quality of the included studies across key criteria, including dataset assurance, explainability, code availability, reproducibility, transparency, and generalizability? **Answer**: Common limitations included incomplete explainability reporting, limited code availability, partial reproducibility, inconsistent ethics reporting, and limited external validation.
- **RQ4**: What are the main characteristics of the datasets and code-set sizes used in the included studies? **Answer**: Most studies used inpatient EHR data, often in English. Hospital EHR and MIMIC datasets were the most common data sources. Dataset sizes are commonly either large (100–500 k) or tiny (≤5k). The most frequently used label spaces are very small (≤100 codes), followed by medium (501–2 k) and large (2–10 k).
- **RQ5**: Which text preprocessing and feature representation techniques are most commonly used? **Answer**: Most studies used preprocessing methods aligned with modern PLMs. Subword tokenization, basic cleaning, and word-level tokenization were commonly reported. For feature representation, contextual embeddings were the most frequent, followed by static word embeddings and domain-specific embeddings. Traditional vectorization methods such as TF-IDF and bag-of-words were less common.
- **RQ6**: Which AI models are most often selected as the main AI models, and which are most often used as baseline comparators? **Answer**: Across 296 experiment-level records, Hybrid DL was the most frequently used main AI model type. DL and transformer models were commonly used as baselines, together with ML and rule-based approaches. LLM and Hybrid LLM approaches appeared more often as main AI models than as baselines, especially in recent studies.
- **RQ7**: Which hyperparameters and fine-tuning details are most commonly reported? **Answer**: Most studies rely on standard hyperparameter tuning strategies. Loss functions and weighting strategies were the most commonly reported training settings, followed by epochs, maximum sequence length, learning rate, batch size, and optimizer. More detailed tuning components were reported less consistently.
- **RQ8**: Which evaluation metrics and evaluation protocols are most commonly used? **Answer**: F1-family metrics were the most commonly used evaluation metrics, followed by recall and precision. F1-micro was frequently used to summarize overall performance, while F1-macro was important for evaluating rare-label performance.
- **RQ9**: How are F-measure metrics reported across different AI model types and evaluation settings? **Answer**: F1-micro was generally higher than F1-macro across AI model types, showing the continuing challenge of rare-label prediction. Hybrid DL achieved high reported values in selected settings.
- **RQ10**: What label-support patterns and imbalance-handling strategies are reported, and how are these associated with reported performance? **Answer**: Label-support levels varied across studies and experiments. Hierarchical or attention-based architectures, threshold tuning or calibration, and specialized loss functions were the most frequently reported imbalance-handling strategies. Higher label support was associated with better reported performance in some settings, but the pattern was not uniform across metrics or AI model types. F1-macro remained more sensitive to sparse and long-tail label distributions than F1-micro or F1-weighted.
- **RQ11**: How does the relationship between documents per label (D/L) and AI model type influence performance? **Answer**: The relationship between D/L and AI model type has a clear effect on performance and varies by data regimes. DL appears to be more suitable for low D/L settings, where performance improves quickly at first but then plateaus or declines. Transformers perform more consistently in medium-to-high D/L settings, with steady improvement and lower variance. Hybrid DL depends more strongly on large data volumes, with performance improving substantially at high D/L levels and approaching transformer performance when sufficient data are available.

### 5.5 Architectural trade-offs and performance implications

The synthesis suggests that reported performance was influenced by both AI model architecture and the level of label support, particularly the availability of sufficient training examples across ICD-10 codes. Across the included studies, Hybrid DL models showed the strongest overall performance, especially in challenging settings such as full-code prediction and datasets with substantial class imbalance. These models appeared to be well suited to structured multi-label ICD-10 coding, where both representation learning and label-level modeling are important.

Standalone transformer models also demonstrated competitive and relatively consistent performance, particularly when task-specific fine-tuning was applied. Non-Hybrid DL models remained important both as primary AI models and as baselines, although their performance varied more substantially across studies.

In contrast, standalone LLMs showed weaker evidence in full multi-label ICD-10 classification settings. Their F1-based results were generally lower than those of structured AI coding approaches, suggesting that they were not yet competitive for broad, structured ICD-10 coding tasks. This result is directionally consistent with Soroush et al. (63) and Wu et al. (64), who showed that LLMs achieved limited performance in medical code querying based on exact-match accuracy. Although the evaluation metrics differ, both findings indicate that current standalone LLMs remain insufficient for reliable ICD-10 coding. However, LLM-based approaches were still emerging in the included evidence base and were often evaluated under zero-shot, few-shot, or retrieval-assisted settings, including retrieval-augmented systems (65), rather than full-code prediction from real-world clinical text.

Overall, Hybrid DL and fine-tuned transformer-based approaches currently appear to provide stronger and more reliable performance for structured multi-label ICD-10 coding than standalone LLMs.

## 6 Discussion

The findings suggest that AI model selection for automatic ICD-10 coding should be guided not only by architecture, but also by the structural characteristics of the data, especially label density and class imbalance. The analysis of the relationship between the D/L ratio and performance metrics suggests that label density is associated with performance scalability and can be grouped into different regimes with different performance patterns.

In cases of extreme sparsity (D/L<10), F1-macro declines sharply, and performance differences between AI model architectures become less clear. In this setting, the main limitation appears to be the small number of samples available per label rather than AI model capacity itself. Increasing architectural complexity alone may therefore not provide substantial benefit. Instead, strategies that directly address imbalance may be more useful, such as hierarchical modeling based on ICD-10 structure, imbalance-aware loss functions, threshold tuning, and targeted data augmentation for rare labels.

When label density increases to a moderate level (D/L≈20-100), performance tends to improve in both F1-micro and F1-macro. This range appears to be a transition zone in which more advanced architectures may become more worthwhile. Transformer and hybrid models appear to show stronger scalability than conventional DL models, especially for learning contextual information and inter-label relationships. In this regime, macro-level metrics may be more important because they better reflect the AI model’s ability to handle long-tail label distributions.

In high-density settings (D/L>100-1000), additional data appear to provide diminishing returns. F1-micro tends to plateau, while F1-macro increases only slightly. At this stage, performance may be constrained less by data quantity and more by structural modeling capacity and learning strategy. Efforts may therefore be better directed toward structured output modeling, inter-label dependency learning, and calibration rather than simply increasing dataset size.

AI model selection may also be aligned with the evaluation objective. If the aim is to maximize F1-micro, which gives greater weight to frequent labels, conventional DL models may still be adequate in moderate-to-high data settings. However, for full-code classification with greater sensitivity to rare labels, F1-macro may be a more informative indicator of robustness. In such settings, transformer or hybrid models combined with systematic imbalance mitigation strategies may be more appropriate. In summary, label density appears to be an important condition for automatic ICD-10 coding performance, but it is not sufficient on its own. Stable and scalable performance depends on the alignment of data structure, evaluation goals, and AI model design.

Overall, much of the existing research still emphasizes performance metrics, while transparency, reliability, and real-world clinical readiness have progressed more slowly. Inconsistent data quality and reporting practices make cross-study comparison difficult and limit reproducibility and stability assessment in new settings. These gaps suggest that, despite clear technical progress, more complete reporting of performance, transparency, ethics, and clinical readiness is still needed. Without further progress in these areas, real-world implementation of AI-based ICD-10 coding systems may continue to face barriers related to trust and acceptance at both institutional and policy levels.

## 7 Limitations and future research directions

Several limitations of this review should also be noted. First, the included experiments are heterogeneous in dataset, language, label-set size, code granularity, task type, and ICD-10 variants or national modifications, so cross-study performance comparisons remain descriptive rather than meta-analytic. Differences in ICD-10 versions may also affect code granularity and structure, and inconsistent reporting of these versions may obscure the relationship among AI model architecture, label space, and observed performance. Second, multiple experiments from the same study were treated as separate observations, which may give more weight to studies that reported many AI model variants. Third, median values were used to summarize continuous F1 scores, which may be unstable when only a small number of experiments are available per group. Fourth, automated ICD-10 coding challenges may also reflect the hierarchical structure of ICD-10, which is less able to capture the complexity of clinical documentation. Although AI-based tools can improve coding efficiency and consistency, they cannot fully resolve this structural mismatch. These limitations should be taken into account when interpreting the patterns reported above.

Taken together, future work may move in three directions. First, primary studies should report enough detail on preprocessing, hyperparameters, training procedure, code availability, and ethics approval to support reproducibility and external validation, including multi-site evaluation. Second, evaluation should move beyond aggregate F1-family metrics toward stratified analysis by label frequency, code granularity, and task type, in order to better reflect rare-label and long-tail behavior. Third, system-level studies should assess implementation readiness in real clinical workflows. Although AI-based coding models show promising benchmark performance, evidence remains insufficient for real-world deployment. Future work should assess not only predictive performance, but also clinical validity, workflow integration, error impact, auditability, and human oversight. Fourth, research should consider how the transition from ICD-10 to ICD-11 may affect automated coding tasks. Although ICD-11 offers a more flexible ontology-based structure, ICD-10 remains widely used, making its improvement an important near-term priority (66). These directions may help bridge the gap between reported performance and real-world deployment of automatic ICD-10 coding systems.

## 8 Conclusion

This systematic review synthesized 24 articles on AI-based automatic ICD-10 coding published between 2020 and 2025, covering 296 AI experiment-level records. Overall, the evidence shows clear technical progress, particularly through DL, transformer-based models, hybrid architectures, and more recent LLM-based approaches.

Most studies used inpatient EHR data, with English-language datasets, hospital EHRs, and the MIMIC family being the most common data sources. Contextual embeddings and PLMs were used more often than conventional vector-based ML or DL methods. However, many evaluations were conducted using restricted or relatively small label spaces, suggesting that the full complexity of ICD-10 coding is still often simplified in experimental settings.

Evaluation relied mainly on F1-family metrics. Across studies, F1-micro was generally higher than F1-macro, indicating that frequent labels were predicted more reliably than rare labels. Hybrid DL reported strong performance in several settings, while transformer and LLM results varied according to dataset, task type, label space, and evaluation design. Rare-label prediction remained a major challenge, particularly in sparse and long-tail settings. These findings suggest that AI model architecture alone is not sufficient. Dataset size, label support, label quality, code granularity, and evaluation setting all need to be considered when developing or comparing automatic ICD-10 coding systems.

Despite technical progress, many studies remained at the research stage, relied on single-site datasets, and reported limited external validation or clinical deployment. Reporting of explainability, reproducibility, code availability, ethics approval, and clinical workflow integration was also inconsistent. These gaps limit generalizability and make it difficult to assess readiness for real-world clinical use.

Future research should move beyond offline performance comparison toward more reproducible and clinically grounded evaluation. This includes clearer reporting of label quality, documents per label, AI model implementation details, source code, external validation, and human-in-the-loop design. Multi-site datasets, prospective evaluation, and consistent rare-label assessment will be important for translating automatic ICD-10 coding from experimental systems into reliable clinical tools.

## Data Availability

The original contributions presented in the study are included in the article/Supplementary Material, further inquiries can be directed to the corresponding author.
