# Supplementary material for: Artificial intelligence for automated ICD-10 coding: a systematic review of multi-label text classification in clinical narratives
Source: Front Digit Health. 2026 Aug 13;8:1905379. doi: 10.3389/fdgth.2026.1905379 (PMC13520067; doi:10.3389/fdgth.2026.1905379)
Supplement: Supplementary file 1 [file Datasheet1.pdf]

Supplementary Material

Table S1. Search strategy and keyword combinations used across multiple databases.

Database	Keyword search
PubMed	("ICD-10 coding"[Title/Abstract] OR "automatic ICD-10"[Title/Abstract] OR "clinical coding"[Title/Abstract] OR "medical coding"[Title/Abstract]) AND ("rule-based"[Title/Abstract] OR "machine learning"[Title/Abstract] OR "neural network"[Title/Abstract] OR "deep learning"[Title/Abstract] OR "transformer"[Title/Abstract] OR "large language model"[Title/Abstract] OR "LLM"[Title/Abstract]) AND ("clinical notes"[Title/Abstract] OR "electronic health record"[Title/Abstract] OR "EHR"[Title/Abstract] OR "discharge summary"[Title/Abstract] OR "medical record"[Title/Abstract]) AND ("2020/01/01"[Date - Publication] : "2025/12/31"[Date - Publication])
Scopus	TITLE-ABS-KEY ("ICD-10 coding" OR "automatic ICD-10" OR "clinical coding" OR "medical coding") AND TITLE-ABS-KEY ("rule-based" OR "machine learning" OR "neural network" OR "deep learning" OR "transformer" OR "large language model" OR "LLM") AND TITLE-ABS-KEY ("clinical notes" OR "electronic health record" OR "EHR" OR "discharge summary" OR "medical record") AND (PUBYEAR > 2020 AND PUBYEAR < 2025) AND (LIMIT-TO (LANGUAGE, "English"))
Web of Science	TS=("ICD-10 coding" OR "automatic ICD-10" OR "clinical coding" OR "medical coding") AND TS=("rule-based" OR "machine learning" OR "neural network" OR "deep learning" OR "transformer" OR "large language model" OR "LLM") AND TS=("clinical notes" OR "electronic health record" OR "EHR" OR "discharge summary" OR "medical record")<br>Refined by: DOCUMENT TYPES=(Article OR Proceedings Paper)<br>Timespan: 2020–2025<br>Languages: English
IEEE Xplore	("ICD-10 coding" OR "automatic ICD-10" OR "clinical coding" OR "medical coding") AND ("rule-based" OR "machine learning" OR "neural network" OR "deep learning" OR "transformer" OR "large language model" OR "LLM") AND ("clinical notes" OR "electronic health record" OR "EHR" OR "discharge summary" OR "medical record")<br>Filters: Year: 2020–2025; Content Type: Journals & Conferences; Language: English
ACM Digital Library	Abstract: ("ICD-10 coding" OR "automatic ICD-10" OR "clinical coding" OR "medical coding") AND Abstract: ("rule-based" OR "machine learning" OR "neural network" OR "deep learning" OR "transformer" OR "large language model" OR "LLM") AND Abstract: ("clinical notes" OR "electronic health record" OR "EHR" OR "discharge summary" OR "medical record")<br>Publication Date: 2020–2025
arXiv	("ICD-10 coding" OR "automatic ICD-10" OR "clinical coding" OR "medical coding") AND ("rule-based" OR "machine learning" OR "neural network" OR "deep learning" OR "transformer" OR "large language model" OR "LLM") AND ("clinical notes" OR "electronic health record" OR "EHR" OR "discharge summary" OR "medical record")<br>Date range: 2020–2025
Google Scholar	("ICD-10 coding" OR "automatic ICD-10" OR "clinical coding" OR "medical coding") ("rule-based" OR "machine learning" OR "neural network" OR "deep learning" OR "transformer" OR "large language model" OR "LLM") ("clinical notes" OR "electronic health record" OR "EHR" OR "discharge summary" OR "medical record") filetype:pdf<br>Since 2020<br>Language: English

Table S2: Overview of methodological quality and reporting practices in automatic ICD-10 coding research.

Author et al. (year)	Dataset assurance	Explainability method	Code availability	Model reproducibility	Ethics Approval	Generalizability
Wu et al. (1)	Expert-reviewed gold-standard labels	Attention visualization	Publicly available	Fully reproducible	Not reported / No ethics statement	Limited / Single-site
Motzfeldt et al. (2)	Expert-reviewed gold-standard labels	Rationale extraction, Human-in-the-loop	Publicly available	Fully reproducible	Implicit transparency only	Limited / Single-site
Gao et al. (3)	Multi-level QA	Attention visualization, Prototype/case-based, Human-in-the-loop	Available upon request	Limited reproducibility	Explicit ethics approval	Moderate / Internal-multi-source
Falis et al. (4)	Partially (auto-assigned ICD labels)	Attention visualization, Human-in-the-loop	Publicly available	Partial reproducibility	Explicit ethics approval	Limited / Single-site
Nguyen et al. (5)	Partially (auto-assigned ICD labels)	Attention visualization	Publicly available	Fully reproducible	Implicit transparency only	Limited / Single-site
Ngo et al. (6)	Partially (auto-assigned ICD labels)	Attention visualization	Not reported / No code available	Partial reproducibility	Explicit ethics approval	Limited / Single-site
Vassileva et al. (7)	Expert-reviewed gold-standard labels	Rationale extraction, Rule-based	Publicly available	Partial reproducibility	Implicit transparency only	High / External or cross-domain
Ponthongmak et al. (8)	Expert-reviewed gold-standard labels	Not reported	Publicly available	Partial reproducibility	Explicit ethics approval	High / External or cross-domain
Mayya et al. (9)	Expert-reviewed gold-standard labels	Attention visualization, Rationale extraction	Not reported / No code available	Partial reproducibility	Not reported / No ethics statement	Limited / Single-site
Masud et al. (10)	Partially (auto-assigned ICD labels)	Prototype/case-based	Not reported / No code available	Limited reproducibility	Explicit ethics approval	High / External or cross-domain
Schlegel et al. (11)	Partially (auto-assigned ICD labels)	Ontology-based	Not reported / No code available	Partial reproducibility	Explicit ethics approval	Limited / Single-site
Mustafa et al. (12)	Expert-reviewed gold-standard labels	Rationale extraction	Not reported / No code available	Limited reproducibility	Implicit transparency only	Limited / Single-site

Continued on next page

Table S2: Continued. Overview of methodological quality and reporting practices in automatic ICD-10 coding research.

**Author et al. (year)**	**Dataset assurance**	**Explainability method**	**Code availability**	**Model reproducibility**	**Ethics Approval**	**Generalizability**
Lenc et al. (13)	Expert-reviewed gold-standard labels	Not reported	Not reported / No code available	Limited reproducibility	Implicit transparency only	Limited / Single-site
Suvirat et al. (14)	Expert-reviewed gold-standard labels	Not reported	Not reported / No code available	Limited reproducibility	Explicit ethics approval	Limited / Single-site
Palacios et al. (15)	Expert-reviewed gold-standard labels	Not reported	Not reported / No code available	Partial reproducibility	Implicit transparency only	Limited / Single-site
Polignano et al. (16))	Expert-reviewed gold-standard labels	Not reported	Publicly available	Fully reproducible	Implicit transparency only	Limited / Single-site
Hauksson and Einarsson (17)	Partially (auto-assigned ICD labels)	Not reported	Not reported / No code available	Limited reproducibility	Explicit ethics approval	Limited / Single-site
Bou Hatoum et al. (18)	None / Not reported	Not reported	Not reported / No code available	Limited reproducibility	Implicit transparency only	Moderate / Internal-multi-source
Xu and Dray (19)	Partially (auto-assigned ICD labels)	Not reported	Not reported / No code available	Partial reproducibility	Implicit transparency only	Limited / Single-site
Makohon and Li (20)	Expert-reviewed gold-standard labels	Not reported	Not reported / No code available	Partial reproducibility	Implicit transparency only	High / External or cross-domain
Mustafa et al. (21)	Partially (auto-assigned ICD labels)	Not reported	Publicly available	Partial reproducibility	Implicit transparency only	Limited / Single-site
Wu et al. (22)	Partially (auto-assigned ICD labels)	Not reported	Not reported / No code available	Partial reproducibility	Implicit transparency only	High / External or cross-domain
Lamproudis et al. (23)	Partially (auto-assigned ICD labels)	Not reported	Publicly available	Partial reproducibility	Explicit ethics approval	Limited / Single-site
Dalloux et al. (24)	Partially (auto-assigned ICD labels)	Not reported	Not reported / No code available	Partial reproducibility	Not reported / No ethics statement	Limited / Single-site

Table S3: Summary of Study Objectives, Key Findings, Limitations, and Future Research Directions.

Author et al. (year)	Objectives	Key findings	Limitations	Further directions
Wu et al. (1)	This study aimed to develop GoM-ICD, an automatic ICD coding framework designed to improve the accuracy and efficiency of assigning standardized codes to electronic medical records.	GoM-ICD achieved a micro-F1 score of 0.613 on the MIMIC-IV ICD-10 benchmark. Its gap schemes improved long-document handling, while the multi-level MoE architecture and RoBERTa-PM-based text-label matching enhanced coding accuracy and text-code alignment.	A key limitation is the remaining clinical readiness gap. Current models are not yet sufficient for direct practical deployment, as medical coding often requires expert judgment, specialist collaboration, and human review.	Future research should evaluate GoM-ICD on larger, more diverse clinical datasets to assess its generalizability, robustness, and scalability, as well as its integration into real-world workflows, including human-in-the-loop review, error monitoring, auditability, and clinical acceptability.
Motzfeldt et al. (2)	The main objective is to introduce Code Like Humans (CLH), a novel multi-agent framework leveraging Large Language Models (LLMs) to emulate human medical coding workflows by integrating official ICD-10 guidelines and external resources. This agentic approach is designed to support the complete ICD-10 coding system over 70K labels and seeks to serve as an assistive tool for human coders rather than an autonomous replacement.	The proposed CLH framework (a multi-agent LLM) achieves performance comparable to PLM-ICD while covering the entire ICD-10 label space, approximately 70K codes. It improves macro-F1 on rare codes and enables interpretable, reasoning-based predictions aligned with human coding workflows.	The evaluation relies on English, single institution datasets, including MIMIC-III and MDACE, and lacks external or multilingual validation. CLH's end-to-end precision remains lower than traditional classifiers, limiting readiness for full automation.	Future work will focus on human-in-the-loop integration and fine-tuning each agent for better evidence extraction, retrieval, and verification. The authors also plan to explore interactive interfaces and broader clinical validation across languages and healthcare settings.

Continued on next page

Table S3: Continued. Summary of Study Objectives, Key Findings, Limitations, and Future Research Directions.

Author et al. (year)	Objectives	Key findings	Limitations	Further directions
Gao et al. (3)	This study proposes CliniCoCo, a novel Human-in-the-Loop (HITL) framework, focusing on how automated clinical coding systems and human coders can work effectively and efficiently together in real-world settings. The ultimate goal is to establish a general paradigm for human–AI collaborative clinical coding that is compatible with diverse and complex clinical scenarios in Chinese hospitals.	The proposed CliniCoCo HITL framework achieved high ICD-10 coding accuracy, with micro-F1 between 0.80 and 0.84, AUC between 0.93 and 0.97, and reduced manual coding time by about 40%. Human–AI collaboration improved both efficiency and interpretability through attention visualization and similar-case retrieval.	The model was evaluated only on Chinese EMRs from two hospitals, limiting cross-lingual generalizability. The pilot usability test involved a small number of coders and lacked full clinical deployment.	Future work should extend validation across multiple institutions and languages to assess generalizability. Integration into real-world hospital workflows and long-term human–AI co-learning studies are planned to evaluate sustainability and clinical impact.
Falis et al. (4)	The primary aim of this study was to investigate GPT-3.5 in generating and coding medical documents with ICD-10 codes for data augmentation, particularly addressing issues related to low-resource labels. It also sought to evaluate GPT-3.5's viability as an automated ICD-coding classifier and a producer of clinically accurate synthetic discharge summaries.	GPT-3.5 performs poorly on real clinical notes but achieves better accuracy on its own synthetic data. Synthetic augmentation improves lower-resource ICD-10 code performance and reduces out-of-family errors.	The study is limited to English MIMIC-IV data and lacks external validation. GPT-3.5 was not fine-tuned, and only four clinicians evaluated the generated texts.	Future work should explore the potential of LLM-generated discharge summaries using different LLMs, prompt engineering, and supplementing prompts with external knowledge, such as ontologies. Solutions to enhance clinical realism include restructuring the prompt to order diagnoses chronologically with time-stamps or retrieving real clinical notes as in-context learning examples to guide generation.

Continued on next page

Table S3: Continued. Summary of Study Objectives, Key Findings, Limitations, and Future Research Directions.

Author et al. (year)	Objectives	Key findings	Limitations	Further directions
Nguyen et al. (5)	The primary objective is to propose a public benchmark suite for ICD-10 coding using a large Electronic Health Record dataset derived from MIMIC-IV. This aims to standardize data preprocessing and model comparison, fostering reproducibility and accelerating progress in automated ICD coding research.	The study introduces MIMIC-IV-ICD, a large benchmark dataset for ICD-9 and ICD-10 coding, enabling standardized evaluation across models. Among tested baselines, PLM-ICD performs best on full-code settings, while MSMN excels on top-50 frequent codes.	The dataset is limited to English clinical notes from a single institution, which may restrict generalizability. It focuses solely on text-based discharge summaries, excluding multimodal or real clinical validation.	Future work plans to expand the benchmark by adding more baselines and potentially incorporating relevant features such as drug codes and patient vitals. The study also aims to extend the use of clinical notes for the joint prediction of ICD codes and tasks like readmission, triage, and mortality prediction.
Ngo et al. (6)	This study introduces and fine-tunes two versions of the domain-specific Norwegian BERT-based model, NorDeClin-BERT, for predicting ICD-10 diagnosis codes in Norwegian clinical text. The primary objective was to evaluate how domain-specific pretraining and model size impact the classification performance compared to general-purpose and cross-lingual BERT models.	Domain-specific pretraining on Norwegian clinical text, using NorDeClin-BERT, substantially improved ICD-10 coding performance over general and cross-lingual models. The large variant achieved the best overall results, while the base model remained highly efficient and competitive.	The study was limited to one hospital and one medical domain (gastroenterology). External validation across other specialties or institutions was not conducted.	Future work should evaluate NorDeClin-BERT on multi-domain and cross-hospital datasets. Integrating explainable AI and real-world clinical testing is recommended to enhance trust and usability.

Continued on next page

Table S3: Continued. Summary of Study Objectives, Key Findings, Limitations, and Future Research Directions.

Author et al. (year)	Objectives	Key findings	Limitations	Further directions
Vassileva et al. (7)	This paper proposes and evaluates a fully unsupervised, multilingual approach for linking clinical entities to ICD-10 codes using a multistage pipeline. The objective is to demonstrate the system's effectiveness without relying on complex dictionary-based matching for unambiguous terms with in-context learning using GPT-4.1 on benchmark datasets in Spanish and Greek.	The hybrid approach combining ICD-10 dictionaries with GPT-4.1 achieved strong multilingual performance, with F1 = 0.85 on Greek (EICardioCC) and F1 = 0.89/0.78 on Spanish (CodiEsp) without any fine-tuning, showing that LLMs can effectively perform ICD-10 entity linking in multiple languages.	The study was limited to two languages, Spanish and Greek, and relied on Azure OpenAI models, which are expensive and cannot be deployed locally in hospitals. Its performance may vary across languages and datasets.	Future work will explore fine-tuning open-source or smaller LLMs for local deployment and efficiency, and extend the system to end-to-end term detection and linking to improve clinical usability.
Ponthongmak et al. (8)	The objective of this study was to develop an automated ICD coding tool, specifically the CNN–PubMedBERT model, using deep learning and NLP techniques applied to discharge summary texts translated from Thai–English clinical data. A secondary goal was the external validation of this model using MIMIC-III data, including ICD-10 and ICD-9, to assess its prediction performance and generalizability in different healthcare cohorts.	The CNN–PubMedBERT model achieved strong ICD-10 coding performance and outperformed conventional CNN and NB baselines. Fine-tuning on external datasets, MIMIC-ICD10/ICD9, improved cross-domain generalizability and model robustness.	The model was trained on only the 50 most frequent ICD-10 codes, limiting clinical coverage and real-world applicability. Mixed Thai–English text and translation errors may have affected data quality and model accuracy.	Future studies should extend the scope to include all ICD-10 codes and should combine text and structured data in hybrid deep learning models to improve performance. Further external validation is needed in other Thai hospitals where documentation is typically in Thai, aiming for eventual clinical utility as a web-based or mobile application to aid diagnostic classification.

Continued on next page

Table S3: Continued. Summary of Study Objectives, Key Findings, Limitations, and Future Research Directions.

Author et al. (year)	Objectives	Key findings	Limitations	Further directions
Mayya et al. (9)	This paper introduces LATA, a neural model based on Label Attention Transformer Architectures, designed for the automatic assignment of ICD-10 codes from unstructured patient case reports. The core objective is to improve predictive performance over basic BERT variants and provide explainability by directly extracting textual evidence associated with the predicted diagnostic code.	The proposed LATA model outperformed all baseline BERT variants by 33–49% in F1 and MAP scores. Its label attention mechanism improved both predictive accuracy and interpretability by highlighting text evidence linked to ICD-10 codes.	Performance dropped for rare ICD-10 codes with very few training samples. The study used only diagnostic codes from a small, single-source dataset, limiting generalizability.	Future work will extend LATA to procedural codes, incorporate large-scale pretraining, and evaluate multilingual or cross-domain ICD coding.
Masud et al. (10)	The main objective was to develop and validate a deep learning-based natural language processing model, called Deep-ADCA, to accurately predict ICD-10 codes using clinical notes in electronic medical records. This model aims to assist physicians in selecting appropriate diagnosis codes at the time of ordering medication, thereby reducing the manual coding workload and enhancing the efficiency of clinical coding.	The CNN-based Deep-ADCA model accurately predicted ICD-10 codes from EMR clinical notes using medication lists, achieving clinically satisfactory F1-scores of 0.61–0.78. It also identified missing diagnoses, demonstrating potential to support physicians and improve coding accuracy.	The model was trained on a single-hospital dataset with limited sample size and five departments only. No external validation or baseline model comparison was performed.	Future work should include external, multi-center validation and larger, more diverse datasets. Adding advanced architectures and more features could further improve performance and generalizability.

Continued on next page

Table S3: Continued. Summary of Study Objectives, Key Findings, Limitations, and Future Research Directions.

Author et al. (year)	Objectives	Key findings	Limitations	Further directions
Schlegel et al. (11)	This paper aims to investigate the feasibility of applying state-of-the-art deep learning clinical coding approaches to the outpatient setting, a task previously overlooked. The study evaluates the models' potential to assist human coders and proposes a flexible architecture, OPD-Reranker, to exploit additional structured and unstructured information for improved performance.	Deep learning models developed for inpatient ICD coding can be effectively adapted to outpatient settings, with OPD-Reranker achieving the best performance, with micro-F1 = 63.1 and recall@5 = 80.1. Domain-specific pretraining and data cleaning (duplicate and rare-code removal) significantly improved stability, efficiency, and coding accuracy.	The dataset is from a single hospital in Taiwan, which limits generalizability across institutions or languages. ICD-10 codes were assigned by physicians with only partial review, leading to potential label noise and approximately 20% inconsistency.	Replicate the study on multi-center outpatient datasets to validate generalizability. Develop automated label-quality checks and interpretability methods to detect inconsistent or erroneous coding in real-world clinical workflows.
Mustafa et al. (12)	This study aims to systematically benchmark the ability of diverse Large Language Models (LLMs), particularly reasoning-enhanced models, to classify hierarchical ICD-10 codes from discharge summaries. The objective is to evaluate the impact of structured reasoning on performance and determine if LLMs can achieve acceptable accuracy for classifying primary and all diagnoses across different levels of ICD-10 granularity.	Reasoning-based LLMs, for example Gemini 2.5 Pro, outperformed non-reasoning models in ICD-10 classification, especially at higher hierarchical levels. Performance decreased with increasing code specificity, showing the challenge of fine-grained medical coding.	The study used a small, balanced subset of 1,500 records and only the top 10 ICD-10 codes from a single dataset (MIMIC-IV). All evaluations were zero-shot without fine-tuning or external validation.	Future work should focus on domain-specific fine-tuning, hybrid reasoning-rule models, and larger, more diverse datasets. Integrating structured clinical data and exploring multimodal or ensemble approaches may enhance coding accuracy.

Continued on next page

Table S3: Continued. Summary of Study Objectives, Key Findings, Limitations, and Future Research Directions.

Author et al. (year)	Objectives	Key findings	Limitations	Further directions
Lenc et al. (13)	The main goal of this research is to propose and implement a Medical Coding Assistant (MCA) system for the Czech language that automatically predicts diagnosis ICD codes to enhance coder efficiency and accuracy. This involved designing and evaluating low-parameter transformer-based models, including a novel four-headed architecture, suitable for rapid deployment in Czech hospitals with limited computational resources.	The proposed Four-headed transformer and mini-PLM-ICD models achieved high performance on Czech ICD-10 coding, with the mini-PLM-ICD reaching the best F1 score of 81.0%. Domain-specific pretraining and multi-task design improved both accuracy and computational efficiency.	The dataset is single-site and not publicly available, limiting reproducibility and external validation. No statistical significance testing or detailed error analysis was performed.	Future work will include integrating data from multiple hospitals to improve generalizability and balance the label distribution. The authors also plan to explore generative and data augmentation methods for enhanced performance and robustness.
Suvirat et al. (14)	This study investigates the effectiveness of classical supervised learning and multilingual BERT models for automatically predicting ICD-10 blocks from Thai free-text discharge summaries using a large institutional dataset. The main objective is to contribute information on ICD classification in the Thai clinical domain and demonstrate the potential for implementing an ICD coding assistance tool.	Multilingual BERT achieved the best performance, with macro F1 = 0.76 for top 500 blocks, outperforming traditional models on Thai–English mixed clinical notes. It demonstrated strong capability even for less frequent ICD-10 blocks.	The model was trained on a single institutional dataset and only predicted block-level ICD-10 codes. BERT's 512-token limit also restricted long-text handling.	Future work should extend to full ICD-10 code prediction and cross-institutional training. Pre-training domain-specific Thai medical language models could further enhance accuracy and generalizability.

Continued on next page

Table S3: Continued. Summary of Study Objectives, Key Findings, Limitations, and Future Research Directions.

Author et al. (year)	Objectives	Key findings	Limitations	Further directions
Palacios et al. (15)	This study evaluates the capabilities of open-access Small Language Models (SLMs) for automated ICD-10 diagnosis coding using the MIMIC-IV dataset. It specifically aims to benchmark the performance of fine-tuned cross-domain and domain-specialized biomedical SLMs using parameter-efficient fine-tuning methods.	Fine-tuned Small Language Models can perform ICD-10 coding effectively, with Phi-3-Mini-4K-Instruct achieving the best F1-score of 0.72. Longer context windows improved accuracy more than model size or domain specialization.	The study used only the Top-50 ICD-10 codes from MIMIC-IV and was limited by a 2048-token input window. Human-coded labels may contain inconsistencies, introducing bias in evaluation.	Future work should explore models with larger context windows and systematically evaluate threshold optimization techniques to improve classification performance. Researchers should also incorporate hierarchical evaluation metrics that assign lower penalties for misclassifications remaining within the same ICD-10 code family to better capture clinically relevant coding accuracy.
Polignano et al. (16)	The main objective was to develop and investigate different machine learning strategies for creating an automatic system capable of clinical coding, associating ICD-10 codes with Spanish clinical reports, specifically participating in the CodiEsp 2020 challenge. Through experimentation, the authors aimed to identify the optimal hybrid approach, which was ultimately a two-step model using BERT for pre-filtering sentences and BiLSTM-CNN-SelfAttention for final classification, to achieve high performance in this complex, multi-label task.	A hybrid model combining BERT (BETO) for sentence pre-filtering, and BiLSTM + CNN + Self-Attention for classification achieved the best results, with F1 = 0.308, MAP = 0.202. The two-stage strategy effectively improved recall and overall performance for automatic ICD-10 coding on Spanish clinical texts.	Not mentioned.	Future work expects to associate the ICD-10 code with the corresponding portion of text. This step is intended to implement a first strategy for explaining the results.

Continued on next page

Table S3: Continued. Summary of Study Objectives, Key Findings, Limitations, and Future Research Directions.

Author et al. (year)	Objectives	Key findings	Limitations	Further directions
Hauksson and Einarsson (17)	This study explores the potential of automating clinical coding in Icelandic by investigating the effectiveness and attainable performance of Transformer-based models through continued pretraining and various model adaptations. The goal is to provide insights into using Natural Language Processing (NLP) for clinical coding in low-resource languages, leveraging a large corpus of electronic health records (EHR).	MedIceBERT, a continued-pretrained Icelandic BERT model with label attention, achieved strong ICD-10 coding performance on Icelandic EHRs, comparable to English benchmarks despite limited resources.	The study faced label inaccuracies, limited computational resources, and single-run experiments, with models and data unreleased due to patient privacy restrictions.	Future work should explore patient-level aggregation, long-context architectures, and distribution-shift robustness, while addressing ethical and bias issues in real clinical deployment.
Bou Hatoum et al. (18)	The objective was to propose a semantics-aware approach utilizing custom loss functions to enhance the accuracy and clinical relevance of multi-label ICD-10 coding. This method improves performance by leveraging cosine similarity to measure the semantic relatedness between predicted and actual codes, moving beyond traditional strict equality matching.	The proposed semantic-aware loss functions, especially WHML, with approximately 0.75, improved ICD-10 coding accuracy from 74.64% to 88.54% F1-micro. Incorporating hierarchical and semantic relationships enhanced clinically relevant predictions.	The dataset was highly imbalanced and limited to a single hospital in Saudi Arabia, restricting generalizability. Rare ICD-10 codes were underrepresented, affecting performance consistency across specialties.	Future work will explore specialty-specific tuning of WHML and integration with optimized model architectures. Expanding to multi-institutional datasets and refining preprocessing could further enhance model robustness and generalizability.

Continued on next page

Table S3: Continued. Summary of Study Objectives, Key Findings, Limitations, and Future Research Directions.

Author et al. (year)	Objectives	Key findings	Limitations	Further directions
Xu and Dray (19)	The main objective is to investigate the feasibility and performance of Federated Learning (FL) for multi-label ICD code classification from clinical notes in a privacy-preserving and scalable manner. This is achieved by proposing a hybrid modular approach that decouples semantic representation using frozen LLM embeddings from classification using lightweight MLP models for collaborative training across distributed healthcare settings.	Lightweight MLP-based classifiers combined with pretrained LLM embeddings achieved strong ICD-9/10 coding performance. Federated learning preserved almost identical accuracy to centralized training, proving its feasibility for privacy-preserving clinical text modeling.	The study used only one English language dataset, MIMIC-IV-Note, without external or multilingual validation. No explainability or human evaluation was performed, limiting clinical interpretability.	Future work should test cross-hospital and multilingual datasets to evaluate real-world generalization. Integrating explainable or domain-aware components could enhance clinical trust and interpretability.
Makohon and Li (20)	The main objective is to compare various deep learning models, including attention-based networks and Hierarchical Attention Networks (HAN), for predicting ICD-10 codes using datasets derived from the MIMIC-III and CodiEsp corpora. This study aims to demonstrate that applying abbreviation normalization, spell checking, and attention mechanisms effectively enhances ICD-10 prediction performance.	The hierarchical attention model (HAN-GRU) achieved the best performance, showing that attention mechanisms effectively enhance ICD-10 code prediction accuracy. Combining MIMIC and CodiEsp datasets improved generalizability across multilingual clinical data.	Not mentioned.	The study concluded that the deep learning attention mechanism effectively improves ICD-10 prediction performance, with the Hierarchical Attention Network GRU (HAN GRU) yielding the best results. Specific further directions exploring Transformer models or real-world clinical validation are not explicitly detailed in the provided excerpts.

Continued on next page

Table S3: Continued. Summary of Study Objectives, Key Findings, Limitations, and Future Research Directions.

Author et al. (year)	Objectives	Key findings	Limitations	Further directions
Mustafa et al. (21)	The objective of this study is to evaluate the performance and consistency of Large Language Models (LLMs) in classifying clinical discharge summaries based on ICD-10 codes, comparing reasoning and non-reasoning architectures. It aims to determine how effectively these models identify and classify clinical patterns to improve automated clinical coding accuracy and enhance decision support.	Reasoning LLMs achieved higher accuracy, 71%, and F1 score, 67%, than non-reasoning models, showing stronger performance in complex ICD-10 classifications. However, non-reasoning models demonstrated greater consistency, 91%, indicating higher stability across runs.	The study used only the top 10 ICD-10 codes from MIMIC-IV and a small balanced dataset, 3,000 samples, limiting generalizability. No multi-label or external validation was performed.	Future work should expand to full multi-label ICD-10 coding, include larger and multi-institutional datasets, and explore ensemble or hybrid approaches combining reasoning and non-reasoning models.
Wu et al. (22)	The primary objective is to propose and evaluate a novel pseudo label-wise attention mechanism to significantly reduce computational redundancy and cost in multi-label automatic ICD coding by merging similar ICD codes. Additionally, the paper aims to present a convenient BERT-based method for obtaining ICD vectors, thereby enabling better prediction of rare and new ICD codes that may not appear in the training set.	The proposed LD-PLAM model with pseudo label-wise attention achieved state-of-the-art performance on both MIMIC-III and Xiangya datasets, reducing GPU memory usage by over 70%. It effectively handled rare and unseen ICD codes through its hierarchical attention and similarity-based prediction design.	Not mentioned.	To address the mediocre performance on the R@5 metric and improve fine-grained recall of positive ICD codes, further precise reordering methods could be explored. Additionally, the pseudo label-wise attention mechanism is a general architecture that should be applicable to other multi-label classification tasks.

Continued on next page

Table S3: Continued. Summary of Study Objectives, Key Findings, Limitations, and Future Research Directions.

Author et al. (year)	Objectives	Key findings	Limitations	Further directions
Lamproudis et al. (23)	The aim of this study is to introduce an openly available clinical dataset (Corpus II) to train deep learning models for predicting ICD-10 diagnosis codes in Swedish discharge summaries, specifically focusing on the gastrointestinal surgery domain. It demonstrates that models fine-tuned on this corpus achieve performance sufficient for practical use as a code recommendation tool for human coders.	SweDeClin-BERT, a Swedish clinical BERT model, achieved strong ICD-10 coding performance with an F1-score of 0.94 using the large Corpus II dataset. Larger and domain-specific data, along with Top-5 prediction, greatly improved coding accuracy and practical usability.	Not mentioned.	The model shows high potential as a coding recommendation tool by accurately reducing the code space. Future work should focus on integrating this high-accuracy classifier into real clinical settings to validate its impact on coder efficiency and workload.
Dalloux et al. (24)	The aim of this work is two-fold: to present a corpus of French clinical documents annotated with ICD-10 codes, and to propose and evaluate neural network-based methods for document-level classification. The goal is to create a tool that suggests relevant codes to human coders, given the complexity and difficulty of this task using clinical narratives (free text).	CNN models achieved higher F1-scores than BiLSTM for multi-label ICD-10 coding, with better results when codes were reduced to 2-digit blocks. Using domain-specific fastText embeddings improved overall performance on French clinical narratives.	Not mentioned.	Future work will integrate structured clinical data and explore modern contextual embeddings like BERT to enhance coding accuracy and representation quality.

Table S4: Dataset composition and label space characteristics of included studies.

**Author et al. (year)**	**Dataset name**	**Dataset language**	**ICD-10 version**	**Dataset size**	**Training dataset size**	**Labels**
Wu et al. (1)	MIMIC-IV only	English	ICD-10	122,279	89,098	7,942
Motzfeldt et al. (2)	MIMIC-III (ICD-10 mapped)	English	ICD-10-CM	1,000–11,500	1,000–7,500	50–1,000
Gao et al. (3)	Hospital EHR	Chinese	ICD-10	9,514–14,104	5,708–8,462	50–671
Falis et al. (4)	MIMIC-IV only, Combined Datasets	English	ICD-10-CM , ICD-10-PCS	9,606–131,916	0–120,048	195–25,230
Nguyen et al. (5)	MIMIC-IV only	English	ICD-10-CM	104,077–122,317	104,077–110,442	50–26,096
Ngo et al. (6)	Domain-specific	Norwegian	ICD-10	87,938	70,350	350–500
Vassileva et al. (7)	Benchmark Datasets	Greek, Spanish	ICD-10	1,000	1,000	2,000–14,000
Ponthongmak et al. (8)	Hospital EHR, MIMIC-III (ICD-10 mapped)	Thai translated to English, English	ICD-10	15,329–40,923	9,197–24,554	50
Mayya et al. (9)	Benchmark Datasets	Mixed: English and Spanish	ICD-10	1,000	695	2,194
Masud et al. (10)	Hospital EHR	English	ICD-10	2,762–6,027	2,485–5,424	148–475
Schlegel et al. (11)	Hospital EHR	Mixed: English and Chinese	ICD-10	2,381,000	2,381,000	2,588
Mustafa et al. (12)	MIMIC-IV only	English	ICD-10	1,500	1,500	10
Lenc et al. (13)	Hospital EHR	Czech	ICD-10	122,279–432,279	89,141–376,515	1,538–7,942
Suvirat et al. (14)	Hospital EHR	Mixed: English and Thai	ICD-10-TM	91,756–405,122	73,404–324,098	8,650
Palacios et al. (15)	MIMIC-IV only	English	ICD-10	104,076	7,368	50
Polignano et al. (16)	Benchmark Datasets	Spanish	ICD-10-CM , ICD-10-PCS	1,000	500	546–1,788

Continued on next page

Table S4: Continued. Dataset composition and label space characteristics of included studies.

Author et al. (year)	Dataset name	Dataset language	ICD-10 version	Dataset size	Training dataset size	Labels
Hauksson and Einarsson (17)	Hospital EHR	Icelandic	ICD-10	16,000,000	14,400,000	50–8,922
Bou Hatoum et al. (18)	Hospital EHR	English	ICD-10-AM	9,570,000	9,570,000	3,100
Xu and Dray (19)	MIMIC-IV only	English	ICD-10	121,344	84,941	10–1,085
Makohon and Li (20)	MIMIC-III (ICD-10 mapped), Combined Datasets	English, Mixed: English and Spanish	ICD-10-CM , ICD-10-PCS	52,722–59,930	36,905–41,951	5,891–7,658
Mustafa et al. (21)	MIMIC-IV only	English	ICD-10	3,000	3,000	10
Wu et al. (22)	Hospital EHR	Chinese	ICD-10	7,732	6,186	50–1,177
Lamproudis et al. (23)	Domain-specific	Swedish	ICD-10	6,062–81,089	5,456–72,980	10–415
Dalloux et al. (24)	Hospital EHR	French	ICD-10	28,000	22,400	6,116

Table S5: Publications employing specific text preprocessing techniques.

Author et al. (year)	Subword tokenization	Basic Cleaning	Word-level tokenization	Sentence segmentation	Medical normalization	Multilingual tokenization	Stemming
Wu et al. (1)	✓	✓	—	—	—	—	—
Motzfeldt et al. (2)	✓	—	—	—	—	—	—
Gao et al. (3)	✓	—	—	—	—	—	—
Falis et al. (4)	—	✓	✓	—	—	—	—
Nguyen et al. (5)	—	✓	✓	—	—	—	—
Ngo et al. (6)	✓	—	—	—	—	—	—
Vassileva et al. (7)	—	—	—	—	—	✓	—
Ponthongmak et al. (8)	✓	✓	✓	✓	—	—	—
Mayya et al. (9)	✓	✓	—	✓	—	—	✓
Masud et al. (10)	—	✓	✓	—	—	—	✓
Schlegel et al. (11)	✓	✓	✓	—	—	✓	—
Mustafa et al. (12)	—	✓	—	—	✓	—	—
Lenc et al. (13)	✓	✓	—	—	—	—	—
Suvirat et al. (14)	✓	✓	✓	—	—	✓	—
Palacios et al. (15)	✓	—	—	—	—	—	—
Polignano et al. (16)	✓	—	✓	✓	—	—	—
Hauksson and Einarsson (17)	✓	✓	—	—	—	—	—
Bou Hatoum et al. (18)	✓	—	—	—	✓	—	—
Xu and Dray (19)	✓	—	—	—	—	✓	—
Makohon and Li (20)	—	✓	✓	—	✓	—	—
Mustafa et al. (21)	—	✓	—	✓	✓	—	—
Wu et al. (22)	—	✓	✓	—	—	—	—
Lamproudis et al. (23)	✓	—	—	—	—	—	—
Dalloux et al. (24)	—	—	✓	—	—	—	—

Table S6: Publications reporting feature representation techniques.

Author et al. (year)	Contextual embedding	Static word embedding	Domain-specific embedding	LLM embedding	TF-IDF	Sentence or document embedding	BoW
Wu et al. (1)	✓	—	✓	✓	—	—	—
Motzfeldt et al. (2)	✓	—	✓	✓	—	—	—
Gao et al. (3)	✓	—	—	—	—	—	—
Falis et al. (4)	—	✓	—	✓	—	—	—
Nguyen et al. (5)	✓	✓	✓	—	—	✓	—
Ngo et al. (6)	✓	—	✓	—	—	—	—
Vassileva et al. (7)	✓	—	—	✓	—	—	—
Ponthongmak et al. (8)	✓	✓	✓	—	✓	—	—
Mayya et al. (9)	✓	—	—	—	—	—	—
Masud et al. (10)	—	✓	—	—	✓	—	—
Schlegel et al. (11)	✓	✓	✓	—	—	✓	—
Mustafa et al. (12)	✓	—	—	✓	—	—	—
Lenc et al. (13)	✓	—	✓	—	—	—	—
Suvirat et al. (14)	✓	—	—	—	✓	—	✓
Palacios et al. (15)	✓	—	✓	✓	—	—	—
Polignano et al. (16)	✓	✓	—	—	—	—	—
Hauksson and Einarsson (17)	✓	—	✓	—	—	—	—
Bou Hatoum et al. (18)	✓	—	✓	—	—	—	—
Xu and Dray (19)	✓	—	—	✓	—	✓	—
Makohon and Li (20)	—	✓	—	—	—	—	—
Mustafa et al. (21)	✓	—	—	✓	—	—	—
Wu et al. (22)	—	✓	—	—	—	—	—
Lamproudis et al. (23)	✓	—	✓	—	—	—	—
Dalloux et al. (24)	—	✓	—	—	—	—	—

Table S7: Model training strategies and corresponding main and baseline models.

Author et al. (year)	Training Strategies	Main Model	Baseline Model
Wu et al. (1)	Train-val-test split,<br>Stratified sampling,<br>Threshold tuning,<br>Fine-tuning,<br>Multi-stage	**Hybrid DL**<br>GoM-ICD	**Hybrid DL**<br>Gap 0,<br>Gap 1,<br>Gap 2
Motzfeldt et al. (2)	Train-val-test split,<br>Threshold tuning,<br>Fine-tuning,<br>Multi-stage	**Hybrid DL**<br>CLH-small,<br>CLH-base,<br>CLH-large, CLH-o3-mini, CLH-o4-mini, CLH-base	**LLM**<br>Llama3-70B,<br>Llama-3.2-1B,<br>**Hybrid LLM**<br>Llama-3.2-1B + Label-wise attention
Gao et al. (3)	Train-val-test split,<br>Threshold tuning,<br>Fine-tuning,<br>Multi-stage	**Hybrid DL**<br>CliniCoCo	**Deep Learning**<br>CAML,<br>LAAT,<br>**Hybrid DL**<br>Joint LAAT,<br>**Transformer**<br>BERT-based model (Chen et al.)
Falis et al. (4)	Train-val-test split	**LLM**<br>GPT-3.5 real,<br>GPT-3.5 synthetic	**Deep Learning**<br>CAML base,<br>CAML aug,<br>LAAT base,<br>LAAT aug,<br>MRCNN base,<br>MRCNN aug

Continued on next page

Table S7: Continued. Model training strategies and corresponding main and baseline models.

Author et al. (year)	Training Strategies	Main Model	Baseline Model
Nguyen et al. (5)	Train-val-test split,<br>Multi-stage	**Hybrid DL**<br>PLM-ICD	**Deep Learning**<br>CAML,<br>LAAT,<br>MSMN,<br>**Hybrid DL**<br>Joint LAAT
Ngo et al. (6)	Train-val-test split,<br>Fine-tuning,<br>Incremental,<br>Threshold tuning	**Transformer**<br>NorDeClin-BERT-large-NorICD,<br>NorBERT3-large-NorICD,<br>NorDeClin-BERT-base-NorICD,<br>NorBERT3-large-NorICD	**Transformer**<br>NorDeClin-BERT-base-NorICD,<br>SweDeClin-BERT-SweICD,<br>SweDeClin-BERT-NorICD,<br>ScandiBERT-NorICD,<br>NorBERT3-base-NorICD,<br>NorDeClin-BERT-base-NorICD,<br>SweDeClin-BERT-SweICD,<br>SweDeClin-BERT-NorICD,<br>ScandiBERT-NorICD,<br>NorBERT3-base-NorICD
Vassileva et al. (7)	Multi-stage	**LLM**<br>GPT-4o 0-shot,<br>GPT-4o 1-shot,<br>GPT-4.1 0-shot,<br>GPT-4.1 1-shot,<br>**Hybrid LLM**<br>Dict+GPT-4o 0-shot,<br>Dict+GPT-4o 1-shot,<br>Dict+GPT-4.1 0-shot,<br>Dict+GPT-4.1 1-shot	**Rule-based**<br>Dict

Continued on next page

Table S7: Continued. Model training strategies and corresponding main and baseline models.

Author et al. (year)	Training Strategies	Main Model	Baseline Model
Ponthongmak et al. (8)	Train-val-test split,<br>Threshold tuning,<br>Fine-tuning	**Deep Learning**<br>CNN-PubMedBERT	**Machine Learning**<br>NB-TF-IDF,<br>**Deep Learning**<br>CNN-NWE,<br>CNN-PB
Mayya et al. (9)	Train-val-test split	**Hybrid DL**<br>LATA-BERT,<br>LATA-ALBERT,<br>LATA-DeBERTa,<br>LATA-DistilBERT,<br>LATA-MobileBERT,<br>LATA-RoBERTa,<br>LATA-XLNet	**Transformer**<br>BERT,<br>ALBERT,<br>DeBERTa,<br>DistilBERT,<br>MobileBERT,<br>RoBERTa,<br>XLNet
Masud et al. (10)	Train-val-test split	**Deep Learning**<br>Deep-ADCA	—
Schlegel et al. (11)	Train-val-test split,<br>Threshold tuning,<br>Fine-tuning	**Hybrid DL**<br>OPD-R ERANKER	**Deep Learning**<br>CAML,<br>LAAT,<br>MSMN<br>**Hybrid DL**<br>OPD-LM-LAAT
Mustafa et al. (12)	None	**LLM**<br>Deepseek Reasoner,<br>Gemini 2.0 Flash Thinking,<br>Gemini 2.5 Pro,<br>GPT o3 Mini,<br>Qwen QWQ	—

Continued on next page

Table S7: Continued. Model training strategies and corresponding main and baseline models.

Author et al. (year)	Training Strategies	Main Model	Baseline Model
Lenc et al. (13)	Train-val-test split,<br>Stratified sampling,<br>Fine-tuning,<br>Multi-stage	**Transformer**<br>mini-transformer,<br>mini-transformer CLS+SEP,<br>mini-PLM-ICD,<br>**Hybrid DL**<br>Four-headed,<br>Four-headed+CorNet	**Transformer**<br>Small-E-Czech,<br>**Hybrid DL**<br>mini-PLM-ICD,<br>Four-headed + CorNet
Suvirat et al. (14)	Train-val-test split,<br>Threshold tuning,<br>Fine-tuning	**Transformer**<br>Multilingual BERT	**Machine Learning**<br>Bag of n-grams,<br>TF-IDF
Palacios et al. (15)	Train-val-test split,<br>Stratified sampling,<br>Threshold tuning,<br>Fine-tuning	**LLM**<br>Falcon-RW-1B,<br>Meditron-7B,<br>Phi3-Mini-4K-Instruct-3.5B	—
Polignano et al. (16)	Train-val-test split,<br>Threshold tuning,<br>Fine-tuning,<br>Multi-stage	**Deep Learning**<br>BiLSTM + CNN + SelfAtt. - BiLSTM + CNN + SelfAtt.,<br>**Transformer**<br>BERT (BETO) - BERT (BETO multi-class),<br>**Hybrid DL**<br>BiLSTM + CNN +SelfAtt. - BERT (BETO multi-class),<br>BERT (BETO) - BiLSTM + CNN + SelfAtt.,<br>BERT - BiLSTM + CNN + SelfAtt.	**Deep Learning**<br>LSTM,<br>BiLSTM,<br>BiLSTM + CNN,<br>BiLSTM + CNN + SelfAtt.,<br>CNN,<br>CNN + SelfAtt.,<br>BiLSTM+CNN+SelfAtt.,<br>**Transformer**<br>BERT (BETO),<br>BERT
Hauksson and Einarsson (17)	Train-val-test split,<br>Fine-tuning,<br>Incremental	**Transformer**<br>MedIceBERT	—

Continued on next page

Table S7: Continued. Model training strategies and corresponding main and baseline models.

Author et al. (year)	Training Strategies	Main Model	Baseline Model
Bou Hatoum et al. (18)	Cross-validation	**Hybrid DL**<br>ClinicalBERT + Custom Loss Functions (TLCL),<br>ClinicalBERT + Custom Loss Functions (PLCL),<br>ClinicalBERT + Custom Loss Functions (BHML),<br>ClinicalBERT + Custom Loss Functions (WHML @0.25),<br>ClinicalBERT + Custom Loss Functions (WHML @0.75)	**Hybrid DL**<br>ClinicalBERT + Custom Loss Functions<br>(Binary Cross-Entropy, EM)
Xu and Dray (19)	Train-val-test split,<br>Stratified sampling	**Hybrid DL**<br>Qwen3-Embed-0.6B + DeepMLP,<br>Qwen3-Embed-0.6B + DeepResMLP,<br>gte-Qwen2-1.5B-instruct + DeepMLP,<br>gte-Qwen2-1.5B-instruct + DeepResMLP,<br>gte-multilingual-base + DeepMLP,<br>gte-multilingual-base + DeepResMLP,<br>snowflake-arctic-embed-v2.0 + DeepMLP,<br>snowflake-arctic-embed-v2.0 + DeepResMLP,<br>snowflake-arctic-embed-m-v2.0 + DeepMLP,<br>snowflake-arctic-embed-m-v2.0 + DeepResMLP,<br>nomic-embed-text-v1.5 + DeepMLP,<br>nomic-embed-text-v1.5 + DeepResMLP	**Hybrid DL**<br>Qwen3-Embed-0.6B + MLP,<br>gte-Qwen2-1.5B-instruct + MLP,<br>gte-multilingual-base + MLP,<br>snowflake-arctic-embed-l-v2.0 + MLP,<br>snowflake-arctic-embed-m-v2.0 + MLP,<br>nomic-embed-text-v1.5 + MLP
Makohon and Li (20)	Train-val-test split,<br>Cross-validation	**Deep Learning**<br>LSTM Attn,<br>GRU Attn,<br>HAN LSTM,<br>HAN GRU	**Deep Learning**<br>LSTM,<br>GRU

Continued on next page

Table S7: Continued. Model training strategies and corresponding main and baseline models.

Author et al. (year)	Training Strategies	Main Model	Baseline Model
Mustafa et al. (21)	Stratified sampling	**LLM**<br>Deepseek Chat,<br>Gemini 2.0 Flash,<br>GPT 3o Mini,<br>Qwen QWQ 32B	—
Wu et al. (22)	Train-val-test split,<br>Fine-tuning	**Hybrid DL**<br>LD-PLAM	**Machine Learning**<br>MVC-LDA,<br>MVC-RLDA,<br>**Deep Learning**<br>DeepLabeler,<br>RAICD,<br>CAML,<br>MultiResCNN,<br>**Hybrid DL**<br>DR-CAML
Lamproudis et al. (23)	Train-val-test split,<br>Fine-tuning,<br>Incremental	**Transformer**<br>SweDeClin-BERT	—
Dalloux et al. (24)	Train-val-test split	**Deep Learning**<br>CNN	**Deep Learning**<br>BiLSTM

Table S8: Reporting of hyperparameters and fine-tuning configurations across included studies.

Author et al. (year)	Loss Function	Epochs	Max Sequence Length	Learning Rate	Batch Size	Optimizer	Dropout	Fine-tuning Scope	Early Stopping	Weight Decay	Scheduler
Wu et al. (1)	✓	✓	✓	✓	✓	✓	—	✓	—	—	✓
Motzfeldt et al. (2)	—	—	—	—	—	—	—	—	—	—	—
Gao et al. (3)	✓	✓	—	✓	✓	✓	—	—	—	—	—
Falis et al. (4)	—	✓	—	—	—	—	—	—	✓	—	—
Nguyen et al. (5)	—	—	✓	—	✓	—	—	—	—	—	—
Ngo et al. (6)	✓	✓	✓	✓	✓	✓	—	✓	✓	✓	✓
Vassileva et al. (7)	—	—	—	—	—	—	—	—	—	—	—
Ponthongmak et al. (8)	✓	✓	✓	✓	✓	✓	✓	✓	✓	✓	—
Mayya et al. (9)	✓	✓	✓	✓	—	✓	✓	—	✓	—	—
Masud et al. (10)	✓	—	✓	—	—	—	—	—	—	—	—
Schlegel et al. (11)	✓	✓	✓	—	✓	✓	—	✓	✓	—	—
Mustafa et al. (12)	—	—	—	—	—	—	—	—	—	—	—
Lenc et al. (13)	✓	✓	✓	✓	✓	✓	—	✓	✓	—	—
Suvirat et al. (14)	✓	✓	—	✓	—	—	—	✓	—	—	—
Palacios et al. (15)	✓	✓	✓	✓	✓	✓	✓	✓	—	✓	—
Polignano et al. (16)	✓	✓	✓	—	✓	—	✓	✓	—	—	—
Hauksson and Einarsson (17)	✓	✓	✓	—	—	—	—	✓	—	—	—
Bou Hatoum et al. (18)	✓	✓	—	✓	✓	✓	—	—	✓	—	—
Xu and Dray (19)	✓	✓	✓	✓	—	✓	✓	—	—	✓	✓
Makohon and Li (20)	✓	✓	—	✓	✓	✓	✓	—	—	—	—
Mustafa et al. (21)	—	—	—	—	—	—	—	—	—	—	—
Wu et al. (22)	✓	—	✓	✓	✓	✓	✓	—	—	—	—
Lamproudis et al. (23)	—	—	—	✓	✓	—	—	—	✓	—	—
Dalloux et al. (24)	✓	✓	—	—	—	—	✓	—	—	—	—

Table S9: Evaluation metrics reported across included automatic ICD-10 coding studies.

Author et al. (year)	F-measure	Recall	Precision	Accuracy	AUC	AUPRC	AUROC	EMR	MAP	Others	F1 usage
Wu et al. (1)	✓	✓	✓	✓	✓	—	—	—	—	—	F1-micro, F1-macro
Motzfeldt et al. (2)	✓	✓	✓	—	—	—	—	✓	—	—	F1-micro, F1-macro
Gao et al. (3)	✓	✓	—	—	✓	—	—	—	—	✓	F1-micro, F1-macro
Falis et al. (4)	✓	✓	✓	—	—	—	—	—	—	✓	F1-micro, F1-macro, F1-score*
Nguyen et al. (5)	✓	—	✓	—	✓	—	—	—	—	—	F1-micro, F1-macro
Ngo et al. (6)	✓	✓	✓	✓	—	—	—	—	—	—	F1-weighted
Vassileva et al. (7)	✓	✓	✓	—	—	—	—	—	—	—	F1-score*
Ponthongmak et al. (8)	✓	✓	✓	—	—	✓	✓	—	—	✓	F1-micro, F1-macro
Mayya et al. (9)	✓	✓	✓	—	—	—	—	—	✓	—	F1-micro
Masud et al. (10)	✓	✓	✓	—	—	—	—	—	—	—	F1-score*
Schlegel et al. (11)	✓	✓	—	—	✓	—	—	—	—	✓	F1-micro, F1-macro
Mustafa et al. (12)	✓	—	—	—	—	—	—	—	—	—	F1-macro
Lenc et al. (13)	✓	✓	✓	✓	—	—	—	✓	—	—	F1-micro, F1-macro
Suvirat et al. (14)	✓	✓	✓	—	—	—	—	✓	—	—	F1-macro
Palacios et al. (15)	✓	—	—	—	✓	—	—	—	—	—	F1-micro, F1-macro, F1-weighted
Polignano et al. (16)	✓	✓	✓	—	—	—	—	—	✓	—	F1-macro
Hauksson and Einarsson (17)	✓	—	—	—	✓	—	—	—	—	—	F1-micro, F1-macro
Bou Hatoum et al. (18)	✓	—	—	—	—	—	—	—	—	—	F1-micro, F1-weighted
Xu and Dray (19)	✓	✓	✓	—	—	—	—	—	—	—	F1-micro, F1-macro, F1-score*
Makohon and Li (20)	✓	✓	✓	—	✓	—	—	—	—	✓	F1-micro, F1-macro
Mustafa et al. (21)	✓	—	—	✓	—	—	—	—	—	✓	F1-macro
Wu et al. (22)	✓	✓	✓	—	✓	✓	—	—	—	—	F1-micro
Lamproudis et al. (23)	✓	✓	✓	—	—	—	—	—	—	—	F1-score*
Dalloux et al. (24)	✓	✓	✓	—	—	—	—	—	—	—	F1-score*

Note. F1-score*: F1-score; averaging method not specified.

Table S10: Label approach and code granularity by study.

Author et al. (year)	Label approach	Full codes	3-char	4-char	Block codes
Wu et al. (1)	All codes	✓	—	—	—
Motzfeldt et al. (2)	All codes	✓	—	—	—
	Top-50 codes	✓	—	—	—
Gao et al. (3)	All codes	✓	—	—	—
	Top-50 codes	✓	—	—	—
	Top-100 codes	✓	—	—	—
Falis et al. (4)	All codes	✓	—	—	—
Nguyen et al. (5)	All codes	✓	—	—	—
	Top-50 codes	✓	—	—	—
Ngo et al. (6)	All codes	✓	—	—	—
	Top 80%	✓	—	—	—
Vassileva et al. (7)	All codes	—	✓	✓	—
Ponthongmak et al. (8)	Top-50 codes	✓	—	—	—
Mayya et al. (9)	All codes	✓	—	—	—
Masud et al. (10)	All codes	✓	—	—	—
Schlegel et al. (11)	All codes	✓	—	—	—
Mustafa et al. (12)	Top-10 codes	✓	✓	✓	—
Lenc et al. (13)	All codes	✓	✓	✓	—
Suvirat et al. (14)	Top-100 codes	—	✓	—	—
	Top-300 codes	—	✓	—	—
	Top-500 codes	—	✓	—	—
Palacios et al. (15)	Top-50 codes	✓	—	—	—

Continued on next page

Table S10: Continued. Label approach and code granularity by study.

Author et al. (year)	Label approach	Full codes	3-char	4-char	Block codes
Polignano et al. (16)	All codes	✓	—	—	—
Hauksson and Einarsson (17)	All codes	✓	—	—	—
	Top-50 codes	✓	—	—	—
	Top-100 codes	✓	—	—	—
Bou Hatoum et al. (18)	All codes	✓	—	—	—
Xu and Dray (19)	All codes	✓	—	—	—
	Top-10 codes	✓	—	—	—
	Top-50 codes	✓	—	—	—
Makohon and Li (20)	All codes	✓	—	—	—
Mustafa et al. (21)	Top-10 codes	—	✓	—	—
Wu et al. (22)	All codes	✓	—	—	—
	Top-50 codes	✓	—	—	—
Lamproudis et al. (23)	All codes	✓	—	—	—
	Top 80%	✓	—	—	—
	Block level	—	—	—	✓
	Block level (Top 80%)	—	—	—	✓
Dalloux et al. (24)	All codes	✓	✓	—	—

Table S11: Appropriateness levels and class imbalance handling strategies across included studies.

**Author et al. (year)**	**Appropriateness**	**Hierarchical***	**Threshold tuning***	**Specialized loss**	**Undersampling**	**Class weighting**	**Data augmentation**	**Curriculum***
Wu et al. (1)	Long-tail	✓	✓	—	—	—	✓	—
Motzfeldt et al. (2)	Extreme sparsity, Robust learning	✓	✓	—	—	—	—	—
Gao et al. (3)	Robust learning, Acceptable, Long-tail	✓	✓	✓	—	✓	—	✓
Falis et al. (4)	Extreme sparsity, Long-tail	—	—	—	—	—	✓	—
Nguyen et al. (5)	Extreme sparsity, Extremely strong	✓	—	✓	—	—	—	—
Ngo et al. (6)	Robust learning	—	✓	—	—	—	—	—
Vassileva et al. (7)	Extreme sparsity	—	—	—	—	—	—	—
Ponthongmak et al. (8)	Robust learning	✓	✓	—	—	—	—	—
Mayya et al. (9)	Extreme sparsity	✓	—	✓	—	—	✓	—
Masud et al. (10)	Acceptable, Long-tail	—	—	—	—	—	—	—
Schlegel et al. (11)	Robust learning	✓	✓	—	✓	—	—	—
Mustafa et al. (12)	Robust learning	—	—	—	✓	—	—	—
Lenc et al. (13)	Robust learning, Acceptable, Long-tail	✓	—	—	—	—	—	—
Suvirat et al. (14)	Long-tail	—	✓	✓	—	—	—	—
Palacios et al. (15)	Robust learning	—	✓	✓	—	✓	—	—
Polignano et al. (16)	Extreme sparsity	✓	✓	—	—	—	—	—
Hauksson and Einarsson (17)	Extremely strong	✓	—	—	—	—	—	—
Bou Hatoum et al. (18)	Extremely strong	—	—	✓	—	—	—	—

Continued on next page

Table S11: Continued. Appropriateness levels and class imbalance handling strategies across included studies.

Author et al. (year)	Appropriateness	Hierarchical*	Threshold tuning*	Specialized loss	Undersampling	Class weighting	Data augmentation	Curriculum*
Xu and Dray (19)	Acceptable, Extremely strong	—	—	✓	—	—	—	—
Makohon and Li (20)	Long-tail	✓	—	—	—	—	—	—
Mustafa et al. (21)	Robust learning	—	—	—	✓	—	—	—
Wu et al. (22)	Long-tail, Robust learning	✓	—	—	—	—	—	—
Lamproudis et al. (23)	Robust learning, Extremely strong	—	—	—	✓	—	—	—
Dalloux et al. (24)	Extreme sparsity	✓	—	—	—	—	—	—

Note. Hierarchical*: Hierarchical or attention architecture. Threshold tuning*: Threshold tuning or calibration. Curriculum*: Curriculum or staged training.
Documents per label: Extreme sparsity (< 5), Long-tail (5–20), Acceptable (20–100), Robust learning (100–1,000), Extremely strong ($> 1,000$).

Table S12: Summary of experimental settings and performance across ICD-10 coding studies.

Author et al. (year)	Modeling Role	Dataset name	Language	Training Dataset Size	Number of Labels	Appropriateness	Modeling Approach		F1-sc*	F1-mi*	F1-ma*	F1-wei*
							**Type**	**Model name**				
Wu et al. (1)	Main	MIMIC-IV	English	89,098	7,942	Long-tail	**Hybrid**	GoM-ICD	—	0.61	0.33	—
								Gap 0	—	0.61	0.33	—
	Baseline	MIMIC-IV	English	89,098	7,942	Long-tail	**Hybrid**	Gap 1	—	0.60	0.28	—
								Gap 2	—	0.60	0.30	—
								CLH-small	—	0.27	0.18	—
								CLH-base	—	0.38	0.24	—
Motzfeldt et al. (2)	Main	MDACE	English	1,000	1,000	Extreme sparsity	**Hybrid**	CLH-large	—	0.43	0.28	—
								CLH-o3-mini	—	0.37	0.24	—
								CLH-o4-mini	—	0.41	0.27	—
	Baseline	MDACE	English	1,000	1,000	Extreme sparsity	**LLM**	CLH-base	—	0.42	0.27	—
								Llama3-70B	—	0.28	0.18	—
Falis et al. (4)	Main	MIMIC-IV	English	110,442	25,230	Extreme sparsity	**LLM**	GPT-3.5 real	0.15	—	—	—
								CAML base	—	0.54	0.04	—
	Baseline	MIMIC-IV	English	110,442	25,230	Extreme sparsity	**DL**	LAAT base	—	0.57	0.06	—
								MRCNN base	—	0.56	0.06	—
								LAAT real	0.57	—	—	—
	Baseline	MIMIC-IV +GPT-3.5 synthetic	English	120,048	25,230	Long-tail	**DL**	CAML aug	—	0.54	0.04	—
								LAAT aug	—	0.57	0.06	—
								MRCNN aug	—	0.55	0.06	—
Nguyen et al. (5)	Main	MIMIC-IV	English	110,442	26,096	Extreme sparsity	**Hybrid**	PLM-ICD	—	0.57	0.05	—

Continued on next page

Table S12: Continued. Summary of experimental settings and performance across ICD-10 coding studies.

Author et al. (year)	Modeling Role	Dataset name	Language	Training Dataset Size	Number of Labels	Appropriateness	Modeling Approach		F1-sc*	F1-mi*	F1-ma*	F1-wei*
							Type	Model name				
								CAML	—	0.53	0.04	—
	Baseline	MIMIC-IV	English	110,442	26,096	Extreme sparsity	DL	LAAT	—	0.55	0.04	—
								MSMN	—	0.56	0.05	—
	Baseline	MIMIC-IV	English	110,442	26,096	Extreme sparsity	Hybrid	Joint LAAT	—	0.56	0.06	—
								LATA-BERT	—	0.63	—	—
								LATA-ALBERT	—	0.61	—	—
								LATA-DeBERTa	—	0.61	—	—
Mayya et al. (9)	Main	CodiEsp	Mixed: English and Spanish	695	2,194	Extreme sparsity	Hybrid	LATA-DistilBERT	—	0.62	—	—
								LATA-MobileBERT	—	0.27	—	—
								LATA-RoBERTa	—	0.64	—	—
								LATA-XLNet	—	0.63	—	—
								BERT	—	0.38	—	—
								ALBERT	—	0.33	—	—
								DeBERTa	—	0.37	—	—
	Baseline	CodiEsp	Mixed: English and Spanish	695	2,194	Extreme sparsity	TF	DistilBERT	—	0.38	—	—
								MobileBERT	—	0.22	—	—
								RoBERTa	—	0.37	—	—
								XLNet	—	0.31	—	—
Schlegel et al. (11)	Main	OPD-dedup	Mixed: English and Chinese	2,381,000	2,588	Robust learning	Hybrid	OPD-RERANKER	—	0.63	0.21	—

Continued on next page

Table S12: Continued. Summary of experimental settings and performance across ICD-10 coding studies.

Author et al. (year)	Modeling Role	Dataset name	Language	Training Dataset Size	Number of Labels	Appropriateness	Modeling Approach: Type	Modeling Approach: Model name	F1-sc*	F1-mi*	F1-ma*	F1-wei*
	Baseline	OPD-dedup	Mixed: English and Chinese	2,381,000	2,588	Robust learning	**DL**	CAML	—	0.42	0.15	—
								LAAT	—	0.58	0.17	—
								MSMN	—	0.57	0.17	—
	Baseline	OPD-dedup	Mixed: English and Chinese	2,381,000	2,588	Robust learning	**Hybrid**	OPD-LM-LAAT	—	0.62	0.20	—
Lenc et al. (13)	Baseline	MIMIC-IV	English	89,141	7,942	Long-tail	**Hybrid**	mini-PLM-ICD	—	0.56	0.21	—
								Four-headed + CorNet	—	0.56	0.51	—
Polignano et al. (16)	Main	CodiEsp-D	Spanish	500	1,788	Extreme sparsity	**DL**	BiLSTM + CNN + SelfAtt. - BiLSTM + CNN + SelfAtt.	—	—	0.11	—
	Main	CodiEsp-D	Spanish	500	1,788	Extreme sparsity	**TF**	BERT(BETO)-BERT(BETO multi-class)	—	—	0.12	—
	Main	CodiEsp-D	Spanish	500	1,788	Extreme sparsity	**Hybrid**	BiLSTM + CNN + SelfAtt. - BERT (BETO multi-class)	—	—	0.10	—
								BERT (BETO) - BiLSTM + CNN + SelfAtt.	—	—	0.14	—
	Baseline	CodiEsp-D	Spanish	500	1,788	Extreme sparsity	**DL**	LSTM	—	—	0.09	—
								BiLSTM	—	—	0.10	—
								BiLSTM + CNN	—	—	0.10	—
								BiLSTM + CNN + SelfAtt.	—	—	0.10	—
								CNN	—	—	0.09	—
								CNN + SelfAtt.	—	—	0.09	—
	Baseline	CodiEsp-D	Spanish	500	1,788	Extreme sparsity	**TF**	BERT(BETO)	—	—	0.10	—
	Main	CodiEsp-D	Spanish	500	1,788	Extreme sparsity	**Hybrid**	BERT(BETO)-BiLSTM+CNN+SelfAtt.	—	—	0.31	—

Continued on next page

Table S12: Continued. Summary of experimental settings and performance across ICD-10 coding studies.

Author et al. (year)	Modeling Role	Dataset name	Language	Training Dataset Size	Number of Labels	Appropriateness	Modeling Approach: Type	Modeling Approach: Model name	F1-sc*	F1-mi*	F1-ma*	F1-wei*
	Baseline	CodiEsp-D	Spanish	500	1,788	Extreme sparsity	**TF**	BERT(BETO)	—	—	0.24	—
	Baseline	CodiEsp-D	Spanish	500	1,788	Extreme sparsity	**DL**	BiLSTM + CNN + SelfAtt.	—	—	0.21	—
Hauksson and Einarsson (17)	Main	Ours	Icelandic	14,400,000	8,922	Extremely strong	**TF**	MedIceBERT	—	0.66	0.18	—
Bou Hatoum et al. (18)	Main	All codes	English	9,570,000	3,100	Extremely strong	**Hybrid**	ClinicalBERT + TLCL	—	0.86	—	0.84
								ClinicalBERT + PLCL	—	0.84	—	0.82
								ClinicalBERT + BHML	—	0.87	—	0.84
								ClinicalBERT + WHML @0.25	—	0.86	—	0.85
								ClinicalBERT + WHML @0.75	—	0.89	—	0.87
	Baseline	All codes	English	9,570,000	3,100	Extremely strong	**Hybrid**	ClinicalBERT+Binary Cross-Entropy, EM	—	0.75	—	0.72
Xu and Dray (19)	Main	Centralized Learning	English	84,941	1,085	Acceptable	**Hybrid**	Qwen3-Embed-0.6B + DeepMLP	—	0.36	0.23	—
								Qwen3-Embed-0.6B + DeepResMLP	—	0.39	0.27	—
								gte-Qwen2-1.5B-instruct + DeepMLP	—	0.37	0.22	—
								gte-Qwen2-1.5B-instruct + DeepResMLP	—	0.39	0.27	—
								gte-multilingual-base + DeepMLP	—	0.36	0.22	—
								gte-multilingual-base + DeepResMLP	—	0.38	0.26	—
								snowflake-arctic-embed-l-v2.0 + DeepMLP	—	0.37	0.20	—
								snowflake-arctic-embed-l-v2.0 + DeepResMLP	—	0.39	0.25	—
								snowflake-arctic-embed-m-v2.0 + DeepMLP	—	0.36	0.21	—
								snowflake-arctic-embed-m-v2.0 + DeepResMLP	—	0.38	0.25	—
								nomic-embed-text-v1.5 + DeepMLP	—	0.35	0.19	—
								nomic-embed-text-v1.5 + DeepResMLP	—	0.36	0.23	—

Continued on next page

Table S12: Continued. Summary of experimental settings and performance across ICD-10 coding studies.

Author et al. (year)	Modeling Role	Dataset name	Language	Training Dataset Size	Number of Labels	Appropriateness	Modeling Approach		F1-sc*	F1-mi*	F1-ma*	F1-wei*
							Type	Model name				
	Baseline	Centralized Learning	English	84,941	1,085	Acceptable	**Hybrid**	Qwen3-Embed-0.6B + MLP	—	0.39	0.27	—
								gte-Qwen2-1.5B-instruct + MLP	—	0.40	0.27	—
								gte-multilingual-base + MLP	—	0.39	0.26	—
								snowflake-arctic-embed-l-v2.0 + MLP	—	0.338	0.25	—
								snowflake-arctic-embed-m-v2.0 + MLP	—	0.37	0.25	—
								nomic-embed-text-v1.5 + MLP	—	0.36	0.23	—
	Main	Federated learning	English	84,941	1,085	Acceptable	**Hybrid**	Qwen3-Embed-0.6B + DeepMLP	—	0.36	0.19	—
								Qwen3-Embed-0.6B + DeepResMLP	—	0.39	0.25	—
								gte-Qwen2-1.5B-instruct + DeepMLP	—	0.36	0.19	—
								gte-Qwen2-1.5B-instruct + DeepResMLP	—	0.37	0.26	—
								gte-multilingual-base + DeepMLP	—	0.35	0.19	—
								gte-multilingual-base + DeepResMLP	—	0.35	0.25	—
								snowflake-arctic-embed-l-v2.0 + DeepMLP	—	0.36	0.18	—
								snowflake-arctic-embed-l-v2.0 + DeepResMLP	—	0.36	0.23	—
								snowflake-arctic-embed-m-v2.0 + DeepMLP	—	0.35	0.18	—
								snowflake-arctic-embed-m-v2.0 + DeepResMLP	—	0.34	0.22	—
								nomic-embed-text-v1.5 + DeepMLP	—	0.29	0.16	—
								nomic-embed-text-v1.5 + DeepResMLP	—	0.31	0.18	—
	Baseline	Federated learning	English	84,941	1,085	Acceptable	**Hybrid**	Qwen3-Embed-0.6B + MLP	—	0.39	0.26	—
								gte-Qwen2-1.5B-instruct + MLP	—	0.40	0.26	—
								gte-multilingual-base + MLP	—	0.36	0.25	—
								snowflake-arctic-embed-l-v2.0 + MLP	—	0.39	0.24	—
								snowflake-arctic-embed-m-v2.0 + MLP	—	0.36	0.23	—
								nomic-embed-text-v1.5 + MLP	—	0.34	0.21	—

Continued on next page

Table S12: Continued. Summary of experimental settings and performance across ICD-10 coding studies.

Author et al. (year)	Modeling Role	Dataset name	Language	Training Dataset Size	Number of Labels	Appropriateness	Modeling Approach: Type	Modeling Approach: Model name	F1-sc*	F1-mi*	F1-ma*	F1-wei*
Makohon and Li (20)	Main	MIMIC-III (ICD-10 mapped)	English	36,905	5,891	Long-tail	**DL**	LSTM Attn	—	0.61	0.38	—
								GRU Attn	—	0.72	0.53	—
								HAN LSTM	—	0.64	0.42	—
								HAN GRU	—	0.73	0.56	—
	Baseline	MIMIC-III (ICD-10 mapped)	English	36,905	5,891	Long-tail	**DL**	LSTM	—	0.60	0.37	—
								GRU	—	0.59	0.37	—
	Main	MIMIC-III (ICD-10 mapped)+CodiEsp	Mixed: English and Spanish	41,951	7,658	Long-tail	**DL**	LSTM Attn	—	0.70	0.50	—
								GRU Attn	—	0.71	0.52	—
								HAN LSTM	—	0.69	0.49	—
								HAN GRU	—	0.73	0.56	—
	Baseline	MIMIC-III (ICD-10 mapped)+CodiEsp	Mixed: English and Spanish	41,951	7,658	Long-tail	**DL**	LSTM	—	0.58	0.37	—
								GRU	—	0.59	0.36	—
Wu et al. (22)	Main	Xiangya (full)	Chinese	6,186	1,177	Long-tail	**Hybrid**	LD-PLAM	—	0.81	—	—
	Baseline	Xiangya (full)	Chinese	6,186	1,177	Long-tail	**DL**	DeepLabeler	—	0.61	—	—
								KAICD	—	0.65	—	—
								CAML	—	0.73	—	—
								MultiResCNN	—	0.74	—	—
	Baseline	Xiangya (full)	Chinese	6,186	1,177	Long-tail	**ML**	MVC-LDA	—	0.71	—	—
								MVC-RLDA	—	0.70	—	—
	Baseline	Xiangya (full)	Chinese	6,186	1,177	Long-tail	**Hybrid**	DR-CAML	—	0.71	—	—
Dalloux et al. (24)	Main	maps 100/100	French	22,400	6,116	Extreme sparsity	**DL**	CNN	0.32	—	—	—

Continued on next page

Table S12: Continued. Summary of experimental settings and performance across ICD-10 coding studies.

**Author et al. (year)**	**Modeling Role**	**Dataset name**	**Language**	**Training Dataset Size**	**Number of Labels**	**Appropriateness**	**Modeling Approach**		**F1-sc***	**F1-mi***	**F1-ma***	**F1-wei***
							**Type**	**Model name**				
	Main	maps 100/1,000	French	22,400	6,116	Extreme sparsity	**DL**	CNN	0.33	—	—	—
	Main	maps 500/100	French	22,400	6,116	Extreme sparsity	**DL**	CNN	0.36	—	—	—
	Main	maps 500/1,000	French	22,400	6,116	Extreme sparsity	**DL**	CNN	0.38	—	—	—
	Main	maps 1,000/100	French	22,400	6,116	Extreme sparsity	**DL**	CNN	0.35	—	—	—
	Main	maps 1,000/1,000	French	22,400	6,116	Extreme sparsity	**DL**	CNN	0.39	—	—	—
	Main	maps 2,000/2,000	French	22,400	6,116	Extreme sparsity	**DL**	CNN	0.40	—	—	—
	Baseline	maps-/600	French	22,400	6,116	Extreme sparsity	**DL**	BiLSTM	0.23	—	—	—

F1-sc* = F1-score, F1-mi* = F1-micro, F1-ma* = F1-macro, F1-wei* = F1-weighted.

Include criteria: All codes, Full codes, Number of labels ≥1,000 labels, Main model, Baseline model.

Exclude criteria: Block level, Block codes, 3-char, 4-char, Number of labels < 1,000 labels.

Hybrid = Hybrid Deep Learning, DL = Deep Learning, ML = Machine Learning, TF = Transformer.

Table S13: Summary of experimental settings and performance across ICD-10 coding studies. (Labels < 1,000)

**Author et al. (year)**	**Modeling Role**	**Dataset name**	**Language**	**Training Dataset Size**	**Number of Labels**	**Appropriateness**	**Modeling Approach**		**F1-score**	**F1-micro**	**F1-macro**	**F1-weighted**
							**Type**	**Model name**				
Motzfeldt et al. (2)	Baseline	MIMIC-III	English	7,500	50	Robust learning	**LLM**	Llama-3.2-1B	—	0.40	0.24	—
	Baseline	MIMIC-III	English	7,500	50	Robust learning	**Hybrid***	Llama-3.2-1B + Label-wise attention	—	0.71	0.65	—
Gao et al. (3)	Main	APH-50	Chinese	5,708	50	Robust learning	**Hybrid**	CliniCoCo	—	0.84	0.80	—
	Baseline	APH-50	Chinese	5,708	50	Robust learning	**DL**	CAML	—	0.75	0.67	—
								LAAT	—	0.80	0.76	—
	Baseline	APH-50	Chinese	5,708	50	Robust learning	**Hybrid**	Joint LAAT	—	0.81	0.76	—
	Baseline	APH-50	Chinese	5,708	50	Robust learning	**TF**	BERT-based model (Chen et al.)	—	0.83	0.76	—
	Main	HPH-50	Chinese	6,134	50	Robust learning	**Hybrid**	CliniCoCo	—	0.84	0.81	—
	Baseline	HPH-50	Chinese	6,134	50	Robust learning	**DL**	CAML	—	0.75	0.68	—
								LAAT	—	0.81	0.77	—
	Baseline	HPH-50	Chinese	6,134	50	Robust learning	**Hybrid**	Joint LAAT	—	0.82	0.77	—
	Baseline	HPH-50	Chinese	6,134	50	Robust learning	**TF**	BERT-based model (Chen et al.)	—	0.83	0.77	—
	Main	APH-100	Chinese	8,462	100	Acceptable	**Hybrid**	CliniCoCo	—	0.80	0.74	—
	Baseline	APH-100	Chinese	8,462	100	Acceptable	**DL**	CAML	—	0.75	0.64	—
								LAAT	—	0.78	0.71	—

Continued on next page

Table S13: Continued. Summary of experimental settings and performance across ICD-10 coding studies. (Labels < 1,000)

Author et al. (year)	Modeling Role	Dataset name	Language	Training Dataset Size	Number of Labels	Appropriateness	Modeling Approach: Type	Modeling Approach: Model name	F1-score	F1-micro	F1-macro	F1-weighted
	Baseline	APH-100	Chinese	8,462	100	Acceptable	**Hybrid**	Joint LAAT	—	0.79	0.71	—
	Baseline	APH-100	Chinese	8,462	100	Acceptable	**TF**	BERT-based model (Chen et al.)	—	0.79	0.71	—
	Main	HPH-100	Chinese	6,409	100	Acceptable	**Hybrid**	CliniCoCo	—	0.82	0.75	—
	Baseline	HPH-100	Chinese	6,409	100	Acceptable	**DL**	CAML	—	0.75	0.64	—
								LAAT	—	0.80	0.72	—
	Baseline	HPH-100	Chinese	6,409	100	Acceptable	**Hybrid**	Joint LAAT	—	0.80	0.72	—
	Baseline	HPH-100	Chinese	6,409	100	Acceptable	**TF**	BERT-based model (Chen et al.)	—	0.81	0.72	—
	Main	APH-full	Chinese	8,462	579	Long-tail	**Hybrid**	CliniCoCo	—	0.73	0.20	—
	Main	HPH-full	Chinese	6,409	671	Long-tail	**Hybrid**	CliniCoCo	—	0.77	0.25	—
Falis et al. (4)	Main	GPT-3.5 synthetic	English	0	195	Long-tail	**LLM**	GPT-3.5 synthetic	0.48	—	—	—
Nguyen et al. (5)	Main	MIMIC-IV	English	104,077	50	Extremely strong	**Hybrid**	PLM-ICD	—	0.73	0.69	—
	Baseline	MIMIC-IV	English	104,077	50	Extremely strong	**DL**	CAML	—	0.68	0.64	—
								LAAT	—	0.73	0.68	—
								MSMN	—	0.74	0.70	—
	Baseline	MIMIC-IV	English	104,077	50	Extremely strong	**Hybrid**	Joint LAAT	—	0.73	0.68	—
Ngo et al. (6)	Main	All codes	Norwegian	70,350	500	Robust learning	**TF**	NorDeClin-BERT-large-NorICD	—	—	—	0.54
		Top 80%		70,350	350	Robust learning		NorBERT3-large-NorICD	—	—	—	0.50

Continued on next page

Table S13: Continued. Summary of experimental settings and performance across ICD-10 coding studies. (Labels < 1,000)

Author et al. (year)	Modeling Role	Dataset name	Language	Training Dataset Size	Number of Labels	Appropriateness	Modeling Approach		F1-score	F1-micro	F1-macro	F1-weighted
							Type	Model name				
	Baseline	All codes	Norwegian	70,350	500	Robust learning	TF	NorDeClin-BERT-base-NorICD	—	—	—	0.50
								NorBERT3-base-NorICD	—	—	—	0.46
								SweDeClin-BERT-SweICD	—	—	—	0.27
								SweDeClin-BERT-NorICD	—	—	—	0.46
								ScandiBERT-NorICD	—	—	—	0.45
								NorBERT3-base-NorICD	—	—	—	0.50
	Baseline	Top 80%	Norwegian	70,350	350	Robust learning	TF	NorDeClin-BERT-base-NorICD	—	—	—	0.58
								NorDeClin-BERT-SweICD	—	—	—	0.31
								SweDeClin-BERT-NorICD	—	—	—	0.54
								ScandiBERT-NorICD	—	—	—	0.54
								NorBERT3-base-NorICD	—	—	—	0.57
Ponthongmak et al. (8)	Main	Rama-data	Thai to Eng	9,197	50	Robust learning	DL	CNN-PubMedBERT	—	0.62	0.54	—
	Main	MIMIC (Predict.)	English	24,554	50	Robust learning	DL	CNN-PubMedBERT	—	0.38	0.12	—
	Main	MIMIC (Finetune)	English	24,554	50	Robust learning	DL	CNN-PubMedBERT	—	0.56	0.43	—
	Baseline	Rama-data	Thai to Eng	9,197	50	Robust learning	ML	NB-TF-IDF	—	0.48	0.33	—
	Baseline	Rama-data	Thai to Eng	9,197	50	Robust learning	DL	CNN-NWE	—	0.64	0.54	—
								CNN-PB	—	0.62	0.54	—
Masud et al. (10)	Main	Cardiology	English	1	148	Acceptable	DL	Deep-ADCA	0.78	—	—	—
	Main	Metabolism	English	3,486	307	Long-tail	DL	Deep-ADCA	0.75	—	—	—

Continued on next page

Table S13: Continued. Summary of experimental settings and performance across ICD-10 coding studies. (Labels < 1,000)

Author et al. (year)	Modeling Role	Dataset name	Language	Training Dataset Size	Number of Labels	Appropriateness	Modeling Approach: Type	Modeling Approach: Model name	F1-score	F1-micro	F1-macro	F1-weighted
	Main	Psychiatry	English	2,510	475	Long-tail	**DL**	Deep-ADCA	0.64	—	—	—
	Main	Nephrology	English	3,336	432	Long-tail	**DL**	Deep-ADCA	0.62	—	—	—
	Main	Neurology	English	5,424	282	Long-tail	**DL**	Deep-ADCA	0.61	—	—	—
Palacios et al. (15)	Main	MIMIC-IV	English	7,368	50	Robust learning	**LLM**	Falcon-RW-1B	—	0.60	0.56	0.61
								Meditron-7B	—	0.68	0.65	0.69
								Phi3-Mini-4K-Instruct-3.5B	—	0.70	0.68	0.72
Polignano et al. (16)	Main	CodiEsp-D	Spanish	500	546	Extreme sparsity	**Hybrid**	BERT - BiLSTM + CNN + SelfAtt.	—	—	0.25	—
	Baseline	CodiEsp-D	Spanish	500	546	Extreme sparsity	**DL**	BiLSTM	—	—	0.19	—
								BiLSTM + CNN + SelfAtt.	—	—	0.16	—
	Baseline	CodiEsp-D	Spanish	500	546	Extreme sparsity	**TF**	BERT	—	—	0.21	—
Hauksson and Einarsson (17)	Main	Ours (50)	Icelandic	14,400,000	50	Extremely strong	**TF**	MedIceBERT	—	0.75	0.68	—
	Main	Ours (100)	Icelandic	14,400,000	100	Extremely strong	**TF**	MedIceBERT	—	0.72	0.65	—

Continued on next page

Table S13: Continued. Summary of experimental settings and performance across ICD-10 coding studies. (Labels < 1,000)

**Author et al. (year)**	**Modeling Role**	**Dataset name**	**Language**	**Training Dataset Size**	**Number of Labels**	**Appropriateness**	**Modeling Approach: Type**	**Modeling Approach: Model name**	**F1-score**	**F1-micro**	**F1-macro**	**F1-weighted**
Xu and Dray (19)	Main	MIMIC-IV (10)	English	84,941	10	Extremely strong	**Hybrid**	Qwen3-Embed-0.6B + DeepMLP	0.59	—	—	—
								Qwen3-Embed-0.6B + DeepResMLP	0.60	—	—	—
								gte-Qwen2-1.5B-instruct + DeepMLP	0.61	—	—	—
								gte-Qwen2-1.5B-instruct + DeepResMLP	0.62	—	—	—
								gte-multilingual-base + DeepMLP	0.58	—	—	—
								gte-multilingual-base + DeepResMLP	0.59	—	—	—
								snowflake-arctic-embed-l-v2.0 + DeepMLP	0.61	—	—	—
								snowflake-arctic-embed-l-v2.0 + DeepResMLP	0.62	—	—	—
								snowflake-arctic-embed-m-v2.0 + DeepMLP	0.59	—	—	—
								snowflake-arctic-embed-m-v2.0 + DeepResMLP	0.61	—	—	—
								nomic-embed-text-v1.5 + DeepMLP	0.57	—	—	—
	Baseline	MIMIC-IV (10)	English	84,941	10	Extremely strong	**Hybrid**	Qwen3-Embed-0.6B + MLP	0.59	—	—	—
								gte-Qwen2-1.5B-instruct + MLP	0.61	—	—	—
								gte-multilingual-base + MLP	0.58	—	—	—
								snowflake-arctic-embed-l-v2.0 + MLP	0.62	—	—	—
								snowflake-arctic-embed-m-v2.0 + MLP	0.60	—	—	—
								nomic-embed-text-v1.5 + MLP	0.58	—	—	—

Continued on next page

Table S13: Continued. Summary of experimental settings and performance across ICD-10 coding studies. (Labels < 1,000)

Author et al. (year)	Modeling Role	Dataset name	Language	Training Dataset Size	Number of Labels	Appropriateness	Modeling Approach: Type	Modeling Approach: Model name	F1-score	F1-micro	F1-macro	F1-weighted
	Main	MIMIC-IV (50)	English	84,941	50	Extremely strong	**Hybrid**	Qwen3-Embed-0.6B + DeepMLP	0.44	—	—	—
								Qwen3-Embed-0.6B + DeepResMLP	0.46	—	—	—
								gte-Qwen2-1.5B-instruct + DeepMLP	0.46	—	—	—
								gte-Qwen2-1.5B-instruct + DeepResMLP	0.48	—	—	—
								gte-multilingual-base + DeepMLP	0.43	—	—	—
								gte-multilingual-base + DeepResMLP	0.44	—	—	—
								snowflake-arctic-embed-l-v2.0 + DeepMLP	0.46	—	—	—
								snowflake-arctic-embed-l-v2.0 + DeepResMLP	0.48	—	—	—
								snowflake-arctic-embed-m-v2.0 + DeepMLP	0.44	—	—	—
								snowflake-arctic-embed-m-v2.0 + DeepResMLP	0.46	—	—	—
								nomic-embed-text-v1.5 + DeepMLP	0.42	—	—	—
	Baseline	MIMIC-IV (50)	English	84,941	50	Extremely strong	**Hybrid**	Qwen3-Embed-0.6B + MLP	0.44	—	—	—
								gte-Qwen2-1.5B-instruct + MLP	0.45	—	—	—
								gte-multilingual-base + MLP	0.47	—	—	—
								snowflake-arctic-embed-l-v2.0 + MLP	0.43	—	—	—
								snowflake-arctic-embed-m-v2.0 + MLP	0.47	—	—	—
								nomic-embed-text-v1.5 + MLP	0.43	—	—	—
Mustafa et al. (21)	Main	MIMIC-IV	English	1,500	10	Robust learning	**LLM**	Deepseek Reasoner	—	—	0.17	—
								Gemini 2.0 Flash Thinking	—	—	0.04	—
								Gemini 2.5 Pro	—	—	0.24	—
								GPT o3 Mini	—	—	0.08	—
								Qwen QWQ	—	—	0.04	—
Wu et al. (22)	Main	Xiangya 50	Chinese	6,186	50	Robust learning	**Hybrid**	LD-PLAM	—	0.86	—	—

Continued on next page

Table S13: Continued. Summary of experimental settings and performance across ICD-10 coding studies. (Labels < 1,000)

Author et al. (year)	Modeling Role	Dataset name	Language	Training Dataset Size	Number of Labels	Appropriateness	Modeling Approach: Type	Modeling Approach: Model name	F1-score	F1-micro	F1-macro	F1-weighted
	Baseline	Xiangya 50	Chinese	6,186	50	Robust learning	**DL**	DeepLabeler	—	0.75	—	—
								KAICD	—	0.79	—	—
								CAML	—	0.80	—	—
								MultiResCNN	—	0.81	—	—
	Baseline	Xiangya 50	Chinese	6,186	50	Robust learning	**Hybrid**	DR-CAML	—	0.80	—	—
	Baseline	Xiangya 50	Chinese	6,186	50	Robust learning	**ML**	MVC-LDA	—	0.80	—	—
								MVC-RLDA	—	0.80	—	—
Lamproudis et al. (23)	Main	Corpus II	Swedish	72,980	415	Robust learning	**TF**	SweDeClin-BERT	0.61	—	—	—
	Main	Corpus II	Swedish	72,980	69	Extremely strong	**TF**	SweDeClin-BERT	0.71	—	—	—

Include criteria: All codes, Full codes, Number of labels < 1,000 labels, Main model, Baseline model.

Exclude criteria: Block level, Block codes, 3-char, 4-char, Number of labels ≥ 1,000 labels.

Hybrid = Hybrid Deep Learning, DL = Deep Learning, ML = Machine Learning, TF = Transformer, Hybrid* = Hybrid LLM.

Table S14: Summary of experimental settings and performance across ICD-10 coding studies. (3-char)

Author et al. (year)	Modeling Role	Dataset name	Language	Training Dataset Size	Number of Labels	Appropriateness	Modeling Approach Type	Modeling Approach Model name	F1-score	F1-micro	F1-macro	F1-weighted
Vassileva et al. (7)	Main	ElCardioCC	Greek	1,000	2,000	Extreme sparsity	**LLM**	GPT-4o 0-shot	0.59	—	—	—
								GPT-4o 1-shot	0.47	—	—	—
								GPT-4.1 0-shot	0.82	—	—	—
								GPT-4.1 1-shot	0.82	—	—	—
	Main	ElCardioCC	Greek	1,000	2,000	Extreme sparsity	**Hybrid***	Dict+GPT-4o 0-shot	0.78	—	—	—
								Dict+GPT-4o 1-shot	0.73	—	—	—
								Dict+GPT-4.1 0-shot	0.85	—	—	—
								Dict+GPT-4.1 1-shot	0.86	—	—	—
	Baseline	ElCardioCC	Greek	1,000	2,000	Extreme sparsity	**Rule-b**	Dict	0.66	—	—	—
	Main	CodiEsp Cat	Spanish	1,000	2,000	Extreme sparsity	**LLM**	GPT-4o 0-shot	0.64	—	—	—
								GPT-4.1 0-shot	0.20	—	—	—
								GPT-4.1 1-shot	0.77	—	—	—
	Main	CodiEsp Cat	Spanish	1,000	2,000	Extreme sparsity	**Hybrid***	Dict+GPT-4o 0-shot	0.78	—	—	—
								Dict+GPT-4o 1-shot	0.60	—	—	—
								Dict+GPT-4.1 0-shot	0.89	—	—	—
								Dict+GPT-4.1 1-shot	0.89	—	—	—
	Baseline	CodiEsp Cat	Spanish	1,000	2,000	Extreme sparsity	**Rule-b**	Dict	0.55	—	—	—
Mustafa et al. (12)	Main	MIMIC-IV	English	1,500	10	Robust learning	**LLM**	Deepseek Reasoner	—	—	0.31	—
								Gemini 2.0 Flash Thinking	—	—	0.14	—
								Gemini 2.5 Pro	—	—	0.39	—
								GPT o3 Mini	—	—	0.21	—
								Qwen QWQ	—	—	0.22	—

Continued on next page

Table S14: Continued. Summary of experimental settings and performance across ICD-10 coding studies. (3-char)

Author et al. (year)	Modeling Role	Dataset name	Language	Training Dataset Size	Number of Labels	Appropriateness	Modeling Approach: Type	Modeling Approach: Model name	F1-score	F1-micro	F1-macro	F1-weighted
Lenc et al. (13)	Main	Czech AMC	Czech	376,515	1,538	Robust learning	**TF**	mini-transformer	—	0.79	0.45	—
								mini-transformer CLS+SEP	—	0.80	0.51	—
								mini-PLM-ICD	—	0.81	0.54	—
	Main	Czech AMC	Czech	376,515	1,538	Robust learning	**Hybrid**	Four-headed	—	0.80	0.53	—
								Four-headed+CorNet	—	0.80	0.53	—
	Baseline	Czech AMC	Czech	376,515	1,538	Robust learning	**TF**	Small-E-Czech	—	0.74	0.31	—
Suvirat et al. (14)	Main	Top-100 codes	Thai	262,625	8,650	Long-tail	**TF**	Multilingual BERT	—	—	0.88	—
	Baseline	Top-100 codes	Thai	73,405	8,650	Long-tail	**ML**	Bag of n-grams	—	—	0.79	—
	Baseline	Top-100 codes	Thai	170,416	8,650	Long-tail	**ML**	TF-IDF	—	—	0.81	—
	Main	Top-300 codes	Thai	293,361	8,650	Long-tail	**TF**	Multilingual BERT	—	—	0.81	—
	Baseline	Top-300 codes	Thai	118,546	8,650	Long-tail	**ML**	Bag of n-grams	—	—	0.68	—
	Baseline	Top-300 codes	Thai	201,152	8,650	Long-tail	**ML**	TF-IDF	—	—	0.71	—
	Main	Top-500 codes	Thai	324,098	8,650	Long-tail	**TF**	Multilingual BERT	—	—	0.76	—
	Baseline	Top-500 codes	Thai	134,878	8,650	Long-tail	**ML**	Bag of n-grams	—	—	0.62	—
	Baseline	Top-500 codes	Thai	231,889	8,650	Long-tail	**ML**	TF-IDF	—	—	0.65	—
Mustafa et al. (21)	Main	MIMIC-IV	English	3,000	10	Robust learning	**LLM**	Deepseek Chat	—	—	0.70	—
								Gemini 2.0 Flash	—	—	0.76	—
								GPT 3o Mini	—	—	0.63	—
								Qwen QWQ 32B	—	—	0.55	—
Dalloux et al. (24)	Main	maps 1,000/1,000	French	22,400	6,116	Extreme sparsity	**DL**	CNN	0.52	—	—	—
		maps 2,000/2,000		22,400	6,116	Extreme sparsity		CNN	0.52	—	—	—

Continued on next page

Table S14: Continued. Summary of experimental settings and performance across ICD-10 coding studies. (3-char)

Author et al. (year)	Modeling Role	Dataset name	Language	Training Dataset Size	Number of Labels	Appropriateness	Modeling Approach		F1-score	F1-micro	F1-macro	F1-weighted
							**Type**	**Model name**				
	Baseline	maps-/600	French	22,400	6,116	Extreme sparsity	**DL**	BiLSTM	0.39	—	—	—

Include criteria: All codes, 3-char, Main model, Baseline model.

Exclude criteria: Block level, Block codes, Full codes, 4-char.

Hybrid = Hybrid Deep Learning, DL = Deep Learning, ML = Machine Learning, TF = Transformer, Hybrid* = Hybrid LLM, Rule-b = Rule-based.

Table S15: Summary of experimental settings and performance across ICD-10 coding studies. (4-char)

Author et al. (year)	Modeling Role	Dataset name	Language	Training Dataset Size	Number of Labels	Appropriateness	Modeling Approach: Type	Modeling Approach: Model name	F1-score	F1-micro	F1-macro	F1-weighted
Vassileva et al. (7)	Main	CodiEsp Subcat	Spanish	1,000	14,000	Extreme sparsity	**LLM**	GPT-4o 0-shot	0.53	—	—	—
								GPT-4o 1-shot	0.17	—	—	—
								GPT-4.1 0-shot	0.57	—	—	—
								GPT-4.1 1-shot	0.61	—	—	—
	Main	CodiEsp Subcat	Spanish	1,000	14,000	Extreme sparsity	**Hybrid***	Dict+GPT-4o 0-shot	0.53	—	—	—
								Dict+GPT-4o 1-shot	0.60	—	—	—
								Dict+GPT-4.1 0-shot	0.78	—	—	—
								Dict+GPT-4.1 1-shot	0.78	—	—	—
	Baseline	CodiEsp Subcat	Spanish	1,000	14,000	Extreme sparsity	**Rule-b**	Dict	0.53	—	—	—
Mustafa et al. (12)	Main	MIMIC-IV	English	1,500	10	Robust learning	**LLM**	Deepseek Reasoner	—	—	0.20	—
								Gemini 2.0 Flash Thinking	—	—	0.08	—
								Gemini 2.5 Pro	—	—	0.27	—
								GPT o3 Mini	—	—	0.12	—
								Qwen QWQ	—	—	0.10	—
Lenc et al. (13)	Main	Czech AMC	Czech	376,515	6,304	Acceptable	**TF**	mini-transformer	—	0.68	0.21	—
								mini-transformer CLS+SEP	—	0.72	0.31	—
								mini-PLM-ICD	—	0.74	0.36	—
	Main	Czech AMC	Czech	376,515	6,304	Acceptable	**Hybrid**	Four-headed	—	0.74	0.35	—
								Four-headed+CorNet	—	0.74	0.35	—
	Baseline	Czech AMC	Czech	376,515	6,304	Acceptable	**TF**	Small-E-Czech	—	0.62	0.11	—

Include criteria: All codes, 4-char, Main model, Baseline model.

Exclude criteria: Block level, Block codes, Full codes, 3-char.

Hybrid = Hybrid Deep Learning, DL = Deep Learning, ML = Machine Learning, TF = Transformer, Hybrid* = Hybrid LLM, Rule-b = Rule-based.

Table S16: Highest-performing models by F1 metrics.

Metric	Authors	Language	Training data	Labels	Appropriateness	Model	Score
F1-weighted	Bou Hatoum et al. (18)	English	9,570,000	3,100	Extremely strong	**Hybrid** ClinicalBERT + WHML @0.75	0.87
F1-micro	Bou Hatoum et al. (18)	English	9,570,000	3,100	Extremely strong	**Hybrid** ClinicalBERT + WHML @0.75	0.89
F1-macro	Makohon and Li (20)	Mixed: English and Spanish	41,951	7,658	Long-tail	**DL** HAN GRU	0.56

Include criteria: All codes, Full codes.

Exclude criteria: Block level, Block codes, 3-char, 4-char, Top-x codes.

Table S17: Highest-reported performance in *imbalance-aware* ICD-10 coding models.

Metric	Authors	Language	Training data	Labels	Appropriateness	Model	Score
F1-weighted	Bou Hatoum et al. (18)	English	9,570,000	3,100	Extremely strong	**Hybrid** ClinicalBERT + WHML @0.75	0.87
F1-micro	Bou Hatoum et al. (18)	English	9,570,000	3,100	Extremely strong	**Hybrid** ClinicalBERT + WHML @0.75	0.89
F1-macro	Gao et al. (3)	Chinese	6,134	50	Robust learning	**Hybrid** CliniCoCo	0.75

Include criteria: All codes, Full codes, Top-x codes.

Exclude criteria: Block level, Block codes, 3-char, 4-char.

Table S18: Highest-reported model performance in *extreme sparsity* settings.

Metric	Authors	Language	Training data	Labels	Model	Score
F1-weighted	—	—	—	—	—	—
F1-micro	Mayya et al. (2021)	English	695	2,194	**TF** LATA-RoBERTa	0.64
F1-macro	Polignano et al. (16)	Spanish	500	1,788	**Hybrid DL** BERT (BETO) - BiLSTM + CNN + SelfAtt.	0.31

Documents per label: Extreme sparsity (< 5), Long-tail (5–20), Acceptable (20–100), Robust learning (100–1,000), Extremely strong ($> 1,000$).

Include criteria: All codes, Full codes, Extreme sparsity settings.

Exclude criteria: Block level, Block codes, 3-char, 4-char, Top-x codes, Other settings such as Long-tail, Acceptable, Robust Learning, and Extremely Strong.

Table S19: Highest-reported model performance in *long-tail* settings.

Metric	Authors	Language	Training data	Labels	Model	Score
F1-weighted	—	—	—	—	—	—
F1-micro	Wu et al. (22)	Chinese	6,186	1,177	**Hybrid DL** LD-PLAM	0.81
F1-macro	Makohon and Li (20)	English	36,905	5,891	**DL** HAN GRU	0.56
F1-macro	Makohon and Li (20)	Mixed: English and Spanish	41,951	7,658	**DL** HAN GRU	0.56

Documents per label: Extreme sparsity (< 5), Long-tail (5–20), Acceptable (20–100), Robust learning (100–1,000), Extremely strong ($> 1,000$).

Include criteria: All codes, Full codes, Long-tail settings.

Exclude criteria: Block level, Block codes, 3-char, 4-char, Top-x codes, Other settings such as Extreme sparsity, Acceptable, Robust Learning, and Extremely Strong.

Table S20: Highest-reported model performance in *acceptable* settings.

Metric	Authors	Language	Training data	Labels	Model	Score
F1-weighted	—	—	—	—	—	—
F1-micro	Xu and Dray (19)	English	84,941	1,085	**Hybrid DL** gte-Qwen2-1.5B-instruct + MLP	0.40
F1-micro	Xu and Dray (19)	English	84,941	1,085	**Hybrid DL** gte-Qwen2-1.5B-instruct + MLP	0.40
F1-macro	Xu and Dray (19)	English	84,941	1,085	**Hybrid DL** Qwen3-Embed-0.6B + DeepResMLP	0.27
F1-macro	Xu and Dray (19)	English	84,941	1,085	**Hybrid DL** gte-Qwen2-1.5B-instruct + DeepResMLP	0.27
F1-macro	Xu and Dray (19)	English	84,941	1,085	**Hybrid DL** Qwen3-Embed-0.6B + MLP	0.27
F1-macro	Xu and Dray (19)	English	84,941	1,085	**Hybrid DL** gte-Qwen2-1.5B-instruct + MLP	0.27

Documents per label: Extreme sparsity (< 5), Long-tail (5–20), Acceptable (20–100), Robust learning (100–1,000), Extremely strong ($> 1,000$).
Include criteria: All codes, Full codes, Acceptable settings.
Exclude criteria: Block level, Block codes, 3-char, 4-char, Top-x codes, Other settings such as Extreme sparsity, Long-tail, Robust Learning, and Extremely Strong.

Table S21: Highest-reported model performance in *robust learning* settings.

Metric	Authors	Language	Training data	Labels	Model	Score
F1-weighted	Ngo et al. (6)	Norwegian	70,350	500	**TF** NorDeClin-BERT-large-NorICD	0.54
F1-micro	Schlegel et al. (11)	Mixed: English and Chinese	2,381,000	2,588	**Hybrid DL** OPD-RERANKER	0.63
F1-macro	Schlegel et al. (11)	Mixed: English and Chinese	2,381,000	2,588	**Hybrid DL** OPD-RERANKER	0.21

Documents per label: Extreme sparsity (< 5), Long-tail (5–20), Acceptable (20–100), Robust learning (100–1,000), Extremely strong ($> 1,000$).
Include criteria: All codes, Full codes, Robust Learning settings.
Exclude criteria: Block level, Block codes, 3-char, 4-char, Top-x codes, Other settings such as Extreme sparsity, Long-tail, Acceptable, and Extremely Strong.

Table S22: Highest-reported model performance in *extremely strong* settings.

**Metric**	**Authors**	**Language**	**Training data**	**Labels**	**Model**	**Score**
F1-weighted	Bou Hatoum et al. (18)	English	9,570,000	3,100	**Hybrid DL** ClinicalBERT + WHML @0.75	0.87
F1-micro	Bou Hatoum et al. (18)	English	9,570,000	3,100	**Hybrid DL** ClinicalBERT + WHML @0.75	0.89
F1-macro	Hauksson and Einarsson (17)	English	14,400,000	8,922	**TF** MedIceBERT	0.18

Documents per label: Extreme sparsity (< 5), Long-tail (5–20), Acceptable (20–100), Robust learning (100–1,000), Extremely strong ($> 1,000$).

Include criteria: All codes, Full codes, Extremely Strong settings.

Exclude criteria: Block level, Block codes, 3-char, 4-char, Top-x codes, Other settings such as Extreme sparsity, Long-tail, Acceptable, and Robust Learning.

REFERENCES

[1]Wu Y, Qiu W, Zeng M, Chen X, Li M, Zhu H. GoM-ICD: Automatic ICD coding with gap schemes and mixture of experts. *Big Data Mining and Analytics* **8** (2025) 1211–1224. doi:10.26599/bdma.2025.9020019.

[2]Motzfeldt A, Edin J, Christensen CL, Hardmeier C, Maaløe L, Rogers A. Code like humans: A multi-agent solution for medical coding. *arXiv [Preprint]* (2025). doi:10.48550/arxiv.2509.05378.

[3]Gao Y, Chen Y, Wang M, Wu J, Kim Y, Zhou K, et al. Optimising the paradigms of human-AI collaborative clinical coding. *npj Digital Medicine* **7** (2024) 368. doi:10.1038/s41746-024-01363-7.

[4]Falis M, Gema AP, Dong H, Daines L, Basetti S, Holder M, et al. Can GPT-3.5 generate and code discharge summaries? *Journal of the American Medical Informatics Association* **31** (2024) 2284–2293. doi:10.1093/jamia/ocae132.

[5]Nguyen TT, Schlegel V, Kashyap A, Winkler S, Huang SS, Liu JJ, et al. MIMIC-IV-ICD: A new benchmark for extreme multilabel classification. *arXiv [Preprint]* (2023). doi:10.48550/arxiv.2304.13998.

[6]Ngo PD, Tejedor Hernandez MA, Chomutare T, Budrionis A, Svenning TO, Torsvik T, et al. Domain-specific pretraining of NorDeClin-BERT for ICD-10 code prediction in Norwegian clinical texts: Model development and evaluation study. *JMIR AI* **4** (2025) e66153. doi:10.2196/66153.

[7]Vassileva S, Koychev IK, Boytcheva S. Using LLMs for multilingual clinical entity linking to ICD-10. *Proceedings of the 15th International Conference on Recent Advances in Natural Language Processing: Natural Language Processing in the Generative AI Era* (Varna, Bulgaria: INCOMA) (2025), 1304–1308.

[8]Ponthongmak W, Thammasudjarit R, McKay GJ, Attia J, Theera-Ampornpunt N, Thakkinstian A. Development and external validation of automated ICD-10 coding from discharge summaries using deep learning approaches. *Informatics in Medicine Unlocked* **38** (2023) 101227. doi:10.1016/j.imu.2023.101227.

[9]Mayya V, Kamath SS, Sugumaran V. LATA: Label attention transformer architectures for ICD-10 coding of unstructured clinical notes. *2021 IEEE Conference on Computational Intelligence in Bioinformatics and Computational Biology (CIBCB)* (Melbourne, Australia: IEEE) (2021), 1–7. doi:10.1109/CIBCB49929.2021.9562815.

[10]Masud JHB, Shun C, Kuo CC, Islam MM, Yeh CY, Yang HC, et al. Deep-ADCA: Development and validation of deep learning model for automated diagnosis code assignment using clinical notes in electronic medical records. *Journal of Personalized Medicine* **12** (2022) 707. doi:10.3390/jpm12050707.

[11]Schlegel V, Kashyap AR, Nguyen TT, Yang TH, Dwivedi VP, Yin WH, et al. Automated clinical coding for outpatient departments. *arXiv [Preprint]* (2023). doi:10.48550/arxiv.2312.13533.

[12]Mustafa A, Naseem U, Azghadi MR. Can reasoning LLMs enhance clinical document classification? *arXiv [Preprint]* (2025). doi:10.48550/arxiv.2504.08040.

[13]Lenc L, Martínek J, Baloun J, Přibáň P, Prantl M, Taylor SE, et al. Czech medical coding assistant based on transformer networks. *Computers in Biology and Medicine* **178** (2024) 108672. doi:10.1016/j.compbiomed.2024.108672.

[14]Suvirat K, Tanasanchonnakul D, Horsiritham K, Kongkamol C, Ingviya T, Chaichulee S. Automated diagnosis code assignment of Thai free-text clinical notes. *2022 IEEE Asia-Pacific Conference on Computer Science and Data Engineering (CSDE)* (Gold Coast, Australia: IEEE) (2022), 1–6. doi:10.1109/CSDE56538.2022.10089356.

[15]Palacios V, Marxer C, Sunshine B, Staudt Y. Evaluating fine-tuned small language models for ICD-10 diagnosis coding. *2025 IEEE Swiss Conference on Data Science (SDS)* (Zürich, Switzerland: IEEE) (2025), 150–153. doi:10.1109/SDS66131.2025.00028.

[16]Polignano M, Suriano V, Lops P, de Gemmis M, Semeraro G. A study of machine learning models for clinical coding of medical reports at CodiEsp 2020. *Working Notes of CLEF 2020: Conference and Labs of the Evaluation Forum* (Thessaloniki, Greece: CEUR-WS.org) (2020), *CEUR Workshop Proceedings*, vol. 2696, 1–18.

[17]Hauksson HO, Einarsson H. Applications of BERT models towards automation of clinical coding in icelandic. *Findings of the Association for Computational Linguistics: NAACL 2024* (Mexico City, Mexico: Association for Computational Linguistics) (2024), 1956–1967. doi:10.18653/v1/2024.findings-naacl.127.

[18]Bou Hatoum M, Charr JC, Ghaddar A, Guyeux C, Laiymani D. Beyond equality matching: Custom loss functions for semantics-aware ICD-10 coding. *Proceedings of the 17th International Conference on Agents and Artificial Intelligence* (Porto, Portugal: SCITEPRESS - Science and Technology Publications) (2025), 166–174. doi:10.5220/0013101000003890.

[19]Xu B, Dray G. Federated learning for ICD classification with lightweight models and pretrained embeddings. *arXiv [Preprint]* (2025). doi:10.48550/arxiv.2507.03122.

[20]Makohon I, Li Y. Multi-label classification of ICD-10 coding and clinical notes using MIMIC and CodiEsp. *2021 IEEE EMBS International Conference on Biomedical and Health Informatics (BHI)* (Athens, Greece: IEEE) (2021), 1–4. doi:10.1109/BHI50953.2021.9508541.

[21]Mustafa A, Naseem U, Azghadi MR. Evaluating hierarchical clinical document classification using reasoning-based llms. *arXiv [Preprint]* (2025). doi:10.48550/arxiv.2507.03001.

[22]Wu Y, Zeng M, Yu Y, Li M. A pseudo label-wise attention network for automatic ICD coding. *arXiv [Preprint]* (2021). doi:10.48550/arxiv.2106.06822.

[23]Lamproudis A, Svenning TO, Torsvik T, Chomutare T, Budrionis A, Ngo PD, et al. Using a large open clinical corpus for improved ICD-10 diagnosis coding. *AMIA Annual Symposium Proceedings* **2023** (2023) 465–473.

[24]Dalloux C, Claveau V, Cuggia M, Bouzillé G, Grabar N. Supervised learning for the ICD-10 coding of French clinical narratives. *Digital Personalized Health and Medicine: Proceedings of MIE 2020* (IOS Press) (2020), *Studies in Health Technology and Informatics*, vol. 270, 427–431. doi:10.3233/SHTI200196.
